# Supplementary material for: Single-nucleus RNA sequencing in ischemic cardiomyopathy reveals common transcriptional profile underlying end-stage heart failure
Source: Cell Rep. Author manuscript; Available in PMC 2023 Oct 23. (PMC10423750; doi:10.1016/j.celrep.2023.112086)
Supplement: 1 [file NIHMS1878882-supplement-1.pdf]

Cell Reports, Volume *42*

Supplemental information

Single-nucleus RNA sequencing in ischemic cardiomyopathy reveals common transcriptional profile underlying end-stage heart failure

Bridget Simonson, Mark Chaffin, Matthew C. Hill, Ondine Atwa, Yasmine Guedira, Harshit Bhasin, Amelia W. Hall, Sikander Hayat, Simon Baumgart, Kenneth C. Bedi Jr., Kenneth B. Margulies, Carla A. Klattenhoff, and Patrick T. Ellinor

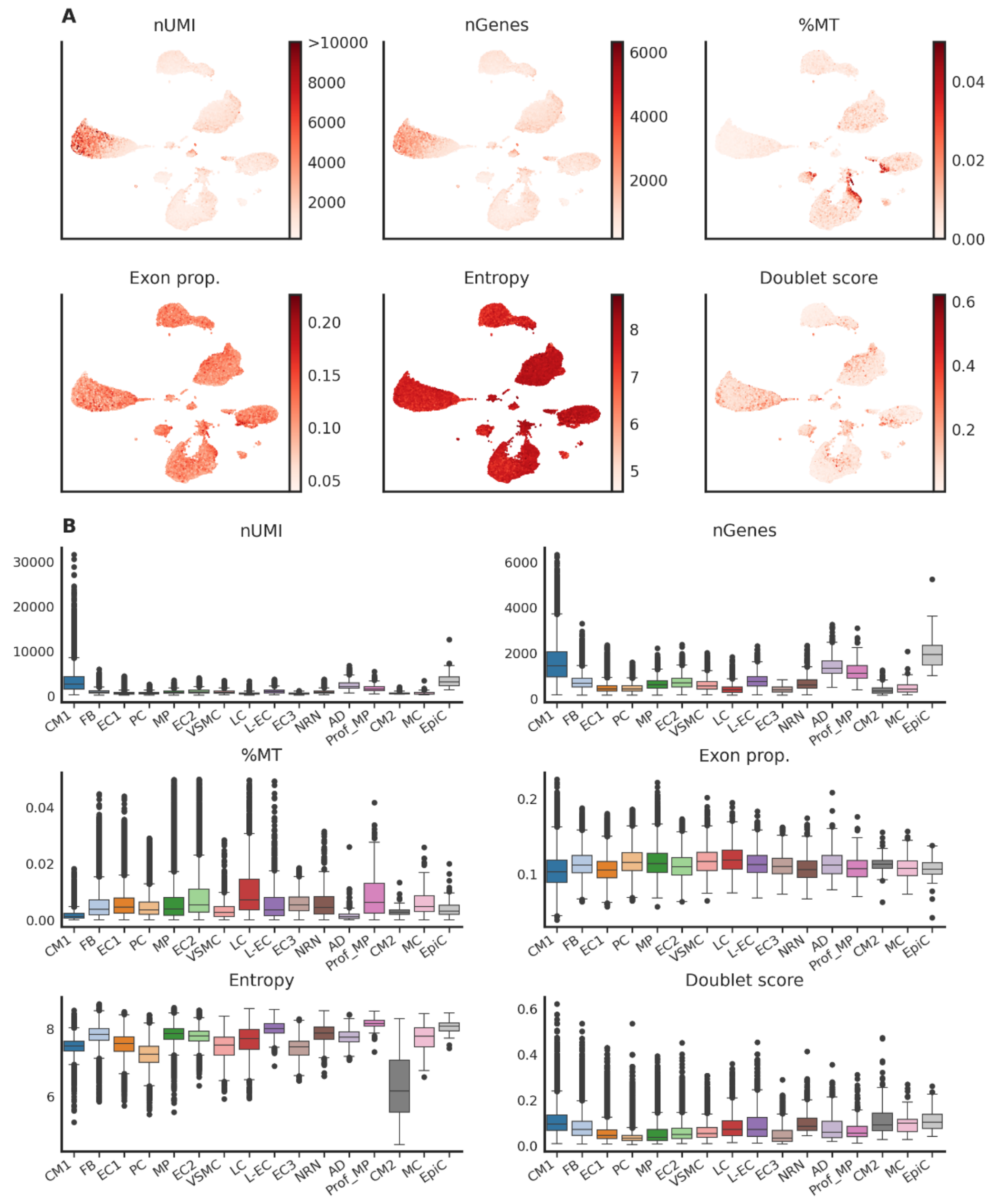

Supplemental Figure 1. Quality control metrics across final snRNA-seq map, related to Figure 1. **A,** Overlay of six per-nucleus quality control (QC) metrics on the final UMAP representation of the snRNA-seq data. QC metrics include the total number of unique molecular identifiers per nucleus (nUMI), the total unique genes detected per nucleus (nGenes), the percent of reads mapping to mitochondrial genes (%MT), the proportion of reads mapping exclusively to exons (Exon prop.), nucleus-level entropy (Entropy), and an estimated doublet score based on Scrublet (Doublet score). **B,** Distribution of the same QC metrics as in **A** stratified by cell type as shown in box plots represented as: Center line, median; Box limits, upper and lower quartiles; Whiskers, 1.5x interquartile range; Points, outliers. CM, cardiomyocyte; FB, fibroblast; EC, endothelial cell; PC, pericyte; MP, macrophage; VSMC, vascular smooth muscle cell; LC, lymphocyte; L-EC, lymphatic endothelial cell; NRN, neuronal; AD, adipocyte; Prof_MP, proliferating macrophage; MC, mast cell; EpiC, epicardial.

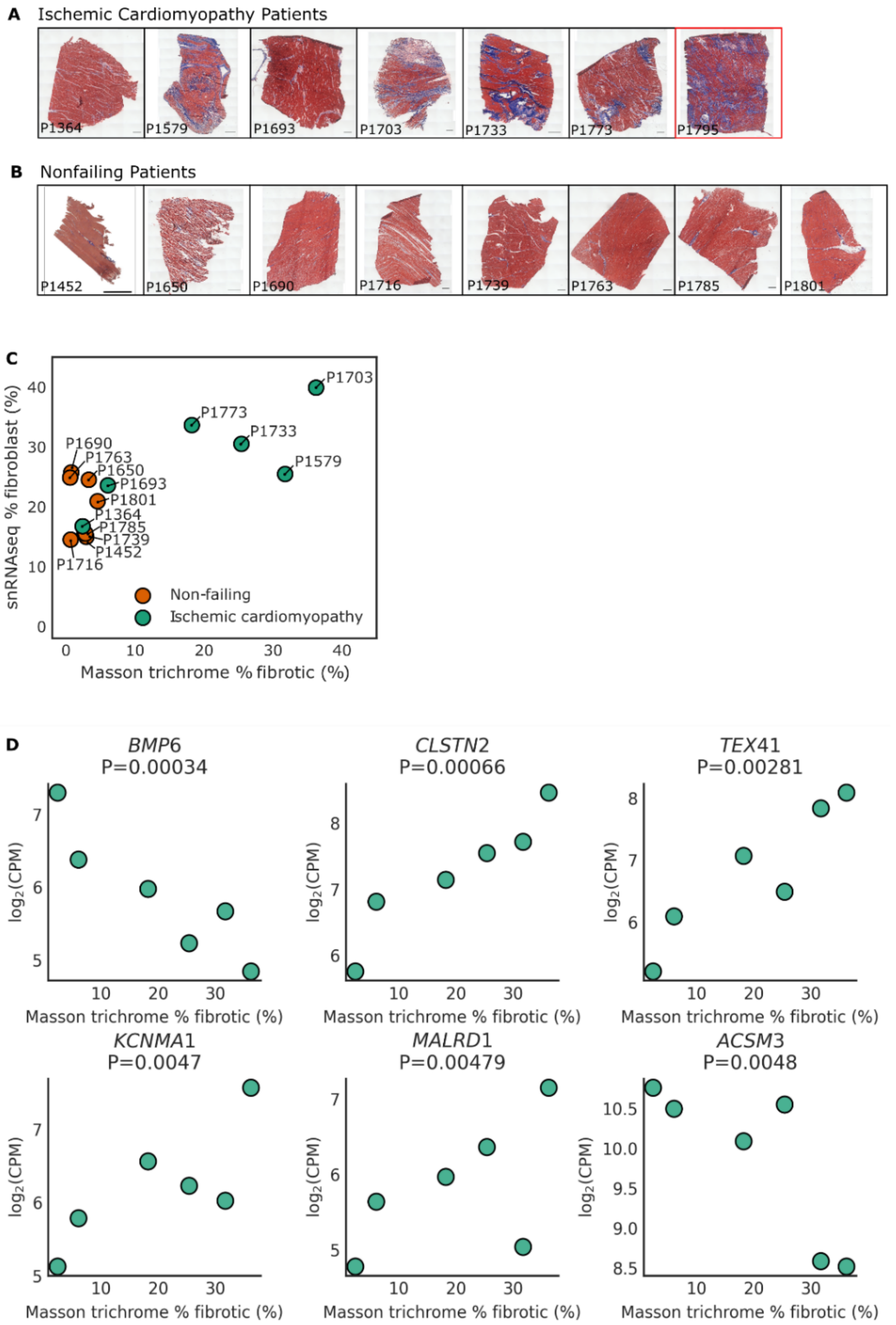

Supplemental Figure 2: LV sections from ICM patients show a wide range of fibrosis compared to NF controls, related to Figure 1. A, B. Mason's Trichrome staining of sections from patient tissue used for snRNAseq. Sample P1795 is denoted with a red border as this sample failed single nuclei RNA-sequencing. Blue: Collagen, Pink: tissue. Scale bar = 1 mm. **C**. Correlation of percentage of tissue section that stained blue with number of fibroblast nuclei from snRNAseq. **D.** Association of fibroblast gene expression with degree of fibrosis seen in Mason's Trichrome staining across 6 ICM patients. Genes with P < 0.005 are shown. CPM, counts per million.

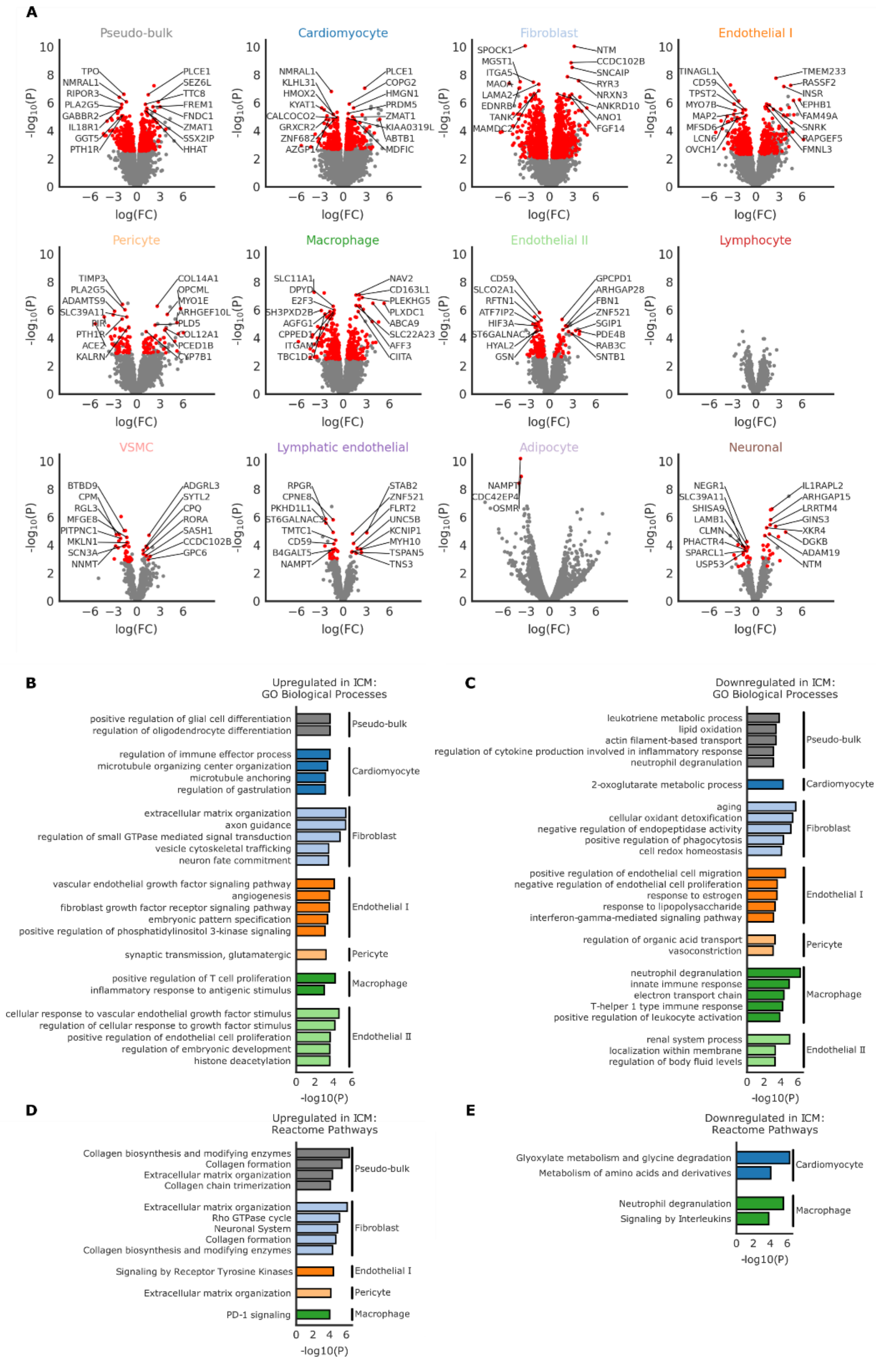
A
Pseudo-bulk
TPO
NMRAL1
RIPOR3
PLA2G5
GABBR2
IL18R1
GGT5
PTH1R
PLCE1
SEZ6L
TTC8
FREM1
FNDC1
ZMAT1
SSX2IP
HHAT
Cardiomyocyte
NMRAL1
KLHL31
HMOX2
KYAT1
CALCOCO2
GRXCR2
ZNF682
AZGP1
PLCE1
COPG2
HMGN1
PRDM5
ZMAT1
KIAA0319L
ABTB1
MDFIC
Fibroblast
SPOCK1
MGST1
ITGA5
MAOA
LAMA2
EDNRB
TANK
MAMDC2
NTM
CCDC102B
SNCAIP
RYR3
NRXN3
ANKRD10
ANO1
FGF14
Endothelial I
TINAGL1
CD59
TPST2
MYO7B
MAP2
MFSD6
LCN6
OVCH1
TMEM233
RASSF2
INSR
EPHB1
FAM49A
SNRK
RAPGEF5
FMNL3
Pericyte
TIMP3
PLA2G5
ADAMTS9
SLC39A11
RIR
PTH1R
ACE2
KALRN
COL14A1
OPCML
MYO1E
ARHGEF10L
PLD5
COL12A1
PCED1B
CYP7B1
Macrophage
SLC11A1
DPYD
E2F3
SH3PXD2B
AGFG1
CPPED1
ITGAM
TBC1D2
NAV2
CD163L1
PLEKHG5
PLXDC1
ABCA9
SLC22A23
AFF3
CIITA
Endothelial II
CD59
SLCO2A1
RFTN1
ATF7IP2
HIF3A
ST6GALNAC3
HYAL2
GSN
GPCPD1
ARHGAP28
FBN1
ZNF521
SGIP1
PDE4B
RAB3C
SNTB1
Lymphocyte
VSMC
BTBD9
CPM
RGL3
MFGE8
PITPNC1
MKLN1
SCN3A
NNMT
ADGRL3
SYTL2
CPQ
RORA
SASH1
CCDC102B
GPC6
Lymphatic endothelial
RPGR
CPNE8
PKHD1L1
ST6GALNAC3
TMTC1
CD59
B4GALT5
NAMPT
STAB2
ZNF521
FLRT2
UNC5B
KCNIP1
MYH10
TSPAN5
TNS3
Adipocyte
NAMPT
CDC42EP4
OSMR
Neuronal
NEGR1
SLC39A11
SHISA9
LAMB1
CLMN
PHACTR4
SPARCL1
USP53
IL1RAPL2
ARHGAP15
LRRTM4
GINS3
XKR4
DGKB
ADAM19
NTM
$-\log_{10}(P)$
log(FC)
B
Upregulated in ICM:
GO Biological Processes
positive regulation of glial cell differentiation
regulation of oligodendrocyte differentiation
Pseudo-bulk
regulation of immune effector process
microtubule organizing center organization
microtubule anchoring
regulation of gastrulation
Cardiomyocyte
extracellular matrix organization
axon guidance
regulation of small GTPase mediated signal transduction
vesicle cytoskeletal trafficking
neuron fate commitment
Fibroblast
vascular endothelial growth factor signaling pathway
angiogenesis
fibroblast growth factor receptor signaling pathway
embryonic pattern specification
positive regulation of phosphatidylinositol 3-kinase signaling
Endothelial I
synaptic transmission, glutamatergic
Pericyte
positive regulation of T cell proliferation
inflammatory response to antigenic stimulus
Macrophage
cellular response to vascular endothelial growth factor stimulus
regulation of cellular response to growth factor stimulus
positive regulation of endothelial cell proliferation
regulation of embryonic development
histone deacetylation
Endothelial II
-log10(P)
C
Downregulated in ICM:
GO Biological Processes
leukotriene metabolic process
lipid oxidation
actin filament-based transport
regulation of cytokine production involved in inflammatory response
neutrophil degranulation
Pseudo-bulk
2-oxoglutarate metabolic process
Cardiomyocyte
aging
cellular oxidant detoxification
negative regulation of endopeptidase activity
positive regulation of phagocytosis
cell redox homeostasis
Fibroblast
positive regulation of endothelial cell migration
negative regulation of endothelial cell proliferation
response to estrogen
response to lipopolysaccharide
interferon-gamma-mediated signaling pathway
Endothelial I
regulation of organic acid transport
vasoconstriction
Pericyte
neutrophil degranulation
innate immune response
electron transport chain
T-helper 1 type immune response
positive regulation of leukocyte activation
Macrophage
renal system process
localization within membrane
regulation of body fluid levels
Endothelial II
-log10(P)
D
Upregulated in ICM:
Reactome Pathways
Collagen biosynthesis and modifying enzymes
Collagen formation
Extracellular matrix organization
Collagen chain trimerization
Pseudo-bulk
Extracellular matrix organization
Rho GTPase cycle
Neuronal System
Collagen formation
Collagen biosynthesis and modifying enzymes
Fibroblast
Signaling by Receptor Tyrosine Kinases
Endothelial I
Extracellular matrix organization
Pericyte
PD-1 signaling
Macrophage
-log10(P)
E
Downregulated in ICM:
Reactome Pathways
Glyoxylate metabolism and glycine degradation
Metabolism of amino acids and derivatives
Cardiomyocyte
Neutrophil degranulation
Signaling by Interleukins
Macrophage
-log10(P)

S**upplemental Figure 3: Differentially expressed genes and pathway analysis of differentially expressed genes in ICM compared to NF, by cell type. Related to Figure 1. A** Volcano plot of differentially expressed genes in cell types, top 8 up- and down-regulated protein coding genes labelled. Genes significant at FDR < 0.05 with a low probability of being derived by background are colored red. **B** Pathway enrichment (P<0.001) for upregulated and downregulated (**C**) genes in ICM compared to NF based on gene ontology (GO) biological processes database using TopGO. **D** Reactome pathway enrichment (FDR<0.05) for upregulated and downregulated (**E**) genes in ICM compared to NF based on Reactome Pathways database using ReactomePA. log(FC), log fold-change; VSMC, vascular smooth muscle cell; ICM, Ischemic cardiomyopathy.

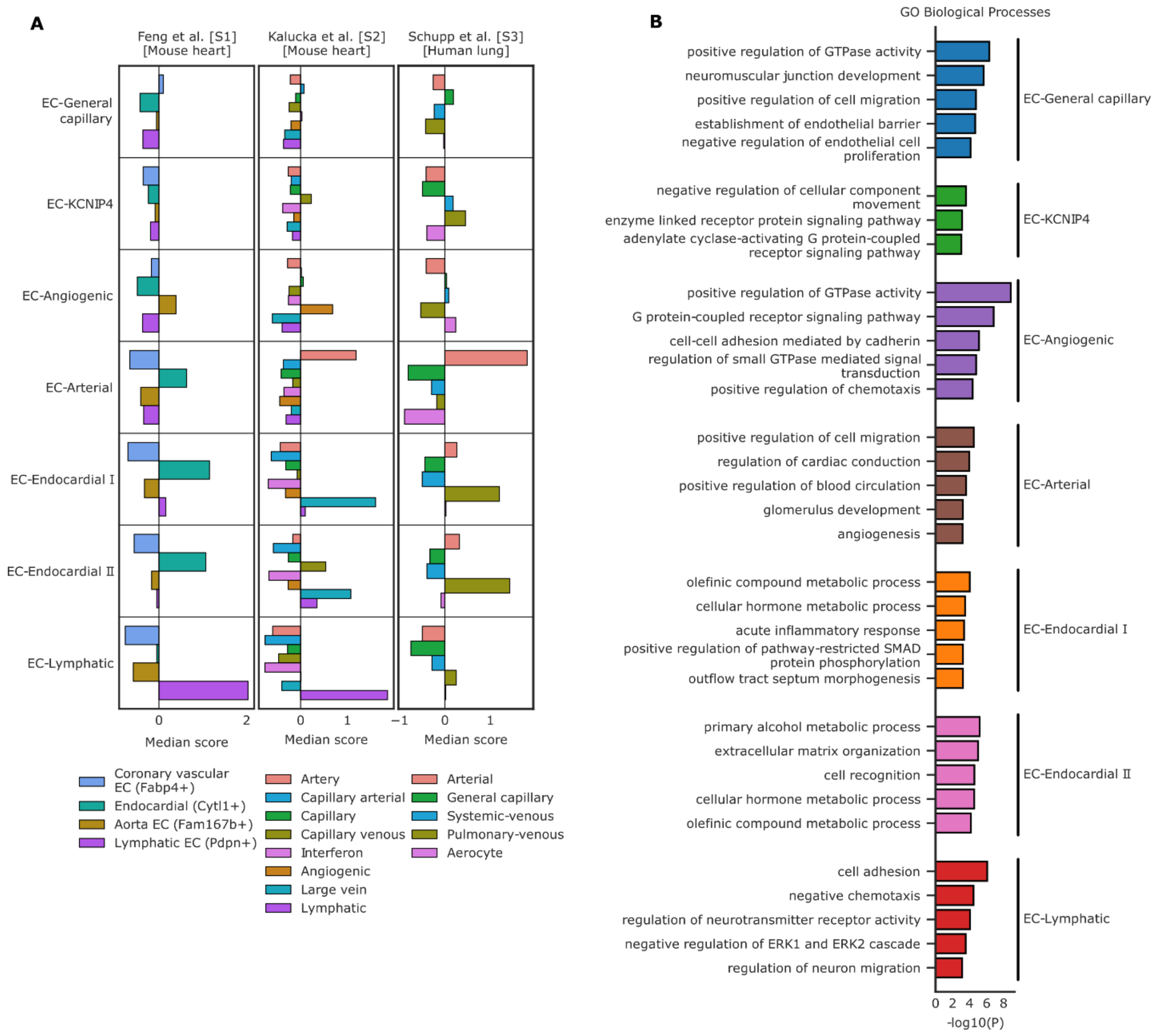

Supplemental Figure 4: Endothelial cell subcluster classification and pathway analysis, related to Figure 2. A. Assigned endothelial subtype classifications for six subclusters (EC-Angiogenic, EC-Arterial, EC-Endocardial I, EC-Endocardial II, EC-General Capillary, EC-Lymphatic) by comparing expression profiles to published data (see STAR Methods). **B**. Pathway analysis of significant marker genes of each endothelial cell subcluster using GO Biological Processes database. Only significant ontologies (P<0.001) are shown. EC, endothelial cell.

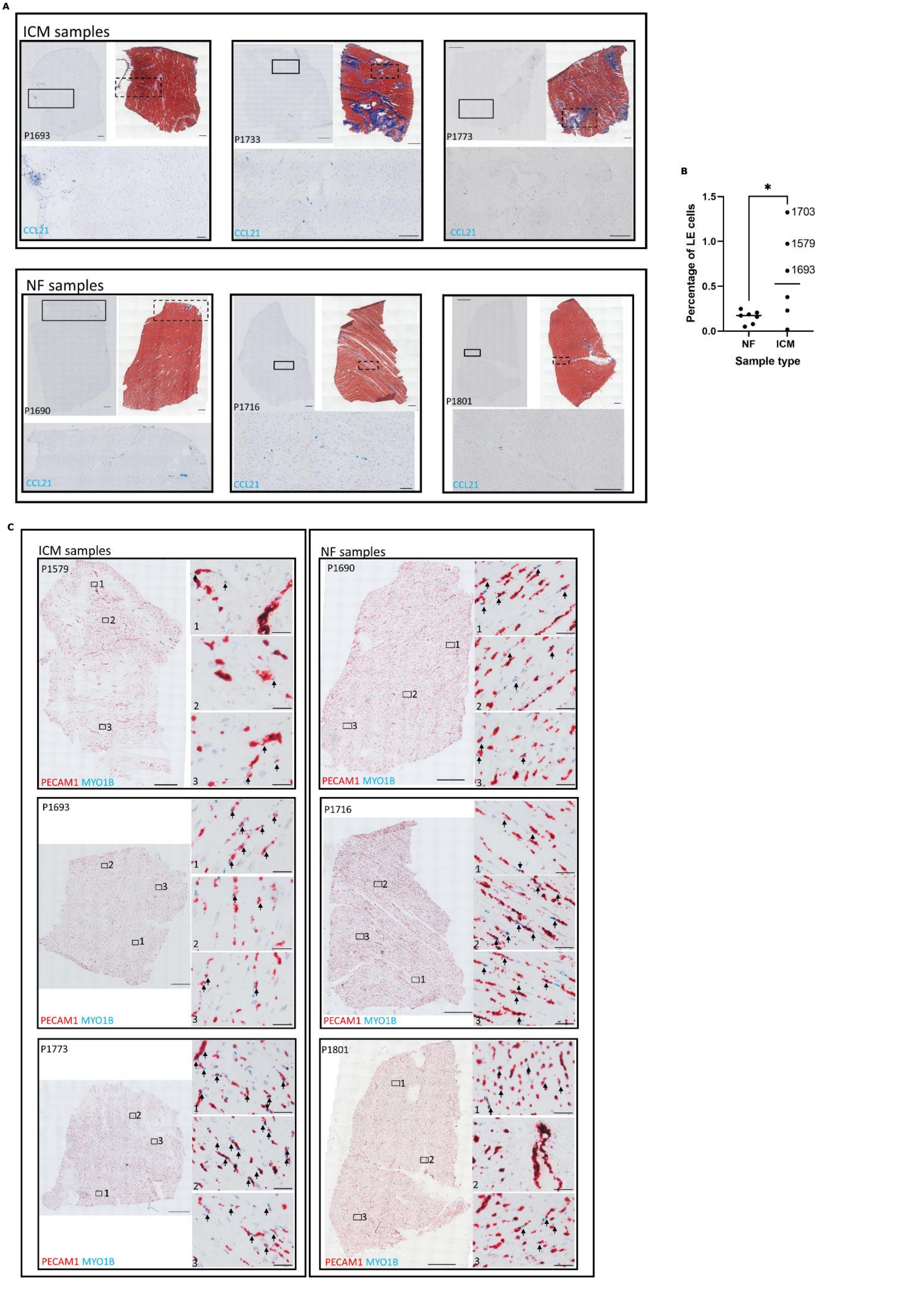

A
ICM samples
P1693
CCL21
P1733
CCL21
P1773
CCL21
NF samples
P1690
CCL21
P1716
CCL21
P1801
CCL21
B
*
Percentage of LE cells
1.5
1.0
0.5
0.0
1703
1579
1693
NF
ICM
Sample type
C
ICM samples
P1579
PECAM1 MYO1B
P1693
PECAM1 MYO1B
P1773
PECAM1 MYO1B
NF samples
P1690
PECAM1 MYO1B
P1716
PECAM1 MYO1B
P1801
PECAM1 MYO1B

Supplemental Figure 5: Example images of expanded in situ hybridization validation of EC-lymphatic subcluster and EC-angiongenic subcluster in three ischemic cardiomyopathy patients and three non-failing controls, related to Figure 2. **A** In situ hybridization with RNAscope showing localization of a canonical lymphatic endothelial cell marker, *CCL21* (blue) across sections from ischemic cardiomyopathy patients (P1693, P1733, P1773) and non-failing patients (P1690, P1716, P1801). Boxes on tile images show where the close-up images were taken, and dotted boxes show the corresponding region on the sequential trichrome stained sections. Nuclear localization is shown with hematoxylin (blue). **B** Quantification of RNAscope, percentage of *CCL21* positive cells over all nuclei, n = 7 (NF) and 6 (ICM), p = 0.0363. Line notes the median, *p<0.05, unpaired t-test. **C** In situ hybridization with RNAscope showing localization of a canonical endothelial marker *PECAM1* (red) and marker of EC-angiogenic subcluster *MYOB1* (blue) across sections from ischemic cardiomyopathy patients (P1579, P1693, P1773) and non-failing patients (P1690, P1716, P1801). Boxes on tile images show where the close-up images were taken, and arrows show double positive cells. Nuclear localization is shown with hematoxylin (blue). ICM, ischemic cardiomyopathy, NF, non-failing. Scale bars of tile represent 1 mm, and of close up of 250 um (A) or 50 um (C).

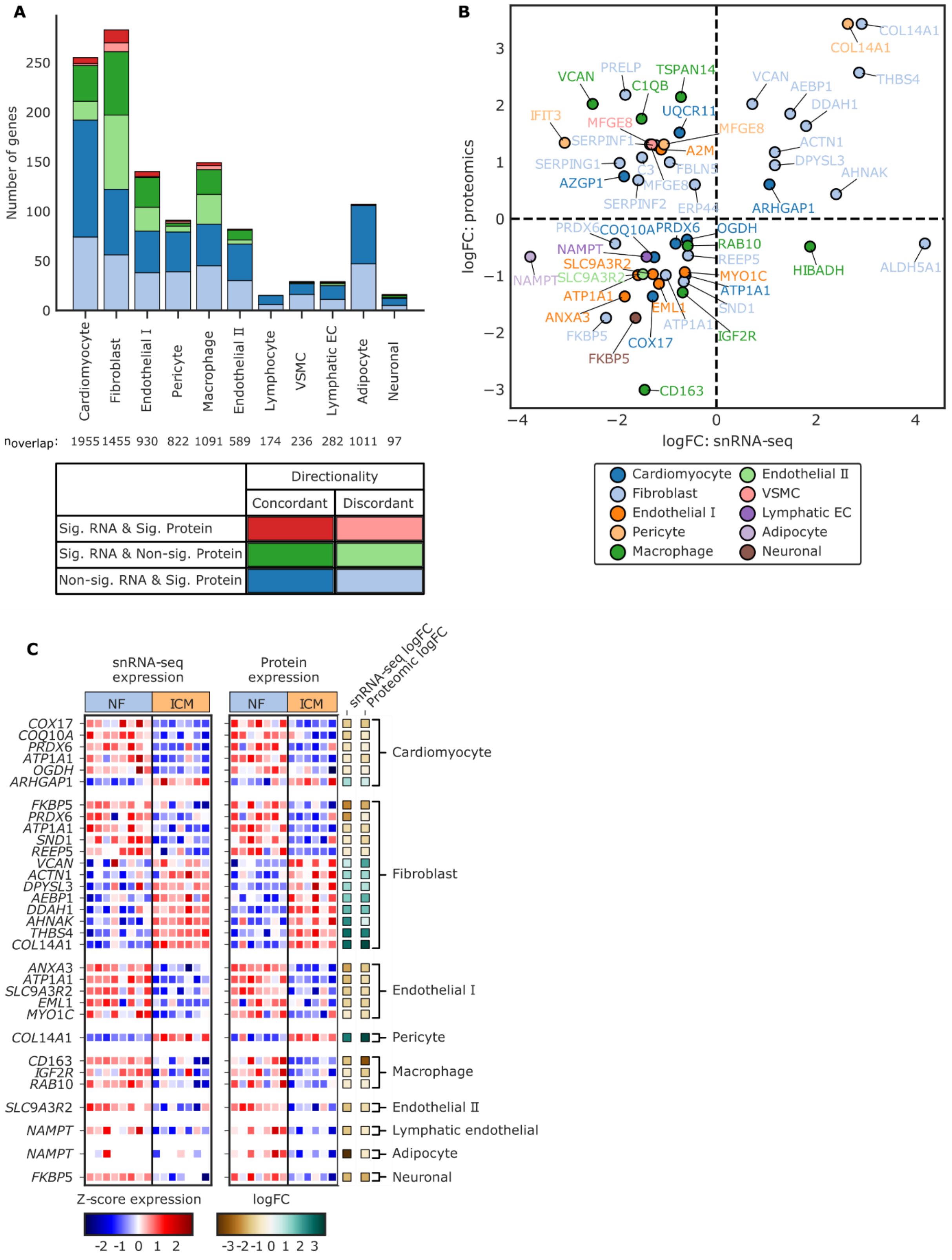
A
Number of genes
Cardiomyocyte
Fibroblast
Endothelial I
Pericyte
Macrophage
Endothelial II
Lymphocyte
VSMC
Lymphatic EC
Adipocyte
Neuronal
noverlap: 1955 1455 930 822 1091 589 174 236 282 1011 97
Directionality
Concordant
Discordant
Sig. RNA & Sig. Protein
Sig. RNA & Non-sig. Protein
Non-sig. RNA & Sig. Protein
B
logFC: proteomics
logFC: snRNA-seq
COL14A1
THBS4
VCAN
AEBP1
DDAH1
ACTN1
DPYSL3
AHNAK
ARHGAP1
PRELP
TSPAN14
C1QB
UQCR11
IFIT3
MFGE8
SERPINF1
A2M
SERPING1
C3
FBLN5
AZGP1
SERPINF2
ERP44
PRDX6
COQ10A
OGDH
RAB10
REEP5
NAMPT
SLC9A3R2
MYO1C
ATP1A1
ANXA3
EML1
SND1
FKBP5
COX17
IGF2R
CD163
HIBADH
ALDH5A1
Cardiomyocyte
Fibroblast
Endothelial I
Pericyte
Macrophage
Endothelial II
VSMC
Lymphatic EC
Adipocyte
Neuronal
C
snRNA-seq expression
Protein expression
NF
ICM
snRNA-seq logFC
Proteomic logFC
COX17
COQ10A
PRDX6
ATP1A1
OGDH
ARHGAP1
Cardiomyocyte
FKBP5
PRDX6
ATP1A1
SND1
REEP5
VCAN
ACTN1
DPYSL3
AEBP1
DDAH1
AHNAK
THBS4
COL14A1
Fibroblast
ANXA3
ATP1A1
SLC9A3R2
EML1
MYO1C
Endothelial I
COL14A1
Pericyte
CD163
IGF2R
RAB10
Macrophage
SLC9A3R2
Endothelial II
NAMPT
Lymphatic endothelial
NAMPT
Adipocyte
FKBP5
Neuronal
Z-score expression
logFC

Supplemental Figure 6: Correlation of snRNAseq data to proteomics data, related to Figure 1. A. Bar plot showing the number and proportion of overlap of significantly dysregulated genes from snRNAseq or proteomics in ICM. Bar colors indicate the statistical significance and consistency of the directionality between the proteomics and snRNAseq analyses. The total number of genes/proteins tested in the given cell type in the snRNAseq as well as in the bulk proteomics data is listed below each bar ($n_{overlap}$) **B**. Plot of correlation of differential RNA/protein expression results between ICM and NF based on snRNAseq and proteomic data for genes that were significantly differentially expressed in both datasets. The color of each dot represents the cell type of the snRNAseq differential expression test. **C.** Heatmap of effect size estimates for protein coding genes that were significantly differentially expressed between ischemic cardiomyopathy (ICM) and non-failing (NF) in snRNAseq data (FDR-adjusted P-value < 0.05, expressed in > 1% of nuclei from either group, low background probability) and proteomic data. Blue/red shading represents Z score expression, Tan/Green shading represents the overall log fold-change comparing ICM to NF (logFC). logFC, log fold-change; VSMC, vascular smooth muscle cell.

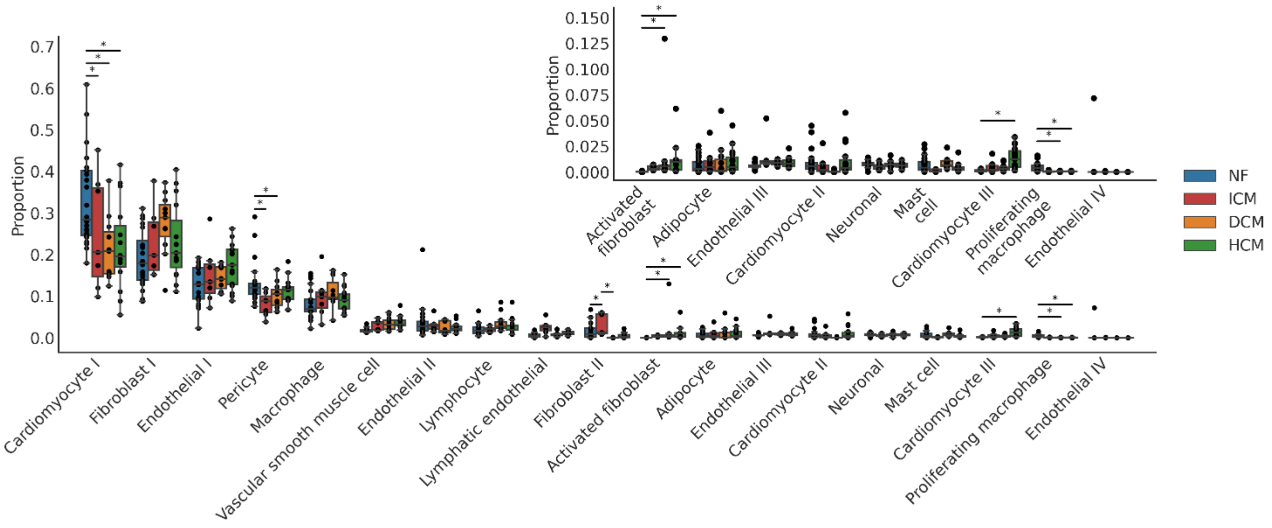

Supplemental Figure 7: Distribution of Cellular Composition in HCM/DCM/ICM combined dataset, related to Figure 5. Statistically credible changes are noted with a * (see STAR Methods). Box plots are represented as: Center line, median; Box limits, upper and lower quartiles; Whiskers, 1.5x interquartile range; Points, outliers.

Supplemental References

[S1] Feng, W., Chen, L., Nguyen, P.K., Wu, S.M., and Li, G. (2019). Single Cell Analysis of Endothelial Cells Identified Organ-Specific Molecular Signatures and Heart-Specific Cell Populations and Molecular Features. Front. Cardiovasc. Med. *6*, 165. 10.3389/fcvm.2019.00165.

[S2] Kalucka, J., de Rooij, L.P.M.H., Goveia, J., Rohlenova, K., Dumas, S.J., Meta, E., Conchinha, N.V., Taverna, F., Teuwen, L.-A., Veys, K., et al. (2020). Single-Cell Transcriptome Atlas of Murine Endothelial Cells. Cell *180*, 764-779.e20. 10.1016/j.cell.2020.01.015.

[S3] Schupp, J.C., Adams, T.S., Cosme, C., Raredon, M.S.B., Yuan, Y., Omote, N., Poli, S., Chioccioli, M., Rose, K.-A., Manning, E.P., et al. (2021). Integrated Single-Cell Atlas of Endothelial Cells of the Human Lung. Circulation *144*, 286–302. 10.1161/CIRCULATIONAHA.120.052318.

[S4] Zhang, Y., El-Sikhry, H, Chaudhary K. R., Batchu S. N., Shayeganpour, A., Jukar, T. B., Bradbury, J. A., Graves, J. P., DeGraff, L. M., Myers, P., et al (2009). Overexpression of CYP2J2 provides protection against doxorubicin-induced cardiotoxicity. Am J. Physiol Heart Circ Physiol. *297*: H37-46. 10.1152/ajpheart.00983.2008

[S5] Wang, B., Zeng, H., Wen, Z., Chen, C., Wang, D. W. (2016). CYP2J2 and its metabolites (epoxyeicosatreinoic acids) attenuates cardiac hypertrophy by activating AMPKa2 and enhancing nuclear translocation of Akt1. Aging Cell. *15*:940-952. 10.1111/acel.12507

[S6] Aliwarga, T., Evangelista, E. A., Sotoodehnia, N., Lemaitre, R. N., Totah, R. A. (2018) Regulation of CYP2J2 and EET levels in Cardiac Disease and Diabetes. Int. J. Mol. Sci *29* 1916 10.3390/ijms19071916

[S7] Valencia, R., Bassiouni, W., Darwesh, A. M., Bapuji, R., Seubert, J. M. (2022) Cardiomyocyte-specific CYP2J2 and its therapeutic implications. Expert Opin Drug Metab Toxicol. *18*: 423-439 10.1080/17425255.2022.2114344

[S8] Gil-Cayuela, C., Rosello-Lleti, E., Ortega, A., Tarazon, E., Trivino, J. C., Martinez-Dolz, L., Gonzalez-Juanatey, J. R., Lago, F., Portoles, M., Rivera M. (2016) New Altered Non-Fibrillar Collagens in Human Dilated Cardiomyopathy: Role in the Remodeling Process. PLoS One *11*: e0168130 10.1371/journal.pone.0168130

[S9] Galindo, C. L., Kasasbeh, E., Murphey, A., Ryzhov, S., Lenihan, S., Ahmad, F. A., Williams, P., Nunnally, A., Adcock, J., Song, Y., et al. (2014) Anti-remodeling and anti-fibrotic effects of the neuregulin-1b glial growth factor 2 in a large animal model of heart failure. JAHA. *3*: e000773 10.1161/JAHA.113.000773

[S10] Holm, H., Gudbjartsson, D. F., Sulem P., Masson, G., Helgadottir, H. T., Zanon, C, Magnusson, O. H., Helgason, A., Saemundsdottir, J., Gylfason, A., et al. (2011). A rare variant in MYH6 is associated with high risk of sick sinus syndrome. Nat. Genet. *43*: 316-320 10.1038/ng.781

[S11] Theis, J. L., Zimmermann, M. T., Evans, J. M., Eckloff, B. W., Wieben, E. D., Qureshi, M. Y., O'Leary, P. W., Olson T. M. (2015). Recessive MYH6 mutations in hypoplastic left heart with reduced ejection fraction. Circ Genetics *8:* 564-571 10.1161/CIRCGENETICS.115.001070

[S12] Granados, J. T., Ghosh, T. K., Pople, M., Bu'Lock, F., Thornborough, C., Eason, J., Kirk, E. P., Fatkin, D., Feneley, M. P., Harvey, R. P., et al. (2010). a-Cardiac myosin heavy chain (MYH6) mutations affecting myofibril formation are associated with congenital heart defects. Human Molecular Genetics, *19*: 4007-4016 10.1093/hmg/ddq315

[S13] Jiang, J., Wakimoto, H., Seidman, J. G., Seidman, C. E. 2013. Allele-specific silencing of mutant Myh6 transcripts in mice suppresses hypertrophic cardiomyopathy. Science *342*:111-114 10.1126/science.1236921

[S14] Justo, T., Martiniuc, A., Dhoot, G. K. (2021) Modulation of cell signalling and sulfation in cardiovascular development and disease. Sci Rep. *11*: 22424 10.1038/s41598-021-01629-0

[S15] Korf-Klingebiel, M., Reboll, M. R., Grote, K., Schleiner, H., Wang, Y., Wu, X., Klede, S., Mikhed, Y., Bauersachs, J., Klintschar, M., et al. (2019) Heparan Sulfate-Editing Extracellular Sulfatases Enhance VEGF Bioavailability for Ischemic Heart Repair. Circ. Res. *11*: 787-801 10.1161/CIRCRESAHA.119.315023

[S16] Yue, X., Li, X., Nguyen H. T., Chin, D. R., Sullivan D. E, Lasky, J. A. (2008) Transforming growth factor-beta1 induces heparan sulfate 6-O-endosulfatase 1 expression in vitro and in vivo. J. Biol Chem *18*: 20397-407 10.1074/jbc.M802850200

[S17] Gerarduzzi, C., Kumar, R. K., Trivedi, P., Ajay, A. K., Iyer, A., Boswell, S., Hutchinson J. N., Waikar, S. S., Vaidya, V. S. (2017) Silencing SMOC2 ameliorates kidney fibrosis by inhibiting fibroblast to myofibroblast transformation. JCI Insight. *2*: e90299 10.1172/jci.insight.90299

[S18] Luo, L., Wang, C-C., Song, X-P., Wang, H-M., Zhou, H., Sun, Y., Wang, X-K., Hou, S., Pei, F-Y. (2018) Suppression of SMOC2 reduces bleomycin (BLM)-induced pulmonary fibrosis by inhibition of TGF-b1/SMADs pathway. *105*: 841-847 10.1016/j.biopha.2018.03.058

[S19] Yuting, Y., Lifeng, F., Qiwei, H. (2019) Secreted modular calcium-binding protein 2 promotes high fat diet (HFD)-induced hepatic steatosis through enhancing lipid deposition fibrosis and inflammation via targeting TGF-b1. Biochem Biophys Res Commun. *509*: 48-55 10.1016/j.bbrc.2018.12.006

[S20] Bergmeier, V., Etich, J., Pitzler, L., Frie, C., Koch, M., Fischer, M., Rappl, G., Abken, H., Tomasek, J. J., Brachvogel, B. (2018) Identification of a myofibroblast-specific expression signature in skin wounds. Matrix Biol. *65*: 59-74 10.1016/j.matbio.2017.07.005

[S21] Tao, G., Levay, A. K., Peacock, J. D., Huk, D., J., Both, S. N., Purcell, N. H., Pinto, J. R., Galantowicz, M. L, Koch, M., Lucchesi, P. A., et al. (2012) Collagen XIV is important for growth and structural integrity of the myocardium. J. Mol Cell Cardiol. *53*: 626-38 10.1016/j.yjmcc.2012.08.002

[S22] Ma, Z., Wang, X., LV, Q., Gong, Y., Xia, M., Zhuang, L., Lu, X., Yang, Y., Zhang, W., Fu, G., et al. (2021) Identification of underlying hub genes associated with hypertrophic cardiomyopathy by integrated bioinformatics analysis. Pharmogenomics Pers Med. *14*: 823-837 10.2147/PGPM.S314880

[S23] Xie, T., Wang, Y., Deng, N., Huang, G., Taghavifar, F., Geng, Y., Liu, N., Kulur, V., Yao, C., Chen, P., et al. (2018) Single-cell deconvolution of fibroblast heterogeneity in mouse pulmonary fibrosis. Cell Rep. *22*: 3625-3640 10.1016/j.yjmcc.2012.08.002

[S24] Komatsu, M., Kanda, T., Urai, H., Kurokochi, A., Kitahama, R., Shigaki, S., Ono, T., Yukioka, H., Hasegawa, K., Tokuyama, H., et al. (2018) NNMT activation can contribute to the development of fatty liver disease by modulating the NAD+ metabolism. Sci Rep. *8*:8637 10.1038/s41598-018-26882-8

[S25] Takahashi, R., Kanda, T., Komatsu, M., Itoh, T., Minakuchi, H., Urai, H., Kuroita, T., Shigaki, S., Tsukamoto, T., Higuchi, N., et al. (2022) The significance of NAD+ metabolites and nicotinamide N-methyltransferase in chronic kidney disease. Sci Rep *12*: 6398 10.1038/s41598-022-10476-6

[S26] Samuchiwal, S. K., Balestrieri, B. (2018) Harmful and protective roles of group V Phospholipase A2: current perspectives and future directions. Biochim Biophys Acta Mol Cell Biol Lipids. *1864*: 819-826 10.1016/j.bbalip.2018.10.001

[S27] Zhang, Y., Fu, C., Zhao, S., Jiang, H., Li, W., Liu X. (2022) PRELP promotes myocardial fibrosis and ventricular remodelling after acute myocardial infarction by the wnt/b-catenin signalling pathway. Cardiovasc J Afr. *33*:1-6 10.5830/CVJA-2022-001

[S28] Chacon-Solano, E., Leon, C., Carretero, M., Garcia, M., Sanchez-Dominguez, R., Quero, F., Mendez-Jimenez, E., Bonafont, J., Ruiz-Mezcua, B., Escamez, M. J., et al. (2022) Mechanistic interrogation of mutation-independent disease modulators of RDEB identifies the small leucine-rich proteoglycan PRELP as a TGF-b antagonist and inhibitor of fibrosis. *111*:189-206 10.1016/j.matbio.2022.06.007

[S29] Du, Z., Lin, Z., Wang, Z., Liu, D., Tian, D., Xia, L. (2020) SPOCK1 overexpression induced by platelet-derived growth factor-BB promotes hepatic stellate cell activation and liver fibrosis through the interin a5b1/PI3K/Akt siganling pathway. Lab Invest. *100:* 1042-1056 10.1038/s41374-020-0425-4

[S30] Singh, P., Rai, A., Dohare, R., Arora, S., Ali, S., Parveen, S., Syed, M. A. (2020) Network-based identification of signature genes KLF6 and SPOCK1 associated with oral submucous fibrosis. Mol Clin Oncol. *12*:299-310 10.3892/mco.2020.1991

[S31] Kolur, V., Vastrad, B., Bastrad, C., Kotturshetti, S., Tengli, A. (2021) Identification of candidate biomarkers and therapeutic agents for heart failure by bioinformatics analysis. BMC Cardiovasc Disord. *21*: 329 10.1186/s12872-021-02146-8

[S32] di Salvo, T., G., Yang, K-C., Brittain, E., Absi, T., Maltais, S., Hemnes, A. (2015) Right ventricular myocardial biomarkers in human heart failure. J. Card. Fail. *21*:398-411 10.1016/j.cardfail.2015.02.005

[S33] Liu, B., Xiang, L., Ji, J., Liu, W., Chen, Y., Xia, M., Liu, Y., Liu, W., Zhu, P., Jin, Y., et al. (2021) SPARCL1 promotes nonalcoholic steatohepatitis progression in mice through upregulation of CCL2. J. Clin. Invest. *131*:e144801 10.1172/JCI144801

[S34] Ye, H., Wang, W-G., Cao, J., Hu, X-C. (2017) SPARCL1 suppresses cell migration and invasion in renal cell carcinoma. Mol Med Rep. *16*:7784-7790 10.3892/mmr.2017.7535

[S35] Salmasi, M., Y., Morris-Reosendahl, D., Jarral, O. A., Rosendahl, U., Asimakopoulos, G., Raja, S., Aragon-Martin, J. A., Child, A., Pepper, J., et al. London Aortic Mechanobiology Working Group. (2022) Determining the genetic contribution in patients with non-syndromic ascending thoracic aortic anuerysms: Correlation with findings from computational pathology. Int. J. Cardiol. *1*:1-9 10.1016/j.ijcard.2022.07.010

[S36] Zhang, R., Xu, X., Chen, X., Hao, C., Ji, Z., Zuo, P., Yang, M., Ma, Genshan, M., Li, Y. (2022) Upregulation of key genes ELN and TGFB3 were association with the severity of cardiac hypertrophy. BMC Genomics. *14*:592 10.1186/s12864-022-08778-0

[S37] de Brouwer, B., Drent, M., van den Ouweland, J. M. W., Wignen, P. A., van Moorsel, C. H. M., Bekers, O., Crutters, J. C., White, E. S., Janssen, R. (2018) Increased circulating desmosine and age-dependent elastinolysis in idiopathic pulmonary fibrosis. Respir. Res. *19*:45 10.1186/s12931-018-0747-6
