# Supplementary material for: Single-nucleus RNA sequencing in ischemic cardiomyopathy reveals common transcriptional profile underlying end-stage heart failure
Source: Cell Rep. Author manuscript; Available in PMC 2023 Oct 23. (PMC10423750; doi:10.1016/j.celrep.2023.112086)
Supplement: 5 [file NIHMS1878882-supplement-5.pdf]

Cell Reports

Article

Single-nucleus RNA sequencing in ischemic cardiomyopathy reveals common transcriptional profile underlying end-stage heart failure

Graphical abstract

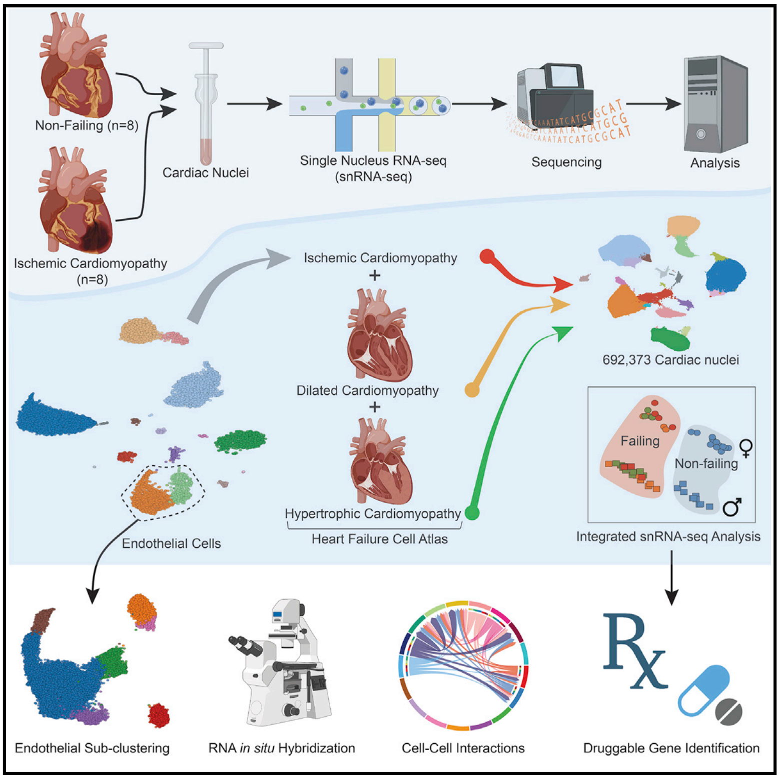

Highlights

- Cellular expression and composition are altered in ischemic cardiomyopathy
- LAMININ signaling from endothelial cells to other cell types increases in disease
- Cell-type transcriptional profiles converge across multiple cardiomyopathy subtypes
- Potential druggable targets identified for end-stage heart failure

Authors

Bridget Simonson, Mark Chaffin, Matthew C. Hill, ..., Kenneth B. Margulies, Carla A. Klattenhoff, Patrick T. Ellinor

Correspondence

ellinor@mgh.harvard.edu

In brief

Simonson et al. compare non-failing and ischemic cardiomyopathy human left ventricles using snRNA-seq and identify an increase in lymphatic and angiogenic endothelial cells in disease. Transcriptionally, ischemic cardiomyopathy is similar to other cardiomyopathies. Gene targets from this shared expression profile could be explored for therapeutic benefit in end-stage heart failure.

Simonson et al., 2023, Cell Reports *42*, 112086
February 28, 2023 © 2023 The Authors.
https://doi.org/10.1016/j.celrep.2023.112086

CellPress

Cell Reports

CellPress
OPEN ACCESS

Article

Single-nucleus RNA sequencing in ischemic cardiomyopathy reveals common transcriptional profile underlying end-stage heart failure

Bridget Simonson,[1,2,7] Mark Chaffin,[1,2,7] Matthew C. Hill,[1,2,3] Ondine Atwa,[1,2] Yasmine Guedira,[1,2] Harshit Bhasin,[1,2] Amelia W. Hall,[1,4] Sikander Hayat,[5] Simon Baumgart,[5] Kenneth C. Bedi, Jr.,[6] Kenneth B. Margulies,[6] Carla A. Klattenhoff,[5] and Patrick T. Ellinor[1,2,3,8,*]

[1]Precision Cardiology Laboratory, The Broad Institute of MIT and Harvard, Cambridge, MA 02142, USA
[2]Cardiovascular Disease Initiative, The Broad Institute of MIT and Harvard, Cambridge, MA 02142, USA
[3]Cardiovascular Research Center, Massachusetts General Hospital, Boston, MA 02114, USA
[4]Gene Regulation Observatory, The Broad Institute of MIT and Harvard, Cambridge, MA 02142, USA
[5]Precision Cardiology Laboratory, Bayer US, LLC, Cambridge, MA 02142, USA
[6]Penn Cardiovascular Institute, University of Pennsylvania, Philadelphia, PA 19104, USA
[7]These authors contributed equally
[8]Lead contact
*Correspondence: ellinor@mgh.harvard.edu
https://doi.org/10.1016/j.celrep.2023.112086

SUMMARY

Ischemic cardiomyopathy (ICM) is the leading cause of heart failure worldwide, yet the cellular and molecular signature of this disease is largely unclear. Using single-nucleus RNA sequencing (snRNA-seq) and integrated computational analyses, we profile the transcriptomes of over 99,000 human cardiac nuclei from the non-infarct region of the left ventricle of 7 ICM transplant recipients and 8 non-failing (NF) controls. We find the cellular composition of the ischemic heart is significantly altered, with decreased cardiomyocytes and increased proportions of lymphatic, angiogenic, and arterial endothelial cells in patients with ICM. We show that there is increased LAMININ signaling from endothelial cells to other cell types in ICM compared with NF. Finally, we find that the transcriptional changes that occur in ICM are similar to those in hypertrophic and dilated cardiomyopathies and that the mining of these combined datasets can identify druggable genes that could be used to target end-stage heart failure.

INTRODUCTION

Ischemic cardiomyopathy (ICM) is the most prevalent type of heart disease, affecting 126 million people worldwide and causing 9 million deaths in 2017.[1] ICM is most commonly caused by atherosclerotic coronary artery disease inhibiting blood flow to the heart. This leads to decreased oxygen in the myocardium causing cardiomyocyte death and cardiac remodeling via formation of a fibrotic scar. Over time the lack of oxygen and formation of the scar leads to a loss of contractile function, cardiac hypertrophy, dilation, and heart failure.[2] Current therapeutics for ICM include medical interventions such as ACE inhibitors and beta blockers, and surgical revascularization therapy; however, for patients progressing to heart failure the long-term prognosis remains poor.[3,4]

ICM has been studied in detail in animal models where disease progression can be monitored closely over time.[4,5] These efforts have identified specific cell types, genes, and pathways that play crucial roles in ICM. One important cardiac cell type responsible for responding to ischemic injury is vascular endothelial cells. Endothelial cells play an important role throughout the remodeling of the heart after ischemia.[6] During the initial immune response, they contribute to white blood cell infiltration to the infarcted region, and they release cytokines and chemokines, which contribute to fibroblast activation and cardiomyocyte health.[7] The formation of functional new vessels from the pre-existing vascular network of endothelial cells, known as angiogenesis, is essential to supply damaged tissue with oxygen and nutrients.[8] During post-ischemic cardiac remodeling lymphangiogenesis also occurs, and lymphatic endothelial cells play a role in clearing fluid buildup, debris, and dead cells from the infarct.[9] Both angiogenesis and lymphangiogenesis are processes that have been targeted as having therapeutic potential for ICM, with mixed results shown in animal studies and in clinical trials.[10,11]

As developing therapeutic targets for such a dynamic cardiovascular disease is challenging, there is a need for greater cellular and transcriptional information from human ICM patients. Advances in single-cell and single-nucleus RNA sequencing (snRNA-seq) have allowed a greater understanding of the underlying transcriptional profile of the healthy[12,13] and diseased[14,15] human heart. Here, we utilize snRNA-seq to profile the left ventricle (LV) of transplant recipients with ICM and non-failing (NF) controls.

© 2023 The Authors.
This is an open access article under the CC BY license (http://creativecommons.org/licenses/by/4.0/).

There were three aims for this study. First, to broadly characterize the cellular composition and transcriptional changes seen in the non-infarct region of the LV of ICM patients. Second, to assess the status of the healthy and ischemic vasculature. And finally, to compare the transcriptional landscape of ICM with other forms of end-stage heart failure, including hypertrophic cardiomyopathy (HCM) and dilated cardiomyopathy (DCM). Together, our results show that there are large changes in both cellular composition and transcription in ICM compared with NF patients. These changes are similar to those seen in HCM and DCM, suggesting that these three types of heart failure share a similar end-stage transcriptional profile. Further investigation into this shared transcriptional profile may lead to identification of new potential therapeutic targets for end-stage heart failure.

RESULTS

In this study, we performed snRNA-seq on samples from the non-infarcted region of the LV of human patients with long-term ICM (n = 8) and NF controls (n = 8). After strict quality control, 1 ICM sample was removed leaving a final dataset of 8 control and 7 ICM human samples (Table 1). After removal of low-quality nuclei, 99,684 nuclei (39,339 ICM; 60,345 NF) across 16 clusters remained (Figures 1A and S1; Table S1). Cell-type labels were assigned to clusters based on the expression of canonical marker genes and the enrichment of gene ontology-based biological processes (Tables S2 and S3). As shown previously, the most abundant cell types were cardiomyocytes (31.6%), fibroblasts (22.6%), and endothelial cells (20.5%)[13] (Figure 1B). Compared with NF controls, we observed significant decreases in cardiomyocytes and endothelial II populations in ICM, with a non-significant increase in fibroblasts (Figure 1C). Masson trichrome staining of tissue sections from these samples showed a large range of fibrosis in the ICM samples, which, when quantified, corresponded with the proportion of fibroblasts from the sample-matched snRNA-seq data (Figures S2A–S2C). We next performed an exploratory differential expression test to identify transcriptional changes in fibroblasts correlating with the degree of fibrosis in ICM samples. The strongest changes were observed for *BMP6*, *KCNMA1*, *CLSTN2*, *TEX41*, *MALRD1*, and *ACSM3* (Figure S2D). Interestingly, *BMP6* is a member of the TGF-β superfamily,[16] and *KCNMA1* is a subunit of the BK channel which regulates the degree of α-SMA expression after TGF-β stimulation in pulmonary fibroblasts.[17]

Changes in gene expression and pathways in ICM compared with NF

To identify transcriptional differences between ICM and NF, we carried out differential gene expression testing on the main cell clusters by aggregating nuclei across samples into a pseudo-bulk representation (see STAR Methods). We observed the highest number of differentially expressed genes (DEGs) in fibroblasts (n = 1,525) followed by cardiomyocytes (n = 513), endothelial I cells (n = 457), and macrophages (n = 415) (Figures 1D and S3A; Table S4).

The top up- and downregulated genes for each cell type are shown in Figure S3A. Interesting DEGs in fibroblasts include: *NTM*, which has been identified as a novel biomarker for heart failure,[18] and *ANO1*, which plays an important role in fibrosis in a rat model of myocardial infarction.[19] In cardiomyocytes, *PLCE1* has been shown to play a role in cardiac hypertrophy via its interaction with the scaffolding protein mAKAP6.[20] *AZGP1* is a secreted protein that has been shown to have anti-fibrotic effects in the kidney and the heart[21] and *CALCOCO2*[22] is a key protein in autophagy and its expression is associated

Table 1. Clinical characteristics of study participants

	Cases	Controls
Total (n)	7	8
Males (%)	2 (33)	4 (50)
Age (mean ± SD)	58 (6.2)	54.63 (7.6)
BMI (mean ± SD)	29.9 (4.5)	30.2 (8.4)
History of hypertension n (%)	5 (71)	2 (25)
History of ICM (%)	7 (100)	0
History of diabetes (%)	3 (43)	0
History of pacemaker device (%)	7 (100)	0
History of CABG (%)	3 (43)	0
Creatinine (mean ± SD)	1.03 (0.25)	1.9 (1.58)
Initial infarct location n (%)		
Anterior wall	2 (29)	0
Anteroseptal	1 (14)	0
Anterior apical wall	1 (14)	0
Diffuse	2 (29)	0
Time between first infarct and transplant (years, range)	8.32 (0.4–25)	0
Medications n (%)		
Anticoagulants	4 (57)	1 (12.5)
Digoxin	2 (29)	0
Amiodarone	1 (14)	0
ACE inhibitor	6 (87)	1 (12.5)
Beta blocker	7 (100)	0
Angiotensin receptor blockers	1 (14)	0
Calcium channel blockers	2 (29)	0
Nitrate	2 (29)	0
Diuretics	7 (100)	0
Lipid lowering	7 (100)	0
Antiplatelet	4 (57)	0
Acetylsalicylic acid (aspirin)	5 (71)	1 (12.5)
Echocardiography		
LVEF % (mean ± SD)	23 (17.4)	62.44 (4.8)****
LVESD cm (mean ± SD)	4.85 (1.7)	2.56 (0.15)*
LVEDD cm (mean ± SD)	5.704 (1.5)	3.960 (0.31)*
LVM g (mean ± SD)	267 (65.4)	197 (40.3)*
LVMI (Male)	119 (25.41)	112.8 (12.16)
LVMI (Female)	149.8 (44.52)	82.77 (4.58)*

ACE, angiotensin-converting enzyme; BMI, body mass index; CABG, coronary artery bypass graft; LVEF, left ventricular ejection fraction; LVESD, left ventricular end systolic dimension; LVEDD, left ventricular end diastolic dimension; LVM, left ventricular mass; LVMI, left ventricular mass index.
*$p < 0.05$, **$p < 0.01$, ****$p < 0.0001$.

CellPress
OPEN ACCESS

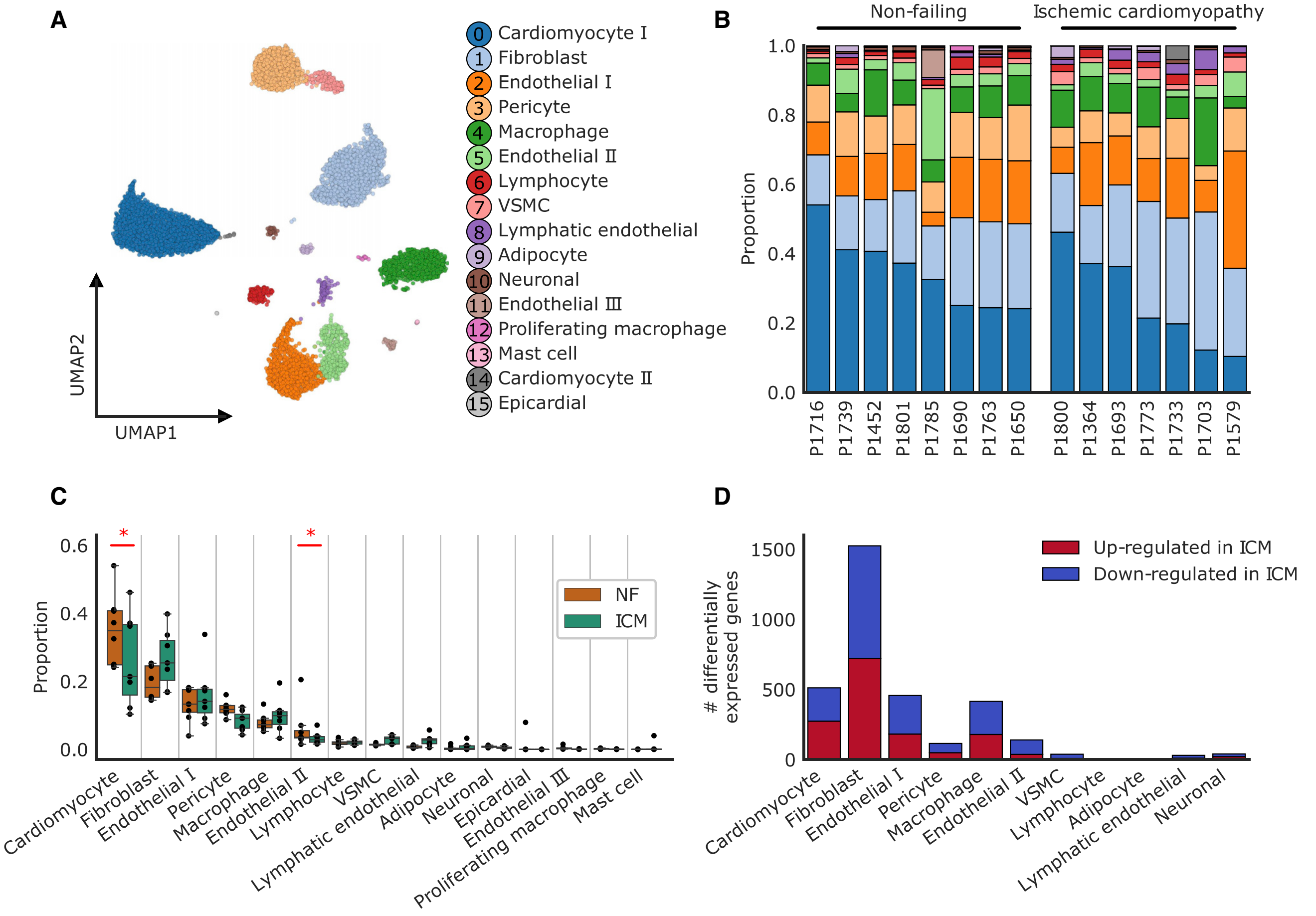

Figure 1. Cellular composition of non-failing and failing ischemic cardiomyopathy

(A) UMAP representation of 99,684 nuclei from 8 NF samples and 7 ICM samples showing 16 cell clusters identified with unsupervised Leiden clustering. VSMC, vascular smooth muscle cell.

(B) Cell-type composition of each individual sample, with colors representing cell types labeled in (A).

(C) Changes in composition observed between NF and ICM samples. Statistically credible changes are noted with an * (see STAR Methods). Boxplots are represented as: center line, median; box limits, upper and lower quartiles; whiskers, 1.5× interquartile range; points, outliers.

(D) Number of significantly differentially expressed genes in each cell type in ICM compared with NF. Upregulated genes are shown in red and downregulated genes are shown in blue.

See also Figures S1–S3 and Tables S1, S2, S3, S4, and S5.

with cardiac dysfunction in DCM. Finally, in the macrophages, *ABCA9* is an important gene in monocyte to macrophage differentiation[23] and *CD163L1* expression in macrophages is associated with tissue resident anti-inflammatory macrophages in cancer.[24]

To determine if these transcriptional changes in ICM were enriched in specific pathways, we performed pathway analysis using the gene ontology biological processes[25] and Reactome pathway databases.[26] In ICM, differentially regulated pathways were identified in cardiomyocytes, fibroblasts, endothelial I and II cells, macrophages, and pericytes (Figures S3B–S3E; Table S5). In fibroblasts, there was increased expression of genes involved in the extracellular matrix organization and collagen formation pathways. In cardiomyocytes, there was increased expression of genes involved in immune effector processes, and decreased expression of genes involved in metabolism. Notably, in endothelial cells we observed increased expression of genes involved in cell proliferation and angiogenesis, including activation of vascular endothelial growth factor (VEGF) signaling pathways, and regulation of cellular response to growth factor stimulus.

Endothelial cell diversity in ICM

Endothelial cells play a crucial role in the remodeling of the heart post myocardial infarction.[27] Due to the enrichment of pathways involved in endothelial cell proliferation and angiogenesis in late-stage ICM, we further investigated the role of vascular cells in ICM. Specifically, we sought to characterize the cellular diversity of endothelial cells and look for subpopulations enriched in ICM. To do this, we performed subclustering on the combination of the endothelial I, endothelial II, endothelial III, and lymphatic endothelial populations, and from this we identified seven

distinct subclusters (Figures 2A and 2B; Tables S6 and S7). We assigned endothelial subtype classifications for six subclusters (EC-angiogenic, EC-arterial, EC-endocardial I, EC-endocardial II, EC-general capillary, EC-lymphatic) by comparing their expression profiles with published data[28–30] and assessing enriched ontologies (Figures S4A and S4B). One additional subcluster, EC-*KCNIP4*, could not be assigned to known endothelial subtype classifications.

We observed distinct shifts in endothelial cell composition in ICM compared with NF samples, including an increase in EC-lymphatic, EC-angiogenic, and EC-arterial cells (Figures 2C and 2D). Increases in EC-lymphatic cells were consistent across most ICM samples, whereas increases in EC-arterial cells were mostly found in one ICM sample (P1579). Increases in EC-angiogenic cells were mostly found in ICM samples P1579 and P1364. We also saw a non-significant increase in EC-endocardial I and II subclusters in NF compared with ICM, which was mostly derived from sample P1785 (Figure 2C). To validate the shifts observed in EC-lymphatic and EC-angiogenic cells we carried out *in situ* hybridization. Using the EC-lymphatic marker gene *CCL21* (logFC = 5.2, $p = 4.0 \times 10^{-22}$) we observed increased numbers of EC-lymphatic cells in the ICM samples that were clustered in fibrotic regions in the tissue (Figures 2E, S5A, and S5B). To validate the EC-angiogenic cells, we co-stained with the endothelial cell marker *PECAM1* and EC-angiogenic cell-specific marker *MYO1B* (logFC = 2.15, $p = 1.7 \times 10^{-14}$). The double-positive EC-angiogenic cells were largely found in the capillaries throughout the tissue (Figures 2F and S5C).

To further understand the differences in endothelial cells in ICM compared with NF we examined the top differentially regulated genes from each subcluster (Figure 3; Table S8). Some genes were differentially expressed between ICM and NF patients in most subclusters of endothelial cells. For example, *HIF3A*, a hypoxia-induced transcription factor, was significantly decreased in ICM patients compared with NF patients in EC-angiogenic, EC-*KCNIP4*, EC-arterial, EC-general capillary, and EC-endocardial I cells. *HIF3A* is a negative regulator of HIF1a/2a response to hypoxia.[31] Knockout of a variant of *HIF3A* that is expressed in neonatal and embryonic cells in mice leads to the enlargement of the right ventricle of mice, and increased endothelin-1 expression in lung endothelial cells.[32] We also saw decreased expression of *SYNE1* in ICM patients compared with NF patients in EC-general capillary, EC-*KCNIP4*, and EC-angiogenic cells. *SYNE1* encodes the nesprin 1 protein, which plays an important role in regulating cell shape, angiogenic loop formation, and migration of endothelial cells.[33]

We next examined DEGs in the three subclusters with increased prevalence in ICM compared with NF: EC-arterial, EC-angiogenic, and EC-lymphatic cells (Figure 3). Comparing ICM with NF patients in the EC-arterial population, there was an increase in *INSR* (insulin receptor), an important gene in angiogenesis.[34] There was also an increase in *XAF1* and decrease in *GSN*, two genes involved in regulation of apoptosis.[35,36] Finally, *SMAD6*, an anti-inflammatory and a negative regulator of BMP and TGF-β signaling, was decreased in ICM patients compared with NF patients.[37]

Comparing ICM with NF patients in the EC-angiogenic population, we saw an increase in *INSR* and a decrease in *PIK3C2A*, a protein previously shown to be decreased in the plasma of patients after an acute myocardial infarction.[38] *AFAP1L1*, which is involved in actin stress fiber formation,[39] was increased in both the EC-angiogenic and EC-general capillary populations (Figure 3).

Within the EC-lymphatic subcluster, *MMRN1*, was decreased in ICM compared with NF. *MMRN1* is an ECM protein that is a marker gene for lymphatic endothelial cells and binds to collagens I, II, and III, which leads to enhanced activated platelet adhesion and thrombus formation after injury.[40] EC-lymphatic and EC-endocardial I nuclei also had high expression of the tumor suppressor gene *PKHD1L1* in NF samples, which was strongly decreased in ICM. *PHKD1L1* plays a role in inhibiting proliferation and cell invasion of cancer cells[41] (Figure 3).

We were also interested in understanding if there were any changes in cell-cell communication between endothelial cells and the other cell types in the heart. To do this, we performed an analysis using CellChat.[42] We find an increase in overall communication involving endothelial cells in ICM compared with NF (Figure 4A), which is particularly pronounced in endocardial and angiogenic endothelial cells (Figure 4B). When considering the flow of information from endothelial sources to other cell types, we see a significant increase in CDH5, JAM, LAMININ, and COLLAGEN signaling (Figure 4C). Of note, there is little communication based on LAMININ signaling from endothelial cells in NF samples, which expands drastically when looking at the ICM condition (Figure 4D). This seems largely driven by an increase in the ligands *LAMA4*, *LAMB1*, and *LAMC1* in several endothelial subclusters, leading to broad communication with many other cell types (Figure 4E).

Proteomic comparison identifies genes and proteins associated with fibrosis

Next, to understand how transcriptional changes at single-nuclei resolution translate to bulk level protein expression, we compared our snRNA-seq dataset with a previously published bulk proteomic data from ICM patients.[43] When comparing the two datasets we found a number of genes that were significantly dysregulated between ICM and NF in either the snRNA-seq data or the proteomic data, but not changed in both (Figure S6A). However, there was a subset of genes that were significantly changed in both datasets (n = 41) (Figures S6A–S6C; Table S9). Interestingly, many genes that were increased in both datasets were differentially expressed in fibroblasts and associated with ECM formation and fibrosis (*THBS4*, *AEBP1*, *COL14A1*, *VCAN*, *ACTNL1*). We also saw a concordant increase in *ARHGAP1* in cardiomyocytes from ICM. *ARHGAP1* is a promoter of apoptosis and has previously been shown to be increased in cardiomyocytes in response to hypoxia.[44]

We identified 17 genes that were discordant. A high proportion of the genes that were increased in the proteomic data and decreased in the snRNA-seq data were secreted proteins (12/15: *VCAN*, *PRELP*, *C1QB*, *MFGE8*, *A2M*, *FBLN5*, *ERP44*, *AZGP1*, *C3*, *SERPING1*, *SERPINF1*, *SERPINF2*). The two genes that were increased in the snRNA-seq and decreased in the proteomic data were both mitochondrial genes: *HIBADH*, which was differentially expressed in macrophages, and *ALDH5A1*, which was differentially expressed in fibroblasts. This analysis

CellPress
OPEN ACCESS

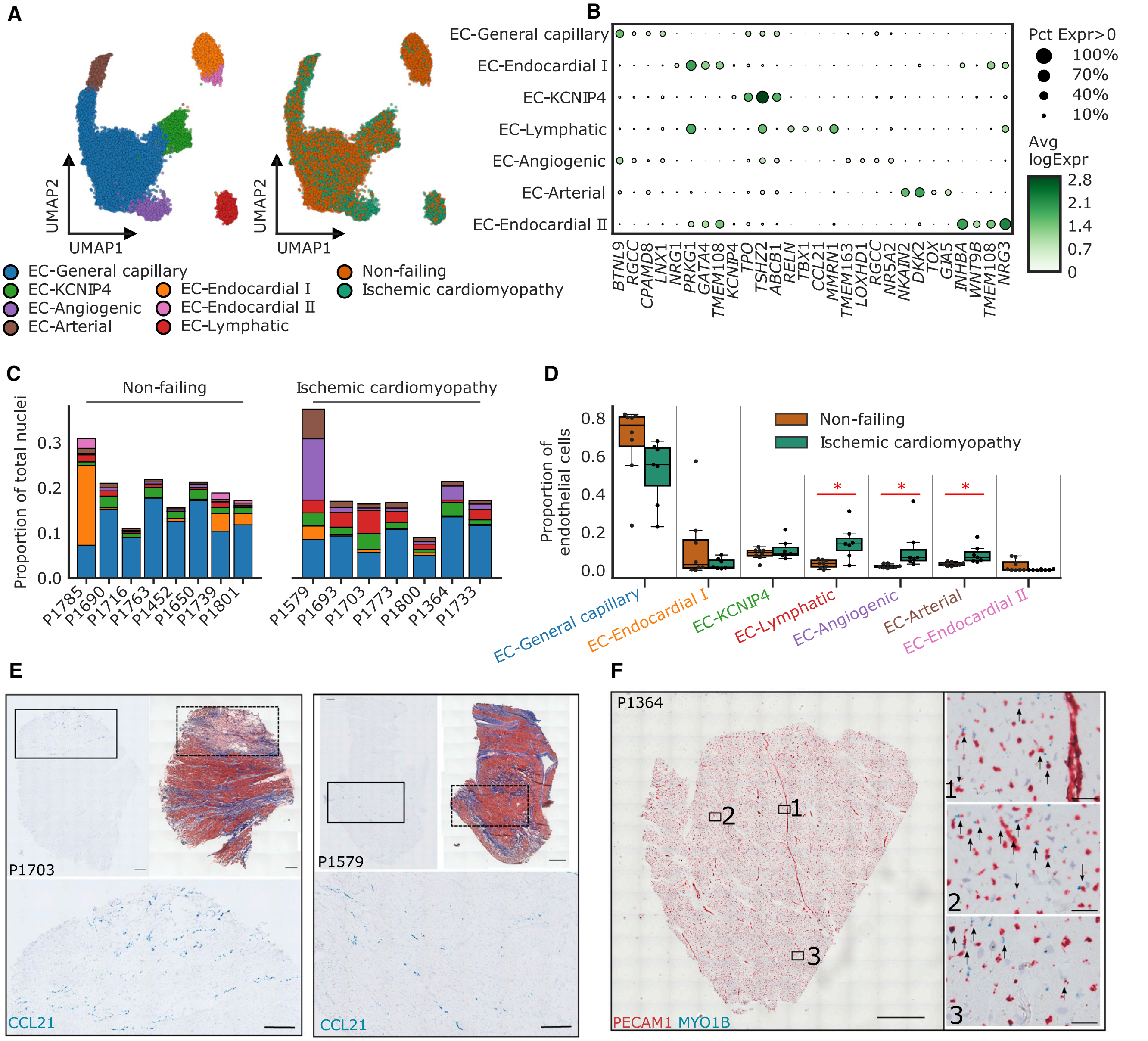

Figure 2. Subclustering of endothelial cells identifies seven distinct subclusters

(A) UMAP of 18,636 endothelial cell identifies 7 subclusters (left) represented across both disease states (right). EC, endothelial cell.

(B) Dot plot of the top marker genes of each endothelial cell subcluster. The size of the dot represents the percent of nuclei expressing the gene at non-zero levels (Pct Expr > 0) and the shading represents the average log-normalized expression (Avg logExpr) of the gene.

(C) Breakdown of endothelial cell composition for each sample relative to the entire composition of all cell types. Subclusters are colored as in (A).

(D) Compositional changes in subclusters between ischemic cardiomyopathy and non-failing samples. Statistically credible increases in EC-lymphatic, EC-angiogenic, and EC-arterial cells were observed in ischemic cardiomyopathy and denoted with an *. Boxplots are represented as: center line, median; box limits, upper and lower quartiles; whiskers, 1.5× interquartile range; points, outliers.

(E) Representative in situ hybridization images of EC-lymphatic cells probed with *CCL21* (blue) alongside trichrome staining of a consecutive section and a cropped close up of an area with high cell numbers. Nuclei are stained with hematoxylin. Scale bars, 1 mm and 250 μm (on close up images). n = 7 (NF) and 6 (ICM).

(F) Representative *in situ* hybridization images of EC-angiogenic cells probed with *PECAM1* (red) and *MYO1B* (blue). Boxes show where close up images were taken and arrows identify cells stained with both *PECAM1* and *MYOB1*. Scale bars, 1 mm and 50 μm (on close up images). Nuclei are stained with hematoxylin (blue). n = 7 (NF) and 6 (ICM).

See also Figures S4 and S5 and Tables S6 and S7.

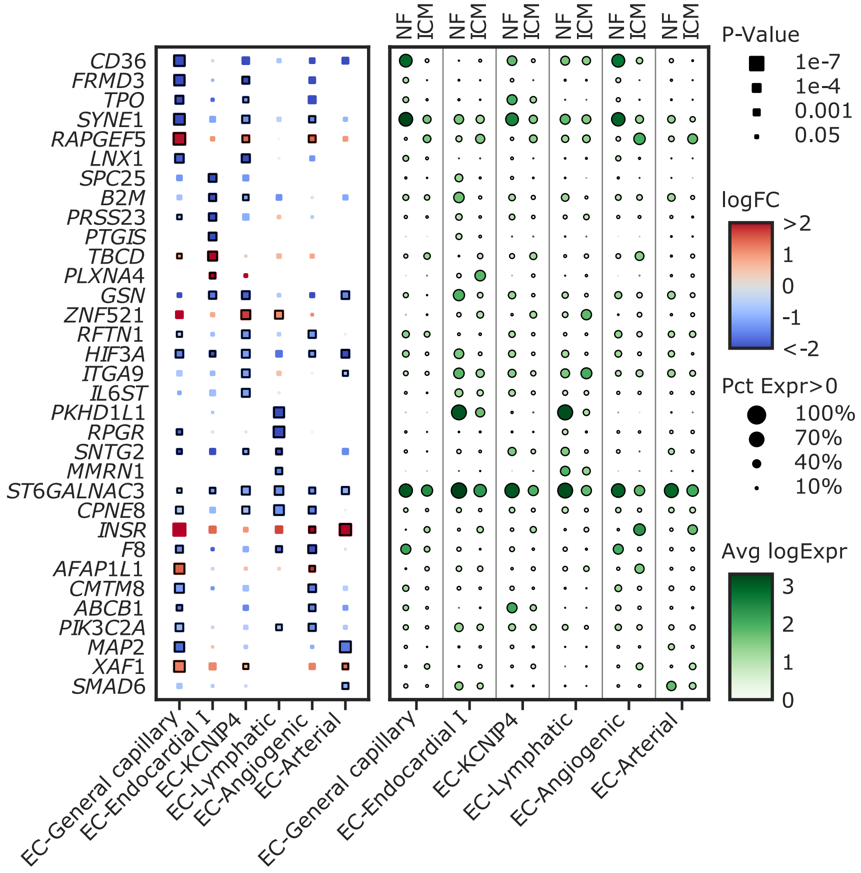

Figure 3. Top differentially expressed protein coding genes in endothelial cell subclusters

Top differentially expressed protein coding genes for each endothelial subcluster. The log fold change (shading) and p value (size) for each gene comparing ICM with NF within each endothelial subcluster is shown on the left. The relative expression in each endothelial subcluster separated by ICM and NF is shown on the right with the size of the dot representing the percent of nuclei expressing the gene and the shade representing the average log-normalized expression. Genes were selected as those expressed in at least 30% of nuclei from either the non-failing or ischemic cardiomyopathy groups with logFC > 1, FDR-adjusted p value < 0.05, and a low probability of being derived from background contamination. NF, non-failing; ICM, ischemic cardiomyopathy; EC, endothelial cell; logFC, log fold change comparing ICM with NF; Pct Expr, percent of nuclei expressing at non-zero levels; Avg logExpr, average log-normalized expression.

See also Table S8.

suggests that there is some correlation between snRNA-seq and proteomics data that may be useful for furthering determining important genes and pathways changed in disease.

Late-stage ICM is transcriptionally similar to late-stage HCM and DCM

Finally, we compared the single-nuclei transcriptional profiles in ICM with a large, recently reported study of snRNA-seq on HCM and DCM samples.[14] In brief, 692,373 nuclei from the ICM dataset and the HCM/DCM dataset were combined and jointly visualized on a shared UMAP representation (Figure 5A). When considering the aggregation of all cell types per sample in a principal-component analysis, we found that the ICM, DCM, and HCM samples clustered together, separate from the NF controls (Figure 5B). We then carried out a cellular composition test of our joint dataset (Figure S7). Overall, cellular composition was similar between DCM, HCM, and ICM, and we observed similar shifts compared with NF patients.

We next wanted to identify genes that were uniquely changed in ICM compared with DCM and HCM. To do this, we identified genes that were significantly changed in ICM vs. NF, as well as differentially expressed between ICM vs. DCM or ICM vs. HCM (Figures 5C and 5D). To control for technical differences between the two studies, we included a covariate representing the study in our differential expression test (see STAR Methods). This identified 17 unique genes, 4 that were significantly differentially expressed both between ICM vs. DCM and ICM vs. HCM, 11 that were significantly differentially expressed only in ICM vs. DCM, and 2 that were significantly differentially expressed only in ICM vs. HCM (Figure 5D). In addition, a small subset of genes were differentially expressed between DCM or HCM vs. NF as well as between DCM or HCM vs. ICM, but not between ICM and NF (Figures 5C and 5D). This included *MDM2*, *KIF9*, and *RSPH6A* in cardiomyocytes, *SELP* in endothelial II, and *TRIM73* in lymphocytes.

There were several interesting genes that were identified in this analysis that were unique to ICM and have been previously associated with ischemia. *CELSR1* was increased in ICM compared with NF and ICM compared with DCM in macrophages. *CELSR1* is an atypical cadherin that is associated with susceptibility to ischemic stroke in Japanese populations[45] and congenital heart disease associated pulmonary arterial hypertension.[46] *IREB2* was increased in pericytes in ICM compared with NF and in ICM compared with DCM and is involved in ferroptosis, a pathway that has been implicated in myocardial infarction and heart disease.[47] *TMLHE* is the first enzyme in the carnitine/acylcarnitine biosynthesis pathway and was decreased in endothelial III cells in ICM compared with NF and in ICM compared with DCM. Knockdown of *TMLHE* is protective in mouse models of ischemia/reperfusion due to decreased long-chain acylcarnitine and ROS production, inhibiting mitochondria and cardiac damage.[48]

There were also changes in genes that have not been previously linked to ICM or heart disease, but still had interesting biological functions. For example, *NPNT* was increased in ICM compared with NF and also in ICM compared with DCM in endothelial II nuclei. *NPNT* is an ECM protein that interestingly has been shown to promote cytokinesis of postnatal CMs.[49] Finally, *DTX3* was decreased in VSMC in ICM compared with NF but increased in

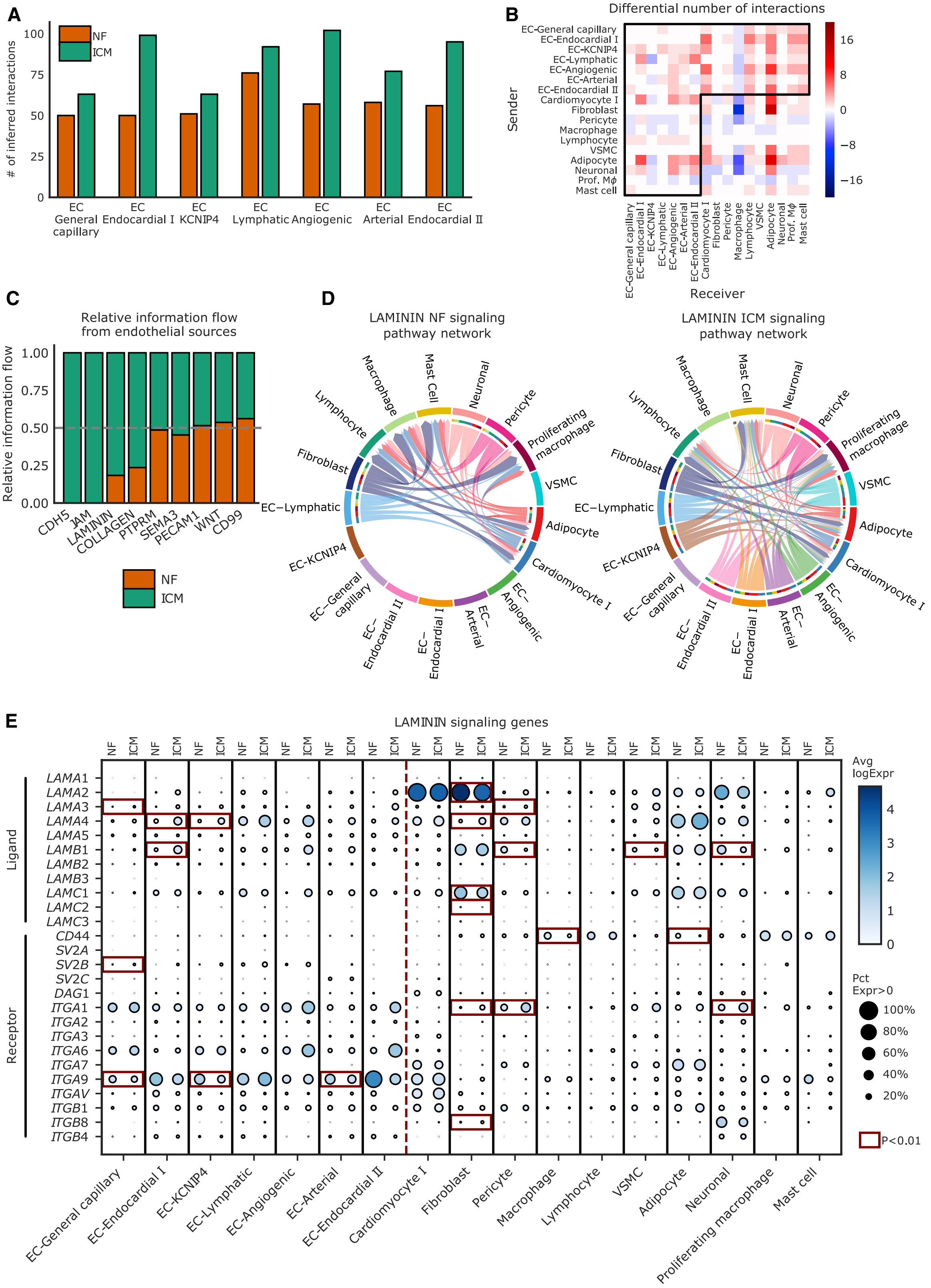

(legend on next page)

both HCM and DCM compared with NF. *DTX3* is an E3 ligase that targets *NOTCH2* and activates proliferation in cancer.[50,51]

We next wanted to explore the similarities observed in the three datasets and looked at the common pathways that were enriched for shared genes between ICM, HCM, and DCM (Figure 5E). We found that many mitochondrial-related pathways were decreased in CMs in disease. We saw an increase in kinase signaling pathways in endothelial I nuclei, with a decrease in signaling by BMP. Interestingly, there were no significant changes observed in fibroblast populations.

Identifying druggable genes for end-stage heart failure

Because of the limited pharmacologic treatments available to treat end-stage heart failure, we sought to identify druggable targets that were present in all three cardiomyopathies. To do so, we compared the list of genes that were strongly, and consistently, up- or downregulated in all cardiomyopathies with the druggable genome.[52] The druggable genome was derived from Finan et al. and includes three tiers of genes based on how similar their features are to known drug targets.[52] We identified 122 druggable genes that were downregulated in all 3 cardiomyopathies, and 52 that were upregulated in all 3 cardiomyopathies (Table S10). We further filtered the list for cell-type specificity (Figure 6A). This led to 10 genes that were druggable and upregulated (Figure 6B) in all 3 cardiomyopathies and 29 genes that were druggable and downregulated in all 3 cardiomyopathies (Figure 6C). Of these genes, *CYD2J2* plays an important compensatory role in heart disease[53] and several were collagens (*COL21A1*, *COL24A1*, *COL14A1*, and *COL23A1*). *MYH6*, a gene well known to be associated with heart failure, was also identified.[54] Several genes were identified that have been previously shown to play important roles in disease that may be druggable and have positive outcomes for heart disease, such as *SMOC2*,[55] *SULF1*,[56] *SPARCL1*,[57] *NRK*,[58] and *PRELP*[59] (Table S11).

Overall, these data suggest that, while there are some differences in late-stage ICM compared with late-stage HCM and DCM, there is a convergence of pathways in late-stage heart disease. By mining the shared enriched pathways and differently expressed genes we are able to identify potential druggable targets for end-stage heart disease.

DISCUSSION

Single cell RNA sequencing and snRNA-seq have allowed the in-depth study of the transcriptome of the healthy and diseased human heart. Here, we carry out snRNA-seq on non-ischemic regions of LV samples from patients with ICM and NF controls. We created a high-quality map of nearly 100,000 nuclei and describe the cellular, transcriptional, and pathway changes observed in ICM compared with NF.

We observed that there was a population of angiogenic endothelial cells (EC-angiogenic) present in capillaries in end-stage disease. Angiogenesis occurs during the initial wound-healing phase of ICM and has been shown to be generally decreased after scar formation.[27] Interestingly, the two samples with the highest proportion of EC-angiogenic cells were the two samples that had the most recent myocardial infarctions (MIs) before transplantation (P1579, 9 months; P1364, 5 months). Increasing blood flow to ischemic tissue by increasing angiogenesis has been a focus of therapeutic development of ICM. Methods such as cell therapy and treatment with VEGF have shown promise in clinical trials, with some improvement in cardiac function observed.[60] The presence of these cell types in late-stage disease suggests that there is still active repair or compensation occurring in the LV of ICM patients.

We were also interested to see the increased number of lymphatic endothelial cells in fibrotic regions of the tissue in ICM compared with NF, especially as these samples are from non-infarcted regions of the LV. Lymphangiogenesis occurs during scar formation after the injury event to help clear fluid, immune cells, and debris from the heart.[27] Like angiogenesis, there has been much interest in increasing lymphangiogenesis as a therapy for ICM. Many studies in animal models have stimulated lymphangiogenesis using VEGF-C after MI, leading to increased clearing of immune cells from damaged tissue, smaller scars, and better heart function[9,61]; however, this response has not always been observed[62,63] and more work is needed in order to understand whether this would be a useful therapeutic pathway to pursue.

Using CellChat, we found a striking increase in LAMININ signaling in ICM from endothelial cells compared with NF, driven largely by increases in the ligands *LAMA4*, *LAMB1*, and *LAMC1* in disease. LAMININ signaling plays a crucial role in attachment of endothelial cells to the basement membrane, and influences the function of cardiomyocytes and other cell types in the heart.[64] Interestingly, mutations and decreased expression of *LAMA4* have been associated with heart failure.[65] *LAMB1* expression has been shown to increase in aging and in a model of acute MI in mice,[66] and has been hypothesized to play a role in pathophysiology in the heart by increasing endothelial cell to mesenchymal cell transition, leading to increased fibrosis.[66] Investigation into the role of endothelial cell signaling to other cell types through LAMININ signaling could help to further characterize the role that endothelial cells play in ICM through their interactions with other cell types.

A limitation of snRNA-seq data is that they only capture the transcriptional activity in the nucleus rather than the whole

Figure 4. CellChat analysis of cell-cell communication between endothelial cells and other cell types

(A) Inferred number of interactions of each subcluster of endothelial cells with all cell types in NF and ICM.

(B) Differential number of interactions of each subcluster of endothelial cell with each cell type, distinguishing between sender and receiver groups. Prof. Mφ, proliferating macrophage.

(C) Relative information flow of signaling from endothelial cells to all cell types within NF or ICM samples.

(D) Chord diagram demonstrating the LAMININ signaling pathway within endothelial cell types and all other cell types, highlighting the increase in signaling in ICM compared with NF. The thickness of each connection represents the strength of communication from the origin cell type to the receiver cell type.

(E) Expression of ligand and receptors involved in LAMININ signaling in endothelial cell subclusters and all other cell clusters. The size of each dot represents the percent of nuclei expression the gene and the shading represents the average normalized expression. Red boxes represent changes between NF and ICM that are significant ($p < 0.01$).

CellPress
OPEN ACCESS

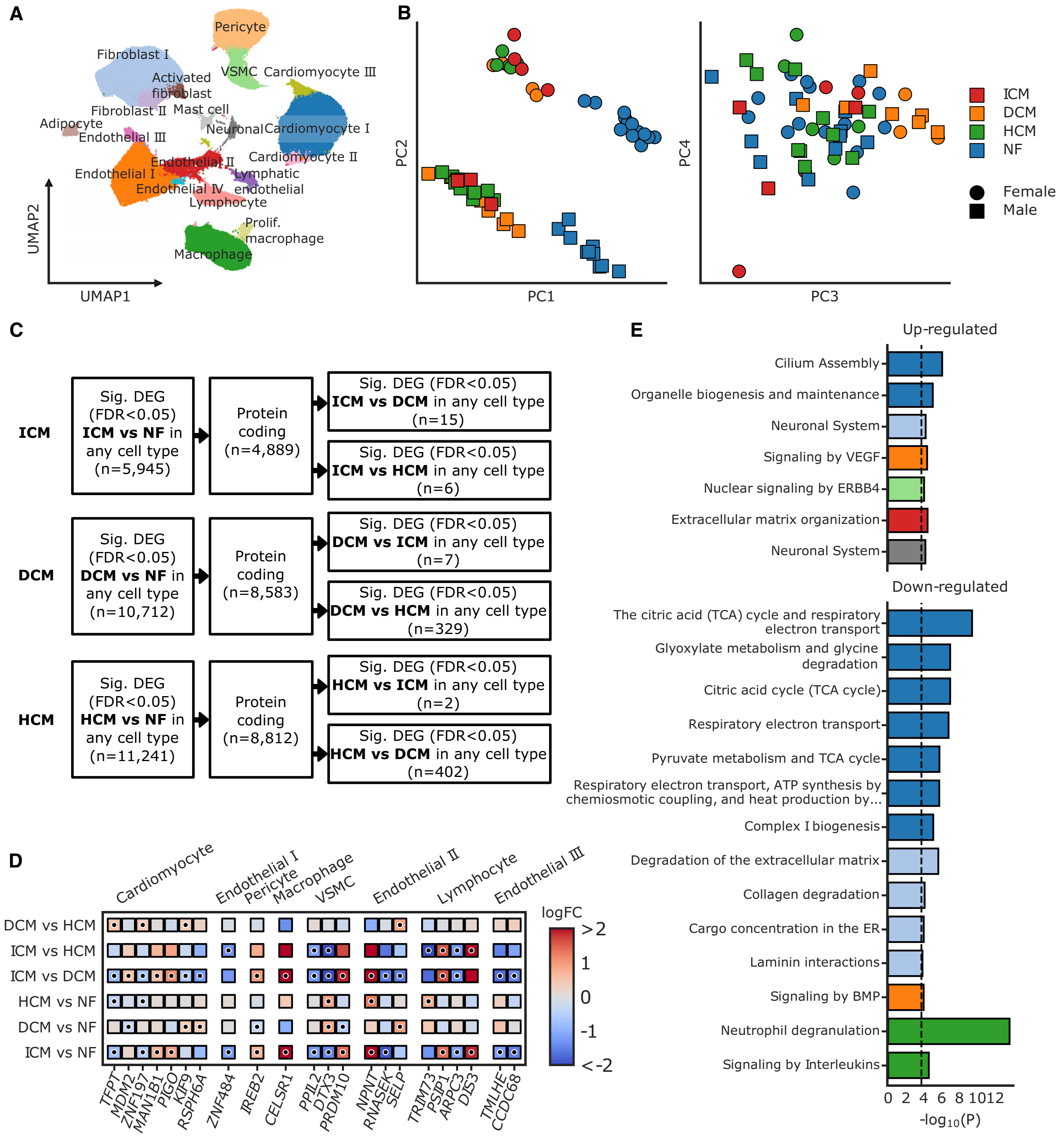

Figure 5. snRNA-seq data on nearly 700,000 nuclei from end-stage cardiomyopathies

(A) Joint UMAP representation of nuclei from hypertrophic cardiomyopathy (HCM), ($n_{patients}$ = 15), dilated cardiomyopathy (DCM) ($n_{patients}$ = 11), ischemic cardiomyopathy (ICM) ($n_{patients}$ = 7), and non-failing (NF) ($n_{patients}$ = 24) samples (n = 692,373) colored by cell-type clusters.

(B) Principal-component (PC) analysis of *pseudo-bulk* expression of all samples (n = 57) as calculated by summing expression across all nuclei in each patient. Colors represent the disease state and shape represents the patient sex.

(C) Flow chart outlining filtering steps carried out to identify protein coding genes that were significantly differentially expressed between any cardiomyopathy and NF, as well as between pairs of cardiomyopathies. DEG, differentially expressed gene; FDR, false discovery rate.

(D) Heatmap of effect size estimates for protein coding genes that were significantly differentially expressed between ICM and NF, as well as ICM and DCM or ICM and HCM (FDR-adjusted p value < 0.05, expressed in >1% of nuclei from either group, low background probability). Also included are genes that were

(legend continued on next page)

cell, which more closely represents the translated proteins. We therefore compared our snRNA-seq dataset and a previously published proteomic study that also used samples taken from non-infarct regions of the LV of transplant recipients with ICM.[43] We found that there was some correlation of gene and protein expression in these samples. As expected, a large number of the proteins that were identified were changed at the mRNA level in cardiomyocytes, fibroblasts, and endothelial cells, the cells that make up the largest portion of the heart. We also saw proteins that showed correlated changes in smaller cell types such as pericytes and neuronal cells. Many of the concordant genes and proteins identified were in fibroblasts. As we observed increased fibrosis in ICM with trichrome staining, and fibrosis plays a large role in ICM this is not particularly surprising. However, more notable was the observation that many of the discordant genes that were decreased at the gene level but increased at the protein level were proteins that are secreted and play a role in the ECM. This could suggest that there is temporal regulation of the mRNA in the nucleus and an accumulation of the secreted proteins in the ECM. One example of a discordant ECM protein was *PRELP*, which was also identified as a druggable gene that was decreased in fibroblasts in all three cardiomyopathies. PRELP protein is increased in the LV of late-stage but not early-stage ICM in pigs[67] and in late-stage ICM in humans[68] and has been suggested to be a biomarker for end-stage heart disease.[59] *PRELP* is thought to be increased in an intermediatory state along the fibroblast myofibroblast transition, and knockout of *PRELP* in fibroblasts leads to decreased activation of fibroblasts after TGB-β stimulation.[14]

The paradigm of the "final common pathway" of heart failure theorizes that all heart failures with reduced ejection fraction converge on the same biological pathway.[69] However, many studies have refuted this idea; for example, bulk RNA-seq[70] and proteomic[43] studies of ICM, DCM, and HCM transplant recipients have shown that there are significant differences between ICM and DCM. Our analysis of ICM compared with HCM and DCM found that, at the nuclear transcriptional level, there are minimal differences in ICM compared with HCM and DCM, supporting the idea of a final common pathway. Current therapies for end-stage heart failure such as implantation of ventricular assist devices and heart transplantation are limited, costly, and highly invasive; therefore, further investigation into the role of these shared pathways and genes in late-stage heart disease may identify areas that could be targeted for therapeutic development. For example, the role of mitochondria dysfunction in the failing heart has been well established,[71] and our data show several mitochondrial-related pathways downregulated in cardiomyocytes across all three cardiomyopathies. Improving mitochondria health and function is a challenging, but active area of interest for pharmacological intervention in heart disease.[72] Integration of snRNA-seq datasets into existing knowledge around mitochondrial dysfunction in heart disease could help to identify key genes and proteins that could be targeted to restore cardiomyocyte bioenergetics.

In summary, we provide an in-depth analysis of the viable left ventricular myocardium of patients with ICM. We found an increased ratio of fibroblasts to cardiomyocytes and many cell-type-specific transcriptional changes, including activation of angiogenesis in endothelial cells and increased lymphatic endothelial cells. We also show changes in signaling from endothelial cells to other cell types in ICM, with an increase in LAMININ signaling. Finally, within each cell type, including cardiomyocytes and fibroblasts, our data support a convergence of the transcriptional profile in advanced heart failure, and we have utilized the druggable genome to identify potential therapeutic targets for end-stage heart failure.

Limitations of the study

We recognize that this study has several limitations, many of which have been discussed previously.[13] The samples we used in this study were from the non-infarct region of the free wall of the LV from ICM patients undergoing heart transplantation or from regions of the free wall of the LV of NF controls. The samples were taken from similar locations from each patient; however, as it is impossible to be exact, there may be variability if there are location-based transcriptional differences, especially in regard to where the sample was taken in relation to the infarct region. Our comparison of snRNA-seq data with proteomic data could be confounded due to the limited number of samples included in the analysis and the samples being taken from different patients. As the samples for this study and the HCM/DCM study were from patients undergoing transplantation, our comparison of transcriptional differences between diseases may be limited as all patients were at end-stage heart failure. ICM may diverge from other forms of heart disease within the infarct and peri-infarct regions. Finally, the composition of the cell types in the ICM samples may be influenced by the fibrotic nature of some of the samples impacting the experimental isolation of nuclei.

STAR★METHODS

Detailed methods are provided in the online version of this paper and include the following:

- KEY RESOURCES TABLE
- RESOURCE AVAILABILITY
  - Lead contact
  - Materials availability
  - Data and code availability
- EXPERIMENTAL MODEL AND SUBJECT DETAILS
  - Human samples

significantly differentially expressed between DCM or HCM and NF, as well as between DCM or HCM and ICM. Shading represents the log fold change (logFC) estimates for each patient population comparison and significant changes are denoted with a dot. VSMC, vascular smooth muscle cell.
(E) Reactome pathways with significant enrichment for genes that were up- or downregulated in all three cardiomyopathies. Multiple testing correction was applied across all cell types/direction combinations using a Benjamini-Hochberg correction (FDR < 0.05, dotted line). Bar colors indicate the cell type in which the significant enrichment was identified, colored as in (A).
See also Figure S7.

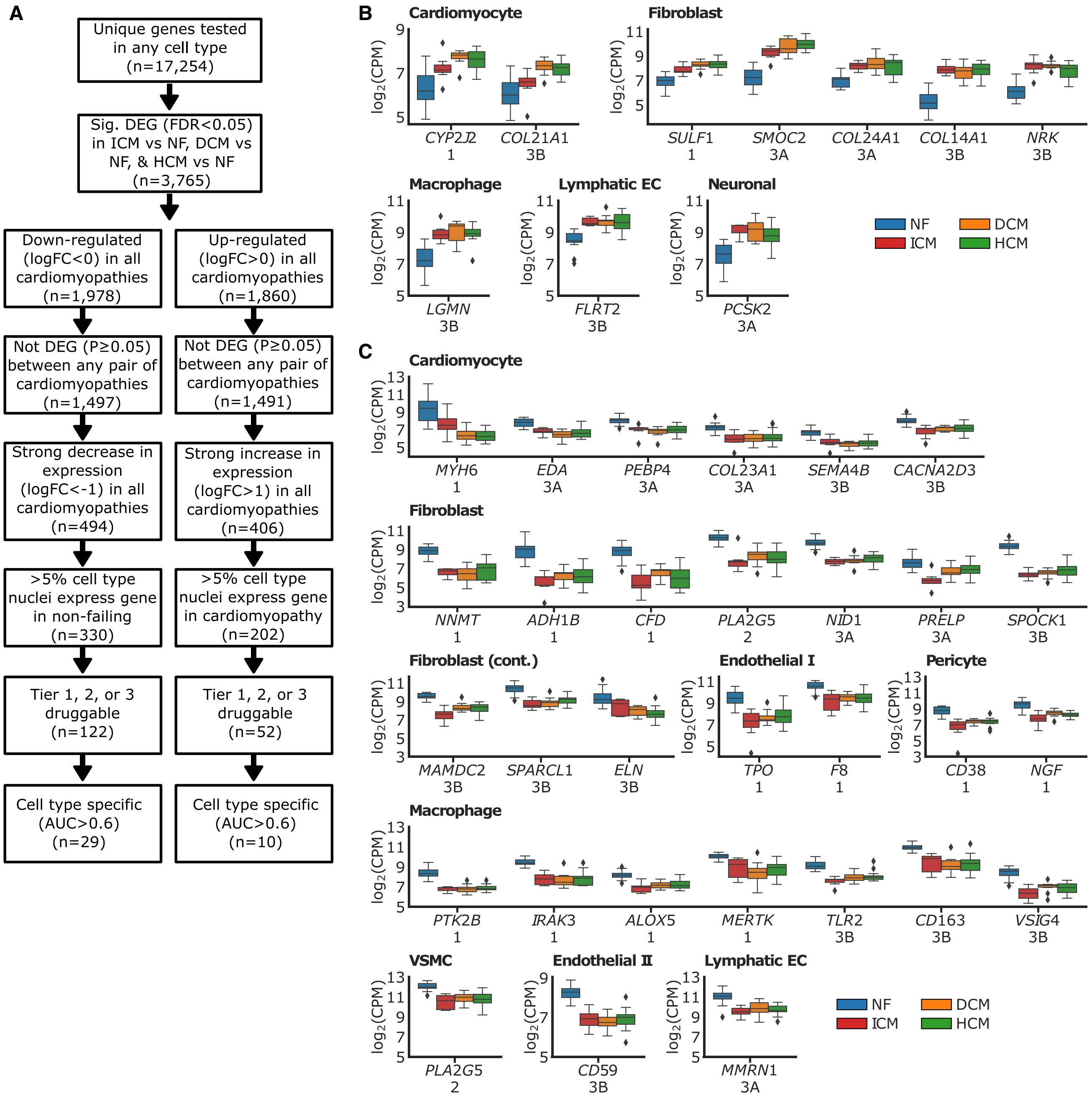

Figure 6. Druggable genes shared by ischemic, hypertrophic, and dilated cardiomyopathies

(A) Flow chart of filtering steps carried out to identify cell-specific druggable genes that are up- or downregulated in ischemic, hypertrophic, and dilated cardiomyopathies. DEG, differentially expressed gene; FDR, false discovery rate; ICM, ischemic cardiomyopathy; NF, non-failing; DCM, dilated cardiomyopathy; HCM, hypertrophic cardiomyopathy; logFC, log fold change; AUC, area under the receiver operating characteristic curve.

(B) Upregulated cell-type-specific druggable genes, with druggable tier noted below.

(C) Downregulated cell-type-specific druggable genes, with druggable tier noted below. Boxplots are represented as: center line, median; box limits, upper and lower quartiles; whiskers, 1.5× interquartile range; points, outliers. CPM, count per million; EC, endothelial cell; VSMC, vascular smooth muscle cell.

See also Tables S10 and S11.

- METHOD DETAILS
  - Single-nuclei RNA sequencing
  - Single-nuclei RNA sequencing data processing
  - Single-nuclei RNA sequencing map aggregation
  - Single-nuclei RNA marker gene calculation
  - Composition analysis
  - Differential expression testing
  - Pathway/ontology analysis

- ○ Endothelial subclustering analysis
- ○ Cell-cell communication analysis
- ○ Proteomics comparison
- ○ Joint ICM, DCM, and HCM snRNA-seq map
- ○ Trichrome staining
- ○ RNAscope validation
- ● QUANTIFICATION AND STATISTICAL ANALYSIS

SUPPLEMENTAL INFORMATION

Supplemental information can be found online at https://doi.org/10.1016/j.celrep.2023.112086.

ACKNOWLEDGMENTS

The Precision Cardiology Laboratory is a joint effort between the Broad Institute and Bayer AG. P.T.E. is supported by grants from the National Institutes of Health (1RO1HL092577, 1R01HL157635, 5R01HL139731), from the American Heart Association Strategically Focused Research Networks (18SFRN34110082), and from the European Union (MAESTRIA 965286). K.B.M. was supported by grants from the National Institutes of Health (1R01HL105993 and 1R01HL149891). Graphical abstract created with art from BioRender.com.

AUTHOR CONTRIBUTIONS

M.C., B.S., C.A.K., and P.T.E. conceived and designed the study. B.S. generated snRNA-seq data. M.C. analyzed the snRNA-seq data. B.S., M.C., M.C.H., A.W.H., S.H., S.B., and P.T.E. interpreted snRNA-seq analyses. O.A. performed RNA *in situ* hybridization. K.C.B. and K.B.M. acquired LV samples for this study. M.C., B.S., M.C.H., and P.T.E drafted the manuscript, which was revised by all authors.

DECLARATION OF INTERESTS

C.A.K. is an employee of Bayer US LLC (a subsidiary of Bayer AG) and may own stock in Bayer. S.H., S.B., and C.A.K. were full-time employees of Bayer when this work was performed. P.T.E. has received sponsored research support from Bayer AG, IBM Health, Bristol Myers Squibb, and Pfizer; he has also served on advisory boards or consulted for Bayer AG, MyoKardia, and Novartis. K.B.M. has research grant funding from Amgen, USA and has also served on advisory boards for MyoKardia, Bristol-Myers Squibb, and Pfizer.

INCLUSION AND DIVERSITY

We support inclusive, diverse, and equitable conduct of research.

Received: May 24, 2022
Revised: November 14, 2022
Accepted: January 23, 2023
Published: February 14, 2023

REFERENCES

1. Khan, M.A., Hashim, M.J., Mustafa, H., Baniyas, M.Y., Al Suwaidi, S.K.B.M., AlKatheeri, R., Alblooshi, F.M.K., Almatrooshi, M.E.A.H., Alzaabi, M.E.H., Al Darmaki, R.S., and Lootah, S.N.A.H. (2020). Global epidemiology of ischemic heart disease: results from the global burden of disease study. Cureus *12*, e9349. https://doi.org/10.7759/cureus.9349.
2. Sutton, M.G., and Sharpe, N. (2000). Left ventricular remodeling after myocardial infarction: pathophysiology and therapy. Circulation *101*, 2981–2988. https://doi.org/10.1161/01.cir.101.25.2981.
3. Levy, D., Kenchaiah, S., Larson, M.G., Benjamin, E.J., Kupka, M.J., Ho, K.K.L., Murabito, J.M., and Vasan, R.S. (2002). Long-term trends in the incidence of and survival with heart failure. N. Engl. J. Med. *347*, 1397–1402. https://doi.org/10.1056/NEJMoa020265.
4. Fu, X., Khalil, H., Kanisicak, O., Boyer, J.G., Vagnozzi, R.J., Maliken, B.D., Sargent, M.A., Prasad, V., Valiente-Alandi, I., Blaxall, B.C., and Molkentin, J.D. (2018). Specialized fibroblast differentiated states underlie scar formation in the infarcted mouse heart. J. Clin. Invest. *128*, 2127–2143. https://doi.org/10.1172/JCI98215.
5. Molenaar, B., Timmer, L.T., Droog, M., Perini, I., Versteeg, D., Kooijman, L., Monshouwer-Kloots, J., de Ruiter, H., Gladka, M.M., and van Rooij, E. (2021). Single-cell transcriptomics following ischemic injury identifies a role for B2M in cardiac repair. Commun. Biol. *4*, 146. https://doi.org/10.1038/s42003-020-01636-3.
6. Singhal, A.K., Symons, J.D., Boudina, S., Jaishy, B., and Shiu, Y.-T. (2010). Role of endothelial cells in myocardial ischemia-reperfusion injury. Vasc. Dis. Prev. *7*, 1–14. https://doi.org/10.2174/1874120701007010001.
7. Segers, V.F.M., Brutsaert, D.L., and De Keulenaer, G.W. (2018). Cardiac remodeling: endothelial cells have more to say than just NO. Front. Physiol. *9*, 382. https://doi.org/10.3389/fphys.2018.00382.
8. Ren, G., Michael, L.H., Entman, M.L., and Frangogiannis, N.G. (2002). Morphological characteristics of the microvasculature in healing myocardial infarcts. J. Histochem. Cytochem. *50*, 71–79. https://doi.org/10.1177/002215540205000108.
9. Shimizu, Y., Polavarapu, R., Eskla, K.-L., Pantner, Y., Nicholson, C.K., Ishii, M., Brunnhoelzl, D., Mauria, R., Husain, A., Naqvi, N., et al. (2018). Impact of lymphangiogenesis on cardiac remodeling after ischemia and reperfusion injury. J. Am. Heart Assoc. *7*, e009565. https://doi.org/10.1161/JAHA.118.009565.
10. Hou, L., Kim, J.J., Woo, Y.J., and Huang, N.F. (2016). Stem cell-based therapies to promote angiogenesis in ischemic cardiovascular disease. Am. J. Physiol. Heart Circ. Physiol. *310*, H455–H465. https://doi.org/10.1152/ajpheart.00726.2015.
11. Song, L., Chen, X., Swanson, T.A., LaViolette, B., Pang, J., Cunio, T., Nagle, M.W., Asano, S., Hales, K., Shipstone, A., et al. (2020). Lymphangiogenic therapy prevents cardiac dysfunction by ameliorating inflammation and hypertension. Elife *9*, e58376. https://doi.org/10.7554/eLife.58376.
12. Litviňuková, M., Talavera-López, C., Maatz, H., Reichart, D., Worth, C.L., Lindberg, E.L., Kanda, M., Polanski, K., Heinig, M., Lee, M., et al. (2020). Cells of the adult human heart. Nature *588*, 466–472. https://doi.org/10.1038/s41586-020-2797-4.
13. Tucker, N.R., Chaffin, M., Fleming, S.J., Hall, A.W., Parsons, V.A., Bedi, K.C., Akkad, A.-D., Herndon, C.N., Arduini, A., Papangeli, I., et al. (2020). Transcriptional and cellular diversity of the human heart. Circulation *142*, 466–482. https://doi.org/10.1161/CIRCULATIONAHA.119.045401.
14. Chaffin, M., Papangeli, I., Simonson, B., Akkad, A.-D., Hill, M.C., Arduini, A., Fleming, S.J., Melanson, M., Hayat, S., Kost-Alimova, M., et al. (2022). Single-nuclei profiling of human dilated and hypertrophic cardiomyopathy. Nature *608*, 174–180. https://doi.org/10.1038/s41586-022-04817-8.
15. Koenig, A.L., Shchukina, I., Amrute, J., Andhey, P.S., Zaitsev, K., Lai, L., Bajpai, G., Bredemeyer, A., Smith, G., Jones, C., et al. (2022). Single-cell transcriptomics reveals cell-type-specific diversification in human heart failure. Nat. Cardiovasc. Res. *1*, 263–280. https://doi.org/10.1038/s44161-022-00028-6.
16. Hanna, A., and Frangogiannis, N.G (2019). The role of the TGF-β superfamily in myocardial infarction. Front. Cardiovasc. Med. *6*, 140. https://doi.org/10.3389/fcvm.2019.00140.
17. Scruggs, A.M., Grabauskas, G., and Huang, S.K. (2020). The role of KCNMB1 and BK channels in myofibroblast differentiation and pulmonary fibrosis. Am. J. Respir. Cell Mol. Biol. *62*, 191–203. https://doi.org/10.1165/rcmb.2019-0163OC.
18. Cao, T.H., Quinn, P.A., Sandhu, J.K., Voors, A.A., Lang, C.C., Parry, H.M., Mohan, M., Jones, D.J.L., and Ng, L.L. (2015). Identification of novel biomarkers in plasma for prediction of treatment response in patients with heart failure. Lancet *385*, S26. https://doi.org/10.1016/S0140-6736(15)60341-5.

19. Tian, X., Sun, C., Wang, X., Ma, K., Chang, Y., Guo, Z., and Si, J. (2020). ANO1 regulates cardiac fibrosis via ATI-mediated MAPK pathway. Cell Calcium *92*, 102306. https://doi.org/10.1016/j.ceca.2020.102306.

20. Zhang, L., Malik, S., Kelley, G.G., Kapiloff, M.S., and Smrcka, A.V. (2011). Phospholipase Cε scaffolds to muscle-specific A kinase anchoring protein (mAKAPβ) and integrates multiple hypertrophic stimuli in cardiac myocytes. J. Biol. Chem. *286*, 23012–23021. https://doi.org/10.1074/jbc.M111.231993.

21. Sörensen-Zender, I., Bhayana, S., Susnik, N., Rolli, V., Batkai, S., Baisantry, A., Bahram, S., Sen, P., Teng, B., Lindner, R., et al. (2015). Zinc-α2-Glycoprotein exerts antifibrotic effects in kidney and heart. J. Am. Soc. Nephrol. *26*, 2659–2668. https://doi.org/10.1681/ASN.2014050485.

22. Gil-Cayuela, C., López, A., Martínez-Dolz, L., González-Juanatey, J.R., Lago, F., Roselló-Lletí, E., Rivera, M., and Portolés, M. (2019). The altered expression of autophagy-related genes participates in heart failure: NRBP2 and CALCOCO2 are associated with left ventricular dysfunction parameters in human dilated cardiomyopathy. PLoS One *14*, e0215818. https://doi.org/10.1371/journal.pone.0215818.

23. Piehler, A., Kaminski, W.E., Wenzel, J.J., Langmann, T., and Schmitz, G. (2002). Molecular structure of a novel cholesterol-responsive A subclass ABC transporter, ABCA9. Biochem. Biophys. Res. Commun. *295*, 408–416. https://doi.org/10.1016/s0006-291x(02)00659-9.

24. González-Domínguez, É., Samaniego, R., Flores-Sevilla, J.L., Campos-Campos, S.F., Gómez-Campos, G., Salas, A., Campos-Peña, V., Corbí, Á.L., Sánchez-Mateos, P., and Sánchez-Torres, C. (2015). CD163L1 and CLEC5A discriminate subsets of human resident and inflammatory macrophages in vivo. J. Leukoc. Biol. *98*, 453–466. https://doi.org/10.1189/jlb.3HI1114-531R.

25. Ashburner, M., Ball, C.A., Blake, J.A., Botstein, D., Butler, H., Cherry, J.M., Davis, A.P., Dolinski, K., Dwight, S.S., Eppig, J.T., et al. (2000). Gene ontology: tool for the unification of biology. The Gene Ontology Consortium. Nat. Genet. *25*, 25–29. https://doi.org/10.1038/75556.

26. Jassal, B., Matthews, L., Viteri, G., Gong, C., Lorente, P., Fabregat, A., Sidiropoulos, K., Cook, J., Gillespie, M., Haw, R., et al. (2020). The reactome pathway knowledgebase. Nucleic Acids Res. *48*, D498–D503. https://doi.org/10.1093/nar/gkz1031.

27. Ishikawa, Y., Akishima-Fukasawa, Y., Ito, K., Akasaka, Y., Tanaka, M., Shimokawa, R., Kimura-Matsumoto, M., Morita, H., Sato, S., Kamata, I., and Ishii, T. (2007). Lymphangiogenesis in myocardial remodelling after infarction. Histopathology *51*, 345–353. https://doi.org/10.1111/j.1365-2559.2007.02785.x.

28. Feng, W., Chen, L., Nguyen, P.K., Wu, S.M., and Li, G. (2019). Single cell analysis of endothelial cells identified organ-specific molecular signatures and heart-specific cell populations and molecular features. Front. Cardiovasc. Med. *6*, 165. https://doi.org/10.3389/fcvm.2019.00165.

29. Kalucka, J., de Rooij, L.P.M.H., Goveia, J., Rohlenova, K., Dumas, S.J., Meta, E., Conchinha, N.V., Taverna, F., Teuwen, L.-A., Veys, K., et al. (2020). Single-cell transcriptome atlas of murine endothelial cells. Cell *180*, 764–779.e20. https://doi.org/10.1016/j.cell.2020.01.015.

30. Schupp, J.C., Adams, T.S., Cosme, C., Raredon, M.S.B., Yuan, Y., Omote, N., Poli, S., Chioccioli, M., Rose, K.-A., Manning, E.P., et al. (2021). Integrated single-cell atlas of endothelial cells of the human lung. Circulation *144*, 286–302. https://doi.org/10.1161/CIRCULATIONAHA.120.052318.

31. Heikkilä, M., Pasanen, A., Kivirikko, K.I., and Myllyharju, J. (2011). Roles of the human hypoxia-inducible factor (HIF)-3α variants in the hypoxia response. Cell. Mol. Life Sci. *68*, 3885–3901. https://doi.org/10.1007/s00018-011-0679-5.

32. Yamashita, T., Ohneda, O., Nagano, M., Iemitsu, M., Makino, Y., Tanaka, H., Miyauchi, T., Goto, K., Ohneda, K., Fujii-Kuriyama, Y., et al. (2008). Abnormal heart development and lung remodeling in mice lacking the hypoxia-inducible factor-related basic helix-loop-helix PAS protein NEPAS. Mol. Cell Biol. *28*, 1285–1297. https://doi.org/10.1128/MCB.01332-07.

33. King, S.J., Nowak, K., Suryavanshi, N., Holt, I., Shanahan, C.M., and Ridley, A.J. (2014). Nesprin-1 and nesprin-2 regulate endothelial cell shape and migration. Cytoskeleton *71*, 423–434. https://doi.org/10.1002/cm.21182.

34. Escudero, C.A., Herlitz, K., Troncoso, F., Guevara, K., Acurio, J., Aguayo, C., Godoy, A.S., and González, M. (2017). Pro-angiogenic role of insulin: from physiology to pathology. Front. Physiol. *8*, 204. https://doi.org/10.3389/fphys.2017.00204.

35. Li, G.H., Shi, Y., Chen, Y., Sun, M., Sader, S., Maekawa, Y., Arab, S., Dawood, F., Chen, M., De Couto, G., et al. (2009). Gelsolin regulates cardiac remodeling after myocardial infarction through DNase I-mediated apoptosis. Circ. Res. *104*, 896–904. https://doi.org/10.1161/CIRCRESAHA.108.172882.

36. Liston, P., Fong, W.G., Kelly, N.L., Toji, S., Miyazaki, T., Conte, D., Tamai, K., Craig, C.G., McBurney, M.W., and Korneluk, R.G. (2001). Identification of XAF1 as an antagonist of XIAP anti-Caspase activity. Nat. Cell Biol. *3*, 128–133. https://doi.org/10.1038/35055027.

37. Galvin, K.M., Donovan, M.J., Lynch, C.A., Meyer, R.I., Paul, R.J., Lorenz, J.N., Fairchild-Huntress, V., Dixon, K.L., Dunmore, J.H., Gimbrone, M.A., et al. (2000). A role for smad6 in development and homeostasis of the cardiovascular system. Nat. Genet. *24*, 171–174. https://doi.org/10.1038/72835.

38. Tan, B., Liu, M., Yang, Y., Liu, L., and Meng, F. (2019). Low expression of PIK3C2A gene: a potential biomarker to predict the risk of acute myocardial infarction. Medicine (Baltim.) *98*, e15061. https://doi.org/10.1097/MD.0000000000015061.

39. Teixeira, S.C., Lopes, D.S., Gimenes, S.N.C., Teixeira, T.L., da Silva, M.S., Brígido, R.T.E.S., da Luz, F.A.C., da Silva, A.A., Silva, M.A., Florentino, P.V., et al. (2017). Mechanistic insights into the anti-angiogenic activity of trypanosoma cruzi protein 21 and its potential impact on the onset of chagasic cardiomyopathy. Sci. Rep. *7*, 44978. https://doi.org/10.1038/srep44978.

40. Leatherdale, A., Parker, D., Tasneem, S., Wang, Y., Bihan, D., Bonna, A., Hamaia, S.W., Gross, P.L., Ni, H., Doble, B.W., et al. (2021). Multimerin 1 supports platelet function in vivo and binds to specific GPAGPOGPX motifs in fibrillar collagens that enhance platelet adhesion. J. Thromb. Haemost. *19*, 547–561. https://doi.org/10.1111/jth.15171.

41. Zheng, C., Quan, R., Xia, E.-J., Bhandari, A., and Zhang, X. (2019). Original tumour suppressor gene polycystic kidney and hepatic disease 1-like 1 is associated with thyroid cancer cell progression. Oncol. Lett. *18*, 3227–3235. https://doi.org/10.3892/ol.2019.10632.

42. Jin, S., Guerrero-Juarez, C.F., Zhang, L., Chang, I., Ramos, R., Kuan, C.-H., Myung, P., Plikus, M.V., and Nie, Q. (2021). Inference and analysis of cell-cell communication using CellChat. Nat. Commun. *12*, 1088. https://doi.org/10.1038/s41467-021-21246-9.

43. Chen, C.Y., Caporizzo, M.A., Bedi, K., Vite, A., Bogush, A.I., Robison, P., Heffler, J.G., Salomon, A.K., Kelly, N.A., Babu, A., et al. (2018). Suppression of detyrosinated microtubules improves cardiomyocyte function in human heart failure. Nat. Med. *24*, 1225–1233. https://doi.org/10.1038/s41591-018-0046-2.

44. Zhang, X., Guan, J., Guo, M., Dai, H., Cai, S., Zhou, C., Wang, Y., and Qin, Q. (2019). Rho GTPase-activating protein 1 promotes apoptosis of myocardial cells in an ischemic cardiomyopathy model. Kardiol. Pol. *77*, 1163–1169. https://doi.org/10.33963/KP.15040.

45. Yamada, Y., Fuku, N., Tanaka, M., Aoyagi, Y., Sawabe, M., Metoki, N., Yoshida, H., Satoh, K., Kato, K., Watanabe, S., et al. (2009). Identification of CELSR1 as a susceptibility gene for ischemic stroke in Japanese individuals by a genome-wide association study. Atherosclerosis *207*, 144–149. https://doi.org/10.1016/j.atherosclerosis.2009.03.038.

46. Eyries, M., Girerd, B., Savale, L., Soubrier, F., Humbert, M., and Montani, D. (2021). A CELSR1 variant in a patient with pulmonary arterial hypertension. Clin. Genet. *100*, 771–772. https://doi.org/10.1111/cge.14046.

47. Wu, X., Li, Y., Zhang, S., and Zhou, X. (2021). Ferroptosis as a novel therapeutic target for cardiovascular disease. Theranostics *11*, 3052–3059. https://doi.org/10.7150/thno.54113.

48. Liepinsh, E., Kuka, J., Vilks, K., Svalbe, B., Stelfa, G., Vilskersts, R., Sevostjanovs, E., Goldins, N.R., Groma, V., Grinberga, S., et al. (2021). Low cardiac content of long-chain acylcarnitines in TMLHE knockout mice prevents ischaemia-reperfusion-induced mitochondrial and cardiac damage. Free Radic. Biol. Med. *177*, 370–380. https://doi.org/10.1016/j.freeradbiomed.2021.10.035.
49. Wu, C.-C., Jeratsch, S., Graumann, J., and Stainier, D.Y.R. (2020). Modulation of mammalian cardiomyocyte cytokinesis by the extracellular matrix. Circ. Res. *127*, 896–907. https://doi.org/10.1161/CIRCRESAHA.119.316303.
50. Ding, X.-Y., Hu, H.-Y., Huang, K.-N., Wei, R.-Q., Min, J., Qi, C., Tang, H., and Qin, X. (2020). Ubiquitination of NOTCH2 by DTX3 suppresses the proliferation and migration of human esophageal carcinoma. Cancer Sci. *111*, 489–501. https://doi.org/10.1111/cas.14288.
51. Valla, M., Opdahl, S., Ytterhus, B., and Bofin, A.M. (2021). DTX3 copy number increase in breast cancer: a study of associations to molecular subtype, proliferation and prognosis. Breast Cancer Res. Treat. *187*, 57–67. https://doi.org/10.1007/s10549-021-06138-2.
52. Finan, C., Gaulton, A., Kruger, F.A., Lumbers, R.T., Shah, T., Engmann, J., Galver, L., Kelley, R., Karlsson, A., Santos, R., et al. (2017). The druggable genome and support for target identification and validation in drug development. Sci. Transl. Med. *9*, eaag1166. https://doi.org/10.1126/scitranslmed.aag1166.
53. He, Z., Zhang, X., Chen, C., Wen, Z., Hoopes, S.L., Zeldin, D.C., and Wang, D.W. (2015). Cardiomyocyte-specific expression of CYP2J2 prevents development of cardiac remodelling induced by angiotensin II. Cardiovasc. Res. *105*, 304–317. https://doi.org/10.1093/cvr/cvv018.
54. Carniel, E., Taylor, M.R.G., Sinagra, G., Di Lenarda, A., Ku, L., Fain, P.R., Boucek, M.M., Cavanaugh, J., Miocic, S., Slavov, D., et al. (2005). Alpha-myosin heavy chain: a sarcomeric gene associated with dilated and hypertrophic phenotypes of cardiomyopathy. Circulation *112*, 54–59. https://doi.org/10.1161/CIRCULATIONAHA.104.507699.
55. Xin, C., Lei, J., Wang, Q., Yin, Y., Yang, X., Moran Guerrero, J.A., Sabbisetti, V., Sun, X., Vaidya, V.S., and Bonventre, J.V. (2021). Therapeutic silencing of SMOC2 prevents kidney function loss in mouse model of chronic kidney disease. iScience *24*, 103193. https://doi.org/10.1016/j.isci.2021.103193.
56. Wu, J., Subbaiah, K.C.V., Xie, L.H., Jiang, F., Khor, E.-S., Mickelsen, D., Myers, J.R., Tang, W.H.W., and Yao, P. (2020). Glutamyl-Prolyl-tRNA synthetase regulates proline-rich pro-fibrotic protein synthesis during cardiac fibrosis. Circ. Res. *127*, 827–846. https://doi.org/10.1161/CIRCRESAHA.119.315999.
57. Schellings, M.W.M., Vanhoutte, D., Swinnen, M., Cleutjens, J.P., Debets, J., van Leeuwen, R.E.W., d'Hooge, J., Van de Werf, F., Carmeliet, P., Pinto, Y.M., et al. (2009). Absence of SPARC results in increased cardiac rupture and dysfunction after acute myocardial infarction. J. Exp. Med. *206*, 113–123. https://doi.org/10.1084/jem.20081244.
58. Wei, T., Song, J., Liang, K., Li, L., Mo, X., Huang, Z., Chen, G., Mao, N., and Yang, J. (2021). Identification of a novel therapeutic candidate, NRK, in primary cancer-associated fibroblasts of lung adenocarcinoma microenvironment. J. Cancer Res. Clin. Oncol. *147*, 1049–1064. https://doi.org/10.1007/s00432-020-03489-z.
59. Ahmed, A., Ahmed, S., Arvidsson, M., Bouzina, H., Lundgren, J., and Rådegran, G. (2020). Prolargin and matrix metalloproteinase-2 in heart failure after heart transplantation and their association with haemodynamics. ESC Heart Fail. *7*, 223–234. https://doi.org/10.1002/ehf2.12560.
60. Johnson, T., Zhao, L., Manuel, G., Taylor, H., and Liu, D. (2019). Approaches to therapeutic angiogenesis for ischemic heart disease. J. Mol. Med. *97*, 141–151. https://doi.org/10.1007/s00109-018-1729-3.
61. Henri, O., Pouehe, C., Houssari, M., Galas, L., Nicol, L., Edwards-Lévy, F., Henry, J.-P., Dumesnil, A., Boukhalfa, I., Banquet, S., et al. (2016). Selective stimulation of cardiac lymphangiogenesis reduces myocardial edema and fibrosis leading to improved cardiac function following myocardial infarction. Circulation *133*, 1484–1497. https://doi.org/10.1161/CIRCULATIONAHA.115.020143.
62. Houssari, M., Dumesnil, A., Tardif, V., Kivelä, R., Pizzinat, N., Boukhalfa, I., Godefroy, D., Schapman, D., Hemanthakumar, K.A., Bizou, M., et al. (2020). Lymphatic and immune cell cross-talk regulates cardiac recovery after experimental myocardial infarction. Arterioscler. Thromb. Vasc. Biol. *40*, 1722–1737. https://doi.org/10.1161/ATVBAHA.120.314370.
63. Keller, T.C.S., Lim, L., Shewale, S.V., McDaid, K., Martí-Pàmies, Í., Tang, A.T., Wittig, C., Guerrero, A.A., Sterling, S., Leu, N.A., et al. (2021). Genetic blockade of lymphangiogenesis does not impair cardiac function after myocardial infarction. J. Clin. Invest. *131*, e147070. https://doi.org/10.1172/JCI147070.
64. Boland, E., Quondamatteo, F., and Van Agtmael, T. (2021). The role of basement membranes in cardiac biology and disease. Biosci. Rep. *41*, BSR20204185. https://doi.org/10.1042/BSR20204185.
65. Chen, J., Ma, Y., Li, H., Lin, Z., Yang, Z., Zhang, Q., Wang, F., Lin, Y., Ye, Z., and Lin, Y. (2022). Rare and potential pathogenic mutations of LMNA and LAMA4 associated with familial arrhythmogenic right ventricular cardiomyopathy/dysplasia with right ventricular heart failure, cerebral thromboembolism and hereditary electrocardiogram abnormality. Orphanet J. Rare Dis. *17*, 183. https://doi.org/10.1186/s13023-022-02348-z.
66. Wagner, J.U.G., Chavakis, E., Rogg, E.-M., Muhly-Reinholz, M., Glaser, S.F., Günther, S., John, D., Bonini, F., Zeiher, A.M., Schaefer, L., et al. (2018). Switch in laminin β2 to laminin β1 isoforms during aging controls endothelial cell functions—brief report. Arterioscler. Thromb. Vasc. Biol. *38*, 1170–1177. https://doi.org/10.1161/ATVBAHA.117.310685.
67. Barallobre-Barreiro, J., Didangelos, A., Schoendube, F.A., Drozdov, I., Yin, X., Fernández-Caggiano, M., Willeit, P., Puntmann, V.O., Aldama-López, G., Shah, A.M., et al. (2012). Proteomics analysis of cardiac extracellular matrix remodeling in a porcine model of ischemia/reperfusion injury. Circulation *125*, 789–802. https://doi.org/10.1161/CIRCULATIONAHA.111.056952.
68. Yi, X., Jiang, D.-S., Feng, G., Jiang, X.-J., and Zeng, H.-L. (2019). An altered left ventricle protein profile in human ischemic cardiomyopathy revealed in comparative quantitative proteomics. Kardiol. Pol. *77*, 951–959. https://doi.org/10.33963/KP.14936.
69. Bowles, N.E., Bowles, K.R., and Towbin, J.A. (2000). The "final common pathway" hypothesis and inherited cardiovascular disease. The role of cytoskeletal proteins in dilated cardiomyopathy. Herz *25*, 168–175. https://doi.org/10.1007/s000590050003.
70. Sweet, M.E., Cocciolo, A., Slavov, D., Jones, K.L., Sweet, J.R., Graw, S.L., Reece, T.B., Ambardekar, A.V., Bristow, M.R., Mestroni, L., and Taylor, M.R.G. (2018). Transcriptome analysis of human heart failure reveals dysregulated cell adhesion in dilated cardiomyopathy and activated immune pathways in ischemic heart failure. BMC Genom. *19*, 812. https://doi.org/10.1186/s12864-018-5213-9.
71. Knowlton, A.A., Chen, L., and Malik, Z.A. (2014). Heart failure and mitochondrial dysfunction: the role of mitochondrial fission/fusion abnormalities and new therapeutic strategies. J. Cardiovasc. Pharmacol. *63*, 196–206. https://doi.org/10.1097/01.fjc.0000432861.55968.a6.
72. Brown, D.A., Perry, J.B., Allen, M.E., Sabbah, H.N., Stauffer, B.L., Shaikh, S.R., Cleland, J.G.F., Colucci, W.S., Butler, J., Voors, A.A., et al. (2017). Mitochondrial function as a therapeutic target in heart failure. Nat. Rev. Cardiol. *14*, 238–250. https://doi.org/10.1038/nrcardio.2016.203.
73. Martin, M. (2011). Cutadapt removes adapter sequences from high-throughput sequencing reads. EMBnet. j. *17*, 10.
74. Fleming, S.J., Marioni, J.C., and Babadi, M. (2019). CellBender remove-background: a deep generative model for unsupervised removal of background noise from scRNA-seq datasets. Preprint at bioRxiv. https://doi.org/10.1101/791699.
75. Wolf, F.A., Angerer, P., and Theis, F.J. (2018). SCANPY: large-scale single-cell gene expression data analysis. Genome Biol. *19*, 15. https://doi.org/10.1186/s13059-017-1382-0.
76. Korsunsky, I., Millard, N., Fan, J., Slowikowski, K., Zhang, F., Wei, K., Baglaenko, Y., Brenner, M., Loh, P.-R., and Raychaudhuri, S. (2019). Fast,

CellPress
OPEN ACCESS

sensitive and accurate integration of single-cell data with Harmony. Nat. Methods *16*, 1289–1296. https://doi.org/10.1038/s41592-019-0619-0.

77. Nemenman, I., Shafee, F., and Bialek, W. (2002). Entropy and inference, revisited. Preprint at arXiv. https://doi.org/10.48550/arXiv.physics/0108025.
78. Wolock, S.L., Lopez, R., and Klein, A.M. (2019). Scrublet: computational identification of cell doublets in single-cell transcriptomic data. Cell Syst. *8*, 281–291.e9. https://doi.org/10.1016/j.cels.2018.11.005.
79. Law, C.W., Chen, Y., Shi, W., and Smyth, G.K. (2014). voom: Precision weights unlock linear model analysis tools for RNA-seq read counts. Genome Biol. *15*, R29. https://doi.org/10.1186/gb-2014-15-2-r29.
80. Ritchie, M.E., Phipson, B., Wu, D., Hu, Y., Law, C.W., Shi, W., and Smyth, G.K. (2015). Limma powers differential expression analyses for RNA-sequencing and microarray studies. Nucleic Acids Res. *43*, e47. https://doi.org/10.1093/nar/gkv007.
81. Love, M.I., Huber, W., and Anders, S. (2014). Moderated estimation of fold change and dispersion for RNA-seq data with DESeq2. Genome Biol. *15*, 550. https://doi.org/10.1186/s13059-014-0550-8.
82. Büttner, M., Ostner, J., Müller, C.L., Theis, F.J., and Schubert, B. (2021). scCODA is a Bayesian model for compositional single-cell data analysis. Nat. Commun. *12*, 6876. https://doi.org/10.1038/s41467-021-27150-6.
83. Chen, Y., Lun, A.T.L., and Smyth, G.K. (2016). From reads to genes to pathways: differential expression analysis of RNA-Seq experiments using Rsubread and the edgeR quasi-likelihood pipeline. F1000Res. *5*, 1438. https://doi.org/10.12688/f1000research.8987.2.
84. Alexa, A., Rahnenführer, J., and Lengauer, T. (2006). Improved scoring of functional groups from gene expression data by decorrelating GO graph structure. Bioinformatics *22*, 1600–1607. https://doi.org/10.1093/bioinformatics/btl140.
85. Yu, G., and He, Q.-Y. (2016). ReactomePA: an R/Bioconductor package for reactome pathway analysis and visualization. Mol. Biosyst. *12*, 477–479. https://doi.org/10.1039/c5mb00663e.
86. Durinck, S., Spellman, P.T., Birney, E., and Huber, W. (2009). Mapping identifiers for the integration of genomic datasets with the R/Bioconductor package biomaRt. Nat. Protoc. *4*, 1184–1191. https://doi.org/10.1038/nprot.2009.97.
87. Schneider, C.A., Rasband, W.S., and Eliceiri, K.W. (2012). NIH Image to ImageJ: 25 years of image analysis. Nat. Methods *9*, 671–675. https://doi.org/10.1038/nmeth.2089.
88. Stuart, T., Butler, A., Hoffman, P., Hafemeister, C., Papalexi, E., Mauck, W.M., Hao, Y., Stoeckius, M., Smibert, P., and Satija, R. (2019). Comprehensive integration of single-cell data. Cell *177*, 1888–1902.e21. https://doi.org/10.1016/j.cell.2019.05.031.
89. Satija, R., Farrell, J.A., Gennert, D., Schier, A.F., and Regev, A. (2015). Spatial reconstruction of single-cell gene expression data. Nat. Biotechnol. *33*, 495–502. https://doi.org/10.1038/nbt.3192.
90. Tirosh, I., Izar, B., Prakadan, S.M., Wadsworth, M.H., Treacy, D., Trombetta, J.J., Rotem, A., Rodman, C., Lian, C., Murphy, G., et al. (2016). Dissecting the multicellular ecosystem of metastatic melanoma by single-cell RNA-seq. Science *352*, 189–196. https://doi.org/10.1126/science.aad0501.
91. Lun, A.T.L., and Marioni, J.C. (2017). Overcoming confounding plate effects in differential expression analyses of single-cell RNA-seq data. Biostatistics *18*, 451–464. https://doi.org/10.1093/biostatistics/kxw055.
92. The Gene Ontology Consortium (2019). The gene ontology resource: 20 years and still GOing strong. Nucleic Acids Res. *47*, D330–D338. https://doi.org/10.1093/nar/gky1055.

STAR★METHODS

KEY RESOURCES TABLE

REAGENT or RESOURCE	SOURCE	IDENTIFIER
**Chemicals, peptides, and recombinant proteins**		
Sucrose	Sigma Aldrich	Cat# 57903-250g
Tris HCl	Sigma Aldrich	Cat# 10812846001
KCl	Sigma Aldrich	Cat# P9333-1kg
MgCl2.6H2O	Sigma Aldrich	Cat# M2670-500g
DTT	Thermo Scientific	Cat# 20291
IGEPAL	Sigma Aldrich	Cat# 18896-50mL
10% BSA	Miltenyi Biotech	Cat# 130-091-376
RNAse Inhibitor Murine	New England Biolabs	Cat# MO314L
16% PFA	Electron Microscopy Sciences	Cat# 15710
Permanent mounting media	Vectamount	Cat# H-5000
**Critical commercial assays**		
Chromium Single Cell 3′ reagent kit, v 3	10x Genomics	Cat# 1000075
Chromium Single Cell B Chip kit, 48 rxn	10x Genomics	Cat# 1000073
Chromium i7 Multiplex Kit, 96 rxn	10s Genomics	Cat# 120262
Trichrome stain kit	Abcam	Cat# 150686
RNAscope 2.5 HD Duplex reagent Kit	ACD bio	Cat# 322430
RNAscope probe hs-CCL21	ACD bio	Cat# 474371
RNAscope probe hs-PECAM1	ACD bio	Cat# 487381-C2
RNAscope probe hs-MYO1B	ACD bio	Cat# 444301
**Deposited data**		
Processed snRNA-seq data h5ad	This paper	Broad Institute's Single Cell Portal: SCP1849 (https://singlecell.broadinstitute.org/single_cell/study/SCP1849)
Raw snRNA-seq FASTQ	This paper	dbGaP: phs001539.v4.p1; SRA: PRJNA433594
**Software and algorithms**		
Custom snRNA-seq analysis code	This paper	Zenodo: https://doi.org/10.5281/zenodo.7469682; GitHub: https://github.com/mark-chaffin/snrnaseq_ischemic_cardiomyopathy
R 3.6.0; 4.1	R Foundation	https://www.r-project.org/
Python 3.7.3	https://www.python.org/	https://www.python.org/
Cell Ranger 4.0.0	10X Genomics	https://support.10xgenomics.com/single-cell-gene-expression/software/overview/welcome
cutadapt 1.18	Martin, 2011[73]	https://github.com/marcelm/cutadapt
CellBender 0.2	Fleming et al., 2019[74]	https://github.com/broadinstitute/CellBender
scR-Invex	https://github.com/broadinstitute/scrinvex	https://github.com/broadinstitute/scrinvex
scanpy 1.7.2	Wolf et al., 2018[75]	https://github.com/scverse/scanpy
harmony-pytorch 0.1.4	Korsunsky et al., 2019[76]	https://github.com/lilab-bcb/harmony-pytorch
ndd 1.6.3	Nemenman et al., 2002[77]	https://github.com/simomarsili/ndd
Scrublet 0.2.1	Wolock et al., 2019[78]	https://github.com/swolock/scrublet

(*Continued on next page*)

Continued

REAGENT or RESOURCE	SOURCE	IDENTIFIER
limma 3.44.3	Law et al., 2014[79]<br>Ritchie et al., 2015[80]	https://doi.org/10.18129/B9.bioc.limma
DESeq2 1.20.0	Love et al., 2014[81]	https://doi.org/10.18129/B9.bioc.DESeq2
scCODA 0.1.2.post	Büttner et al., 2021[82]	https://github.com/theislab/scCODA
edgeR 3.30.3	Chen et al., 2016[83]	https://doi.org/10.18129/B9.bioc.edgeR
topGO 2.32.0	Alexa et al., 2006[84]	https://doi.org/10.18129/B9.bioc.topGO
ReactomePA 1.24.0	Yu and He, 2016[85]	https://doi.org/10.18129/B9.bioc.ReactomePA
biomaRt 2.36.1	Durinck et al., 2009[86]	https://doi.org/10.18129/B9.bioc.biomaRt
CellChat 1.5.0	Jin et al., 2021[41]	https://github.com/sqjin/CellChat
GraphPad Prism	Dotmatics	Graphpad.com
(Fuji is just) ImageJ	Schneider et al., 2012[87]	https://imagej.nih.gov

RESOURCE AVAILABILITY

Lead contact

Further information and requests for resources and reagents should be directed to and will be fulfilled by the lead contact, Patrick Ellinor (ellinor@mgh.harvard.edu).

Materials availability

This study did not generate new unique reagents.

Data and code availability

- Processed single-nuclei transcriptomic data have been deposited at the Broad Institute's Single Cell Portal: https://singlecell.broadinstitute.org/single_cell/study/SCP1849/ and are publicly available as of the date of publication. Accession numbers are listed in the key resources table. Raw sequence data have been deposited at dbGaP (the database of Genotypes and Phenotypes, accession number phs001539.v4.p1) and Sequence Read Archive (SRA) and are available to authorized users. These accession numbers are also listed in the key resources table. Microscopy data reported in this paper will be shared by the lead contact upon request.
- Original code for snRNA-seq analysis, including the global map construction, differential expression analysis, and endothelial subclustering has been deposited at Zenodo: https://doi.org/10.5281/zenodo.7469682 and GitHub: https://github.com/mark-chaffin/snrnaseq_ischemic_cardiomyopathy. DOIs are listed in the key resources table. Any additional analysis was performed using publicly available programs and analytical packages as outlined in the STAR Methods and key resources table.
- Any additional information required to reanalyze the data reported in this paper is available from the lead contact upon request.

EXPERIMENTAL MODEL AND SUBJECT DETAILS

Human samples

Adult human left ventricular tissue was collected from heart transplant recipients (for the ICM cases) and from deceased organ donors (for the NF cases), as previously described.[13] All hearts were arrested *in situ* with at least 1 L of ice-cold crystalloid cardioplegia solution, and transported in ice-cold cardioplegia solution to the lab where they were cryopreserved (within 4 h). Specimens were collected in a transmural fashion (epicardium - > endocardium) from the LV free wall below the level of the papillary muscle and adjacent to the septum and above the LV apex. The donor and transplant recipients were collected identically, though it is impossible to be from the exact same spot across samples at different times. We did not purposefully collect the border zone and infarct region in this study. The age and sex of the samples were as follows: NF: M 52, F 64, M 58, M 58, F 47, F 63, F 53, M 42, ICM: F 64, F 55, M 55, F 62, F 47, M 64, F 62. All other relevant patient information can be found in Table 1. Samples collected in the HCM and DCM study were collected in a similar manner.

Written informed consent for research use of donated tissue was obtained from the transplant recipients or next of kin (for the NF cases). Research use of tissues were approved by the relevant institutional review boards at the University of Pennsylvania, Gift-of-Life Donor Program, and Massachusetts General Hospital and the Broad Institute.

METHOD DETAILS

Single-nuclei RNA sequencing

Single nucleus suspensions were generated as previously described.[13] In brief, approximately 100 mg of left ventricular tissue was cryosectioned (100 μm) and placed into 2 mL of ice-cold NIM2 buffer (250 mM sucrose, 10 mM Tris pH 8.0, 25 mM KCl, 5 mM MGCL2, 1 μM DTT, 0.01% IGEPAL). Samples were tritiated with a cut tip, transferred to a 7 mL dounce and homogenized on ice. Samples were made up to 4 mL with NIM2 buffer and large debris pelleted by centrifugation at 40 x g at 4° for 2 min. The supernatant was filtered through stacked 40 μm and 10 μm filters. Filters were washed with 6 mL of PBS wash buffer (0.01% BSA, 5 mM MgCl2, PBS) and the nuclei pelleted by centrifugation at 550 x g at 4° for 5 min. The nuclei pellet was washed by removal of the supernatant, and addition of 6 mL of PBS wash buffer, followed by centrifugation at 550 x g at 4° for 5 min. The supernatant was removed and nuclei resuspended in 150 μL of nuclei resuspension buffer (0.01% BSA, 5 mM MgCl2, 0.4 U/uL RNase inhibitor), and the nuclei were counted using a hemocytometer. Approximately 6000 nuclei per sample were loaded onto a 10x genomics microfluidic chip and samples were processed according to the 10x genomics protocol (Chromium Single cell 3′, v3) with the following changes. First, nuclei were incubated at 4C for 15 min after emulsion generation to promote nuclear lysis. Second, the reverse transcription protocol was modified for one of the two replicates to be 42C for 20 min then 53C for 120 min. Libraries were multiplexed at an average of 16 libraries per flow cell on an Illumina Novaseq S1 at the Broad Institute's Genomics Platform.

Single-nuclei RNA sequencing data processing

Initial data processing was performed using the 10x Genomics toolkit CellRanger v4.0.0. First, FASTQ files were generated from demultiplexed raw base call files with *cellranger mkfastq*. Homopolymer repeats greater than 30 base pairs as well as the template switch oligo and its reverse complement (AAGCAGTGGTATCAACGCAGAGTACATGGG, CCCATGTACTCTGCGTTGATACCACTGCTT) were trimmed using *cutadapt*[73] with parameters max_error_rate = 0.1, min_overlap = 20 and max_error_rate = 0.07, min_overlap = 10, respectively. Reads were then aligned to the GRCh38 pre-mRNA human reference (v2020-A) with *cellranger count* with default parameters setting –expect-cells 6000.

Samples were assessed for quality using three criteria. First, *cellranger count* metrics were compared across samples to identify extreme outliers (Table S1). One sample, P1795, had an abnormally low percent of valid barcodes (<90%) and was removed. Second, the relationship between total unique molecular identifiers (UMIs) per nucleus and the rank of nuclei by total UMI was visually inspected to ensure a clear distinction between non-empty and empty droplets and acceptable levels of ambient RNA. Third, individual Uniform Manifold Approximation and Projection (UMAP) plots were inspected for each sample to identify any lacking clear structure. Finally, the total count of Y chromosome genes was calculated for each sample and correlated with phenotypic sex. In total, 1 of 16 samples was removed.

For the remaining samples, we applied the tool *remove-background* from CellBender v0.2, https://github.com/broadinstitute/CellBender [74] to call non-empty droplets and subtract ambient background RNA contamination. Parameters were set to: –expected-cells 6000, –z-dim 100, –total-droplets-included 25,000, –epochs 150, –learning_rate 1e-4, –fpr 0.01. For one sample, P1693, the –total-droplets-included parameter was set to 30,000 to ensure model convergence.

Finally, we calculated the fraction of reads from each cell barcode that mapped purely to exonic regions using the tool *scR-Invex* (https://github.com/broadinstitute/scrinvex). This metric can serve as a surrogate for cytoplasmic contamination as reads derived from the cytoplasmic should map predominantly to exonic regions, whereas reads from the nucleus will span both introns and exons.

Single-nuclei RNA sequencing map aggregation

Single-nuclei RNA sequencing map aggregation was performed analogously to Chaffin et al.[14] All analysis was performed in *scanpy* v1.7.2[75] unless otherwise specified. First, non-empty droplets called with CellBender *remove-background* were aggregated across samples. The top 2000 highly variable genes were identified using the methodology from Seurat V3.[88] Counts were log-normalized per nucleus to 10,000 counts and scaled per gene to unit variance and zero mean. The first 50 principal components were estimated using 2000 highly variable genes and aligned across samples to account for biological and technical heterogeneity using *Harmony* as implemented in *harmony-pytorch* v0.1.4.[76] A neighborhood graph was computed with *sc.pp.neighbors()* using cosine distance. Data was projected using the UMAP method with the effective minimum distance between embedded points set to 0.2. High resolution Leiden clustering (resolution = 2.0) was performed to cluster nuclei into groups.

To remove low quality nuclei, four quality control QC metrics were employed: 1. The fraction of UMI mapping to mitochondrial genes based on CellRanger counts (%MT), 2. The fraction of reads mapping exclusively to exonic regions (exon_prop), 3. A measure of nuclei-based entropy as calculated using the *ndd* python library (https://pypi.org/project/ndd/1.6.3/)[77] and 4. A predicted doublet score as estimated using *Scrublet*[78] with default settings based on CellRanger counts (doublet_score). Clusters from this preliminary map with elevated levels of %MT, exon_prop, or doublet_score or abnormally low levels of entropy were removed which accounted for 47,115/176,518 (26.7%) nuclei. Remaining clusters were further cleaned by performing a per sample, per cluster procedure based on the distribution of each QC metric defined by the first quartile (Q1), the third quartile (Q3), and the interquartile range (IQR). For each sample/cluster combination, outlier nuclei were identified as those with 1. Total UMI (n_umi) greater than Q3 + 1.5*IQR or less than Q1 – 1.5*IQR, 2. Number of unique genes (n_genes) greater than Q3 + 1.5*IQR or less than Q1 – 1.5*IQR, 3. %MT greater than Q3 + 1.5*IQR, 4. Entropy greater than Q3 + 1.5*IQR or less than Q1 – 1.5*IQR, 5. exon_prop greater than Q3 + 1.5*IQR, 6. doublet_score

greater than Q3 + 1.5*IQR, or 7. log(n_genes) * entropy greater than Q3 + 1.5*IQR. When a given sample/cluster combination had less than 30 nuclei, hard thresholds were set at 150 $\leq$ n_umi $\leq$15,000, 150 $\leq$ n_genes $\leq$6000, %MT $\leq$ 5%, entropy $\geq$8, exon_prop $\leq$ 0.18, doublet_score $\leq$0.30, log(n_gene) * entropy $\leq$75. This removed an additional 28,879 nuclei.

The remaining 102,524 nuclei were re-aggregated using the same approach as above after reducing the resolution of Leiden clustering to 0.5. As a final pruning step, a sub-clustering procedure was performed. First, similar clusters were joined, and marker genes were calculated for each major cell type based on an area under the receiver operating characteristic curve (AUC) comparing the expression in each major cell type to all other cell types. Each major cell type was then sub-clustered by recalculating the neighborhood graph on the harmony adjusted PCs. Then, Leiden clustering was performed at increasing resolutions of 0.05–1.0, in increments of 0.05. At each step, marker genes for each sub-cluster compared to all other sub-clusters were calculated using the AUC metric. When a sub-cluster emerged and had no genes with AUC greater than 0.6, sub-clustering was halted. All remaining sub-clusters were then scored based on the top 50 marker genes of the global cell types, as determined by AUC, as well as all mitochondrial genes using the *sc.tl.score_genes()* function in *scanpy*[89,90]. Any sub-clusters of a cell type enriched in scores from other major cell types or mitochondrial genes were deemed low quality or misclassified and subsequently removed. This resulted in the removal of an additional 2,840 nuclei and a final snRNA-seq map containing 99,684 nuclei.

Single-nuclei RNA marker gene calculation

To assign cell type labels to each cluster, marker genes for each Leiden cluster were calculated compared to all other clusters using two approaches. First, an AUC was calculated for each gene comparing the log-normalized expression at the nuclei level in each cluster against all other clusters. Second, a formal differential expression model controlling for the correlation amongst nuclei from the same individual was performed by summing gene counts across all nuclei in a cluster within an individual patient and treating the data as a bulk RNA sequencing experiment. Nuclei were only aggregated in an individual if there were more than 20 nuclei of the given cluster. Next, a differential expression model was performed using the limma-voom framework[79,80] with a model of ∼0 + cluster + individual with DESeq2 normalization[81] to control for the fact that each individual is represented across clusters. An individual contrast was extracted comparing each cluster to all other clusters. Marker genes for each cluster were then identified as protein coding genes with an AUC >0.60, a Benjamini-Hochberg corrected p value from the limma-voom model <0.01, a log fold-change estimate from the limma-voom model greater than 2 and expressed in at least 25% of nuclei in the given cluster.

Composition analysis

To identify differences in cell type composition between ischemic and non-failing samples, we applied the Bayesian method from *scCODA* v0.1.2.post1.[82] For this analysis, a reference cell type must be selected that is deemed to be relatively invariant across samples. In the global compositional test, pericytes were selected as they had the smallest variation across samples and were reasonably prevalent in the dataset. When testing for changes in endothelial cell composition, the reference group was selected as EC-*KCNIP4*.

Differential expression testing

We performed differential expression between ICM and NF samples treating each patient as the unit of biological replication. To control for the fact that nuclei derived from a given patient will be correlated, we took the approach of Lun and Marioni.[91] For each patient, we calculated the sum across nuclei for each gene within a given cell type of interest and treated the data as if it were derived from a bulk RNA-sequencing experiment. We required a patient to have at least 25 nuclei of the given cell type to be included. We removed genes in <1% of nuclei from both ICM and NF samples, separately, or identified as lowly expressed by filterByExpr() in edgeR based on the summed counts.[83] We further removed mitochondrial and ribosomal genes from differential expression testing. Expression was normalized using DESeq2[81] and differential expression testing was performed using the limma-voom testing framework.[79,80] All tests were adjusted for age and sex. We applied false discovery rate (FDR) correction using the Benjamini-Hochberg procedure. Our primary analysis was performed on CellBender adjusted counts, but as a sensitivity analysis, we repeated our test using CellBender unadjusted counts. Only genes with FDR-adjusted p-values <0.05 in both tests were considered significant.

Additionally, we tested for an association between the percent of fibrosis, as estimated using Trichrome staining, and gene expression among the ICM patients. Only the 6 ICM patients with successful Trichrome staining were included. Differential expression was tested as described above, adjusting for age and sex. As this was considered an exploratory analysis due to the limited power of the statistical test, we focus on genes that have an unadjusted p-value <0.005 from both CellBender and CellRanger.

To minimize the role of background contamination in dictating our differential expression results, we calculated a per gene, per cell type background contamination heuristic. For example, if a gene is highly expressed in a prevalent cell type (e.g. cardiomyocytes) and differentially expressed between ICM and NF patients, we may observe differential expression from the background in other cell types that express low levels of the gene. This heuristic consisted of two components: 1. A measure of overall expression for each gene in the dataset, and 2. A measure of specificity of each gene to the given cell type being tested. For the first component, we divided the total UMI of each gene in the entire dataset by the total UMI in the dataset. We then estimated a value (“bkg_prob”) for each gene along the cumulative distribution of these values. For the second component, we calculated two positive predictive values for each gene, standardized for equal prevalence of cell type, by dichotomizing expression as >0 (PPV0) or >1 (PPV1) and predicting classification into the cell type where differential expression test was performed. We then calculated “nontarget_prob” as 1 - mean(PPV0,PPV1). Our final heuristic for a given gene in a given cell type was calculated as bkg_prob * nontarget_prob. Genes

with values >0.4 were considered as having a high probability of being derived from background and flagged or removed from downstream analyses.

Pathway/ontology analysis

Pathway analyses were performed using two databases, gene ontology (GO) biological processes (BP)[25,92] and Reactome pathways.[26] Marker gene pathway enrichment was performed on GO BP with topGO[84] using both a Kolmogorov-Smirnov (KS) test and Fisher's exact test with the weight01 algorithm to account for the GO graph. For the KS test, genes for each cluster were ranked on the t-statistic from the differential expression test. For Fisher's exact test, selected marker genes for each cluster were compared against all genes tested. Only GO terms with greater than 15 and less than 500 genes were considered. Ontologies with a p-value <0.001 from the Fisher's exact test and odds ratio >2 were considered significant. A multiple testing correction was not applied because GO term enrichment is calculated conditionally on neighboring terms and therefore not all tests are independent.

Pathway enrichment for genes differentially expressed in the ICM versus NF comparison was tested using both GO BP databases with topGO[84] and Reactome pathways using ReactomePA.[85] Genes were separated into significantly upregulated in ICM and significantly downregulated in ICM prior to enrichment testing. All genes with a background contamination heuristic >0.4 in a given cell type were removed. For GO BP terms, we used a Fisher's exact test with the weight01 algorithm to account for the GO graph in topGO and only considered ontologies with greater than 15 and less than 500 genes. Ontologies with a p-value <0.001 and an odds ratio for enrichment >2 were considered significant. For Reactome pathways, we employed a hypergeometric test using ReactomePA with a minimum gene set size of 10 and a maximum gene set size of 500. A Benjamini-Hochberg correction was applied to ReactomePA results across all cell type/direction combinations and pathways with FDR <0.05 were considered significant.

Endothelial subclustering analysis

All 20,462 nuclei from global clusters Endothelial I, Endothelial II, Endothelial III, and Lymphatic endothelial were sub-clustered by the following procedure. First, we recalculated Harmony-adjusted principal components as described in the global map construction but restricted to just these 20,462 nuclei. Next, we calculated a neighborhood graph using cosine distance and 10 nearest neighbors. Clusters were determined with the Leiden algorithm at various resolutions, starting at 0.10 and increasing by increments of 0.10. When a cluster emerged with no genes that had an AUC >0.60 separating it from all other clusters, sub-clustering was halted. Sub-clusters were inspected for enrichment in non-endothelial cell markers and mitochondrial gene expression. Any misclassified or low-quality sub-clusters were removed. This process was repeated 3 times and removed a total of 1,826 (9%) of nuclei. To determine the final cluster identities, we performed hierarchical clustering with Euclidean distance and the Ward method based on the mean expression of the 2000 highly variable genes within each sub-cluster. Sub-clusters with distance less than (0.25 * max inter-cluster distance) were merged.

Marker genes were calculated as in the global map. As sub-clusters tend to be more related than global cell populations, the marker gene criteria were loosened to protein coding genes with an AUC >0.50, a Benjamini-Hochberg corrected p value from the limma-voom model <0.01, a log fold-change estimate from the limma-voom model greater than 0, and expressed in at least 15% of nuclei in the given cluster. Gene ontology enrichment for marker genes was calculated as described in the Pathway/Ontology analysis section. Additionally, to compare sub-clusters of endothelial cells to previously published data, we scored all endothelial cells based on profiles from up to 50 of the top marker genes (as determined by log fold-change) of endothelial sub-clusters from Feng et al.,[28] Kalucka et al.,[29] and Schupp et al.[30] using the *sc.tl.score_genes()* function. Mouse genes were mapped to human orthologs biomaRt v2.36.1.[86]

To identify up- and down-regulated genes in ICM patients compared to NF patients with endothelial sub-clusters, we performed differential expression testing as described above. To calculate the background contamination heuristic, we separated nuclei into endothelial cells (Endothelial I, Endothelial II, Endothelial III, and Lymphatic endothelial) and non-endothelial cells and performed the same calculation as described above.

Cell-cell communication analysis

Communication between endothelial cell subtypes and other cell types was quantified using CellChat.[42] Expression was log-normalized, and nuclei were separated into ICM and NF. Cell types or subtypes with less than 10 nuclei in either disease group were excluded. We used CellChat to then infer the cell-cell communication network within each disease state based on upregulated ligands/receptors in cell types. We then compared the signaling networks in ICM to the signaling networks in NF, focused on endothelial subtypes. Specifically, we compared the number of interactions involving each endothelial subtype broadly, as well as changes in communication between specific pairs of cell types. We then looked for differences in signaling pathways by comparing the information flow from endothelial cells which was defined as the sum of communication probabilities with any endothelial source to any cell type.

Proteomics comparison

We compared changes in at the RNA level in each cell type between ICM and NF based on our snRNA-seq data to changes in protein expression in left ventricles between ICM and NF reported in Chen et al.[43] All log fold-change and p value estimates for genes in the snRNA-seq in each cell type were compared directly to the published estimates from the bulk proteomics data.

CellPress
OPEN ACCESS

Joint ICM, DCM, and HCM snRNA-seq map

We constructed a joint map of snRNA-seq data coming from ICM and NF patients in this study with additional snRNA-seq derived from dilated cardiomyopathy (DCM), hypertrophic cardiomyopathy (HCM), and additional NF patients from Chaffin et al.[14] In brief, CellBender-adjusted counts from the 99,684 ICM and NF nuclei were aggregated with an additional 592,689 nuclei from DCM, HCM, and NF patients. The map was constructed analogously as described in "Single-nuclei RNA sequencing map aggregation", aligning across the patient identifier of nuclei using *Harmony*. Leiden clustering was performed at resolution 0.6.

Principal component analysis was then performed on the patient-level by summing gene counts across all nuclei per patient and treating the data as a bulk experiment. Mitochondrial genes, ribosomal genes, and genes expressed in <1% of nuclei were removed. Remaining genes were normalized with DESeq2 and genes with less than 10 total counts were removed. A variance stabilizing transformation was applied with *vst()*.[81] Principal components were calculated with *prcomp()* in *R*.

Differential expression analysis was performed on a cell-type basis for 6 pairwise comparisons: ICM vs NF, DCM vs NF, HCM vs NF, ICM vs DCM, ICM vs HCM, and DCM vs HCM. Testing was performed as described in "Differential expression testing between ICM and NF" by first summing counts across all nuclei per patient and the fitting the model Expr ~1 + disease + sex + age + study using the limma-voom framework[79,80] where study indicates whether the sample is derived from the ICM vs NF study or the DCM/HCM vs NF study. This last covariate allows us to control for technical differences between studies by assuming that NF patients from each study should be comparable. For each cell type, a contrast was specified for each of the 6 pairwise comparisons described above. Nuclei were only summed in a patient if there were at least 25 nuclei of the given cell type and cell types were only tested if there were at least 3 samples in the two groups compared. Multiple testing correction was performed using the Benjamini-Hochberg procedure within each pairwise comparison at an FDR <0.05.

To identify shared pathways between ICM, DCM, and HCM, we performed a Reactome pathway enrichment analysis focusing on genes that were differentially expressed in all cardiomyopathies, per cell type. Genes significantly dysregulated in all three cardiomyopathies (based on both CellBender and CellRanger counts) were split into those consistently upregulated across cardiomyopathy subtypes and those consistently downregulated across cardiomyopathy subtypes. Pathway analysis was performed on these sets using ReactomePA as described above. A Benjamini-Hochberg correction was applied to ReactomePA results across all cell type/direction combinations and pathways with FDR <0.05 were considered significant.

To identify shared druggable targets across all cardiomyopathies, we performed a series of filtering steps on genes tested for differential expression. For each cell type, we identified genes that were significantly differentially expressed (FDR <0.05 based on Benjamini-Hochberg procedure) in ICM versus NF, DCM versus NF, and HCM versus NF. We split these into up- and down-regulated genes based on the log fold-change estimate. We further removed genes that were significantly differentially expressed between any pair of cardiomyopathies, reduced this to genes with a strong effect (absolute value of log fold-change >1), and reasonably expressed in either cardiomyopathy or NF (>5% of nuclei). We then annotated these genes by whether they were druggable based on Finan et al.[52] and looked for cell-type specificity based on AUC.

Trichrome staining

Trichrome staining was carried out on 10 μm sections following product protocol (Abcam: ab150686) and whole sections were imaged and tiled at 10x (Zeiss Axio Observer Z1). Area of fibrosis was quantified using thresholding on (Fuji is just) ImageJ v2.1.0.

RNAscope validation

To image lymphatic endothelial cells and angiogenic endothelial cells, colorimetric RNAscope was carried out on 10 μm fresh frozen tissue sections using the RNAscope 2.5 HD Duplex kit (ACDbio: 322430) following manufacturers protocols. As nearly all angiogenic endothelial cell marker genes were expressed appreciably in other cell types, we used a combination of two probes to identify this population. First, we identified 6 genes that were highly selective to angiogenic endothelial cells compared to other endothelial cell populations (AUC >0.6, log fold-change >1, percent of nuclei expression >20%) and that were not expressed in more than 25% of any other endothelial cell population. As all 6 of these genes showed some expression in other non-endothelial cell types, we calculated the selectivity of these 6 genes combined with expression of the global endothelial marker *PECAM1*. We determined that 23% of angiogenic endothelial cells in our snRNA-seq data expressed *MYO1B* and *PECAM1* whereas double-positives in other endothelial sub-populations and non-endothelial global populations occurred in no more than 6% of nuclei. Therefore, the probes used for this study were hs-CCL21 (474371), hs-PECAM1 (487381-C2), and hs-MYO1B (444301). To quantify the number of lymphatic endothelial cells, (Fuji is just) ImageJ v2.1.0[87] was used to manually count the number of CCL21 positive cells, and then thresholded to automatically count the number of nuclei per section.

QUANTIFICATION AND STATISTICAL ANALYSIS

Graphpad Prism 9.3.1 was used to carry out an unpaired t test on the number of lymphatic endothelial cells identified in the RNAscope experiments (Figure S5). Statistical significance defined as a p value less than 0.5 All additional statistical analyses were performed in Python or R, with details of these analyses described in the main text, STAR Methods, figure legends, and supplemental tables.

Cell Reports, Volume *42*

Supplemental information

Single-nucleus RNA sequencing in ischemic cardiomyopathy reveals common transcriptional profile underlying end-stage heart failure

Bridget Simonson, Mark Chaffin, Matthew C. Hill, Ondine Atwa, Yasmine Guedira, Harshit Bhasin, Amelia W. Hall, Sikander Hayat, Simon Baumgart, Kenneth C. Bedi Jr., Kenneth B. Margulies, Carla A. Klattenhoff, and Patrick T. Ellinor

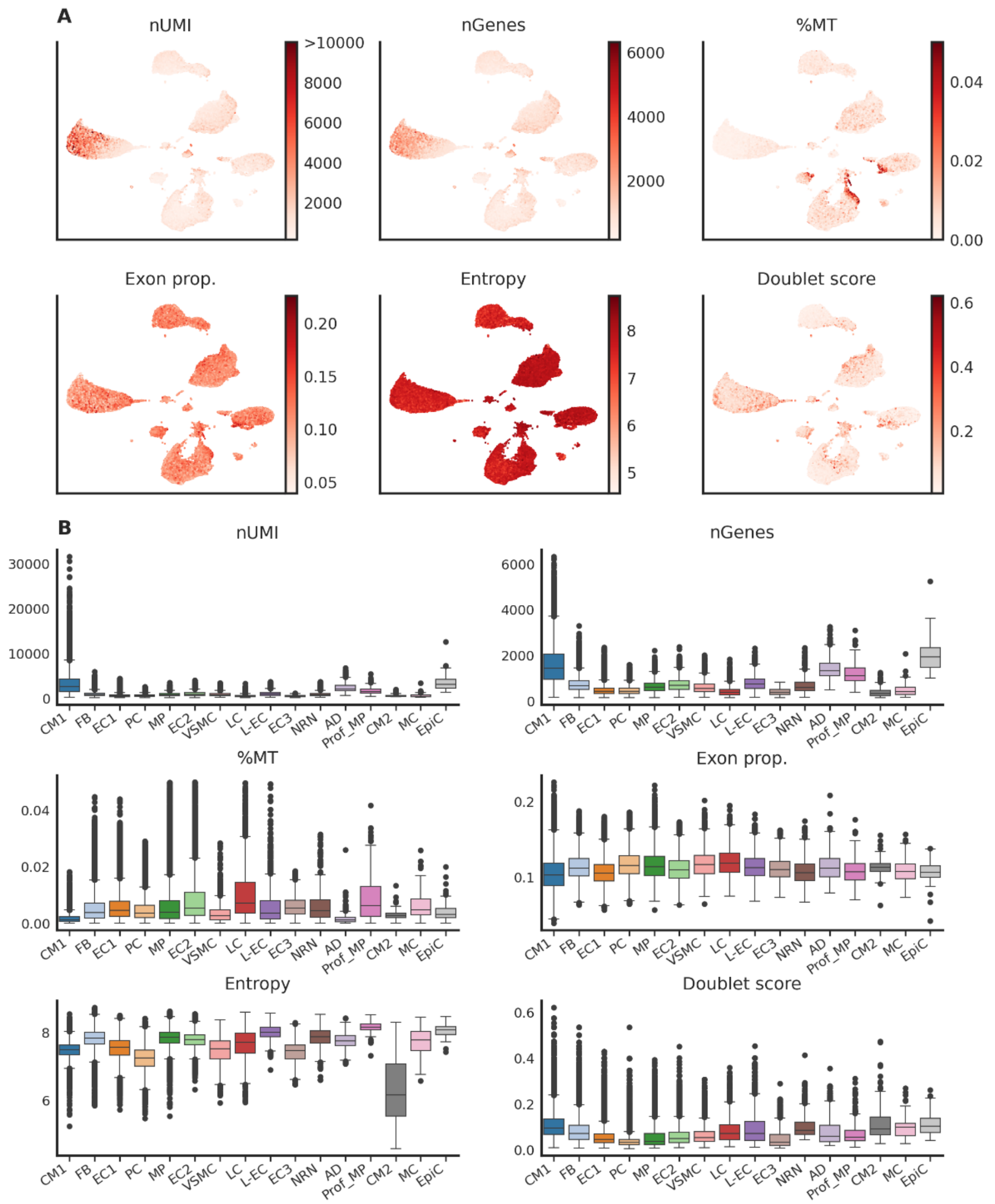

Supplemental Figure 1. Quality control metrics across final snRNA-seq map, related to Figure 1. **A,** Overlay of six per-nucleus quality control (QC) metrics on the final UMAP representation of the snRNA-seq data. QC metrics include the total number of unique molecular identifiers per nucleus (nUMI), the total unique genes detected per nucleus (nGenes), the percent of reads mapping to mitochondrial genes (%MT), the proportion of reads mapping exclusively to exons (Exon prop.), nucleus-level entropy (Entropy), and an estimated doublet score based on Scrublet (Doublet score). **B,** Distribution of the same QC metrics as in **A** stratified by cell type as shown in box plots represented as: Center line, median; Box limits, upper and lower quartiles; Whiskers, 1.5x interquartile range; Points, outliers. CM, cardiomyocyte; FB, fibroblast; EC, endothelial cell; PC, pericyte; MP, macrophage; VSMC, vascular smooth muscle cell; LC, lymphocyte; L-EC, lymphatic endothelial cell; NRN, neuronal; AD, adipocyte; Prof_MP, proliferating macrophage; MC, mast cell; EpiC, epicardial.

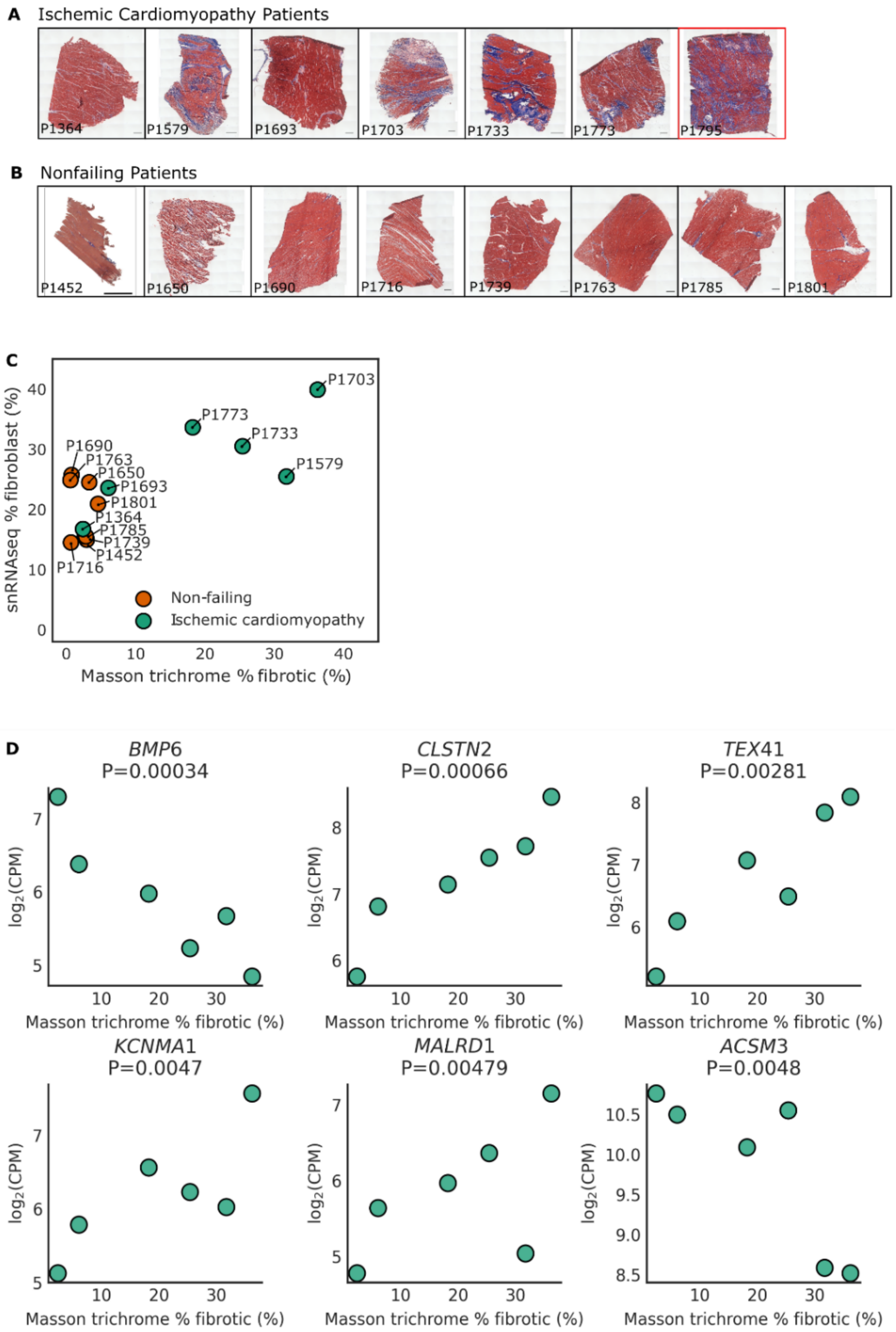

Supplemental Figure 2: LV sections from ICM patients show a wide range of fibrosis compared to NF controls, related to Figure 1. A, B. Mason's Trichrome staining of sections from patient tissue used for snRNAseq. Sample P1795 is denoted with a red border as this sample failed single nuclei RNA-sequencing. Blue: Collagen, Pink: tissue. Scale bar = 1 mm. **C**. Correlation of percentage of tissue section that stained blue with number of fibroblast nuclei from snRNAseq. **D.** Association of fibroblast gene expression with degree of fibrosis seen in Mason's Trichrome staining across 6 ICM patients. Genes with P < 0.005 are shown. CPM, counts per million.

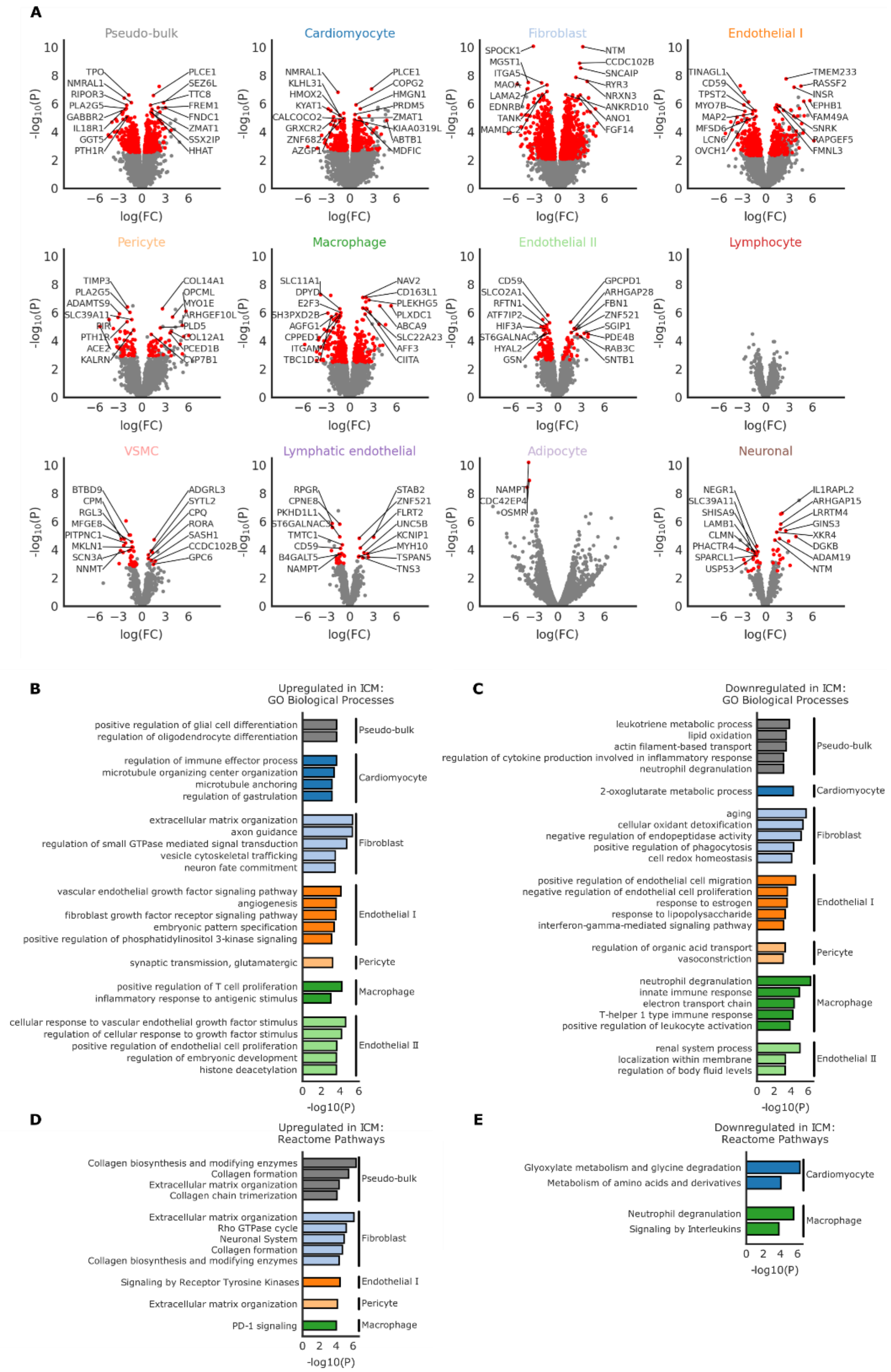
A
Pseudo-bulk
TPO
NMRAL1
RIPOR3
PLA2G5
GABBR2
IL18R1
GGT5
PTH1R
PLCE1
SEZ6L
TTC8
FREM1
FNDC1
ZMAT1
SSX2IP
HHAT
Cardiomyocyte
NMRAL1
KLHL31
HMOX2
KYAT1
CALCOCO2
GRXCR2
ZNF682
AZGP1
PLCE1
COPG2
HMGN1
PRDM5
ZMAT1
KIAA0319L
ABTB1
MDFIC
Fibroblast
SPOCK1
MGST1
ITGA5
MAOA
LAMA2
EDNRB
TANK
MAMDC2
NTM
CCDC102B
SNCAIP
RYR3
NRXN3
ANKRD10
ANO1
FGF14
Endothelial I
TINAGL1
CD59
TPST2
MYO7B
MAP2
MFSD6
LCN6
OVCH1
TMEM233
RASSF2
INSR
EPHB1
FAM49A
SNRK
RAPGEF5
FMNL3
Pericyte
TIMP3
PLA2G5
ADAMTS9
SLC39A11
RIR
PTH1R
ACE2
KALRN
COL14A1
OPCML
MYO1E
ARHGEF10L
PLD5
COL12A1
PCED1B
CYP7B1
Macrophage
SLC11A1
DPYD
E2F3
SH3PXD2B
AGFG1
CPPED1
ITGAM
TBC1D2
NAV2
CD163L1
PLEKHG5
PLXDC1
ABCA9
SLC22A23
AFF3
CIITA
Endothelial II
CD59
SLCO2A1
RFTN1
ATF7IP2
HIF3A
ST6GALNAC3
HYAL2
GSN
GPCPD1
ARHGAP28
FBN1
ZNF521
SGIP1
PDE4B
RAB3C
SNTB1
Lymphocyte
VSMC
BTBD9
CPM
RGL3
MFGE8
PITPNC1
MKLN1
SCN3A
NNMT
ADGRL3
SYTL2
CPQ
RORA
SASH1
CCDC102B
GPC6
Lymphatic endothelial
RPGR
CPNE8
PKHD1L1
ST6GALNAC3
TMTC1
CD59
B4GALT5
NAMPT
STAB2
ZNF521
FLRT2
UNC5B
KCNIP1
MYH10
TSPAN5
TNS3
Adipocyte
NAMPT
CDC42EP4
OSMR
Neuronal
NEGR1
SLC39A11
SHISA9
LAMB1
CLMN
PHACTR4
SPARCL1
USP53
IL1RAPL2
ARHGAP15
LRRTM4
GINS3
XKR4
DGKB
ADAM19
NTM
$-\log_{10}(P)$
log(FC)
B
Upregulated in ICM:
GO Biological Processes
positive regulation of glial cell differentiation
regulation of oligodendrocyte differentiation
Pseudo-bulk
regulation of immune effector process
microtubule organizing center organization
microtubule anchoring
regulation of gastrulation
Cardiomyocyte
extracellular matrix organization
axon guidance
regulation of small GTPase mediated signal transduction
vesicle cytoskeletal trafficking
neuron fate commitment
Fibroblast
vascular endothelial growth factor signaling pathway
angiogenesis
fibroblast growth factor receptor signaling pathway
embryonic pattern specification
positive regulation of phosphatidylinositol 3-kinase signaling
Endothelial I
synaptic transmission, glutamatergic
Pericyte
positive regulation of T cell proliferation
inflammatory response to antigenic stimulus
Macrophage
cellular response to vascular endothelial growth factor stimulus
regulation of cellular response to growth factor stimulus
positive regulation of endothelial cell proliferation
regulation of embryonic development
histone deacetylation
Endothelial II
-log10(P)
C
Downregulated in ICM:
GO Biological Processes
leukotriene metabolic process
lipid oxidation
actin filament-based transport
regulation of cytokine production involved in inflammatory response
neutrophil degranulation
Pseudo-bulk
2-oxoglutarate metabolic process
Cardiomyocyte
aging
cellular oxidant detoxification
negative regulation of endopeptidase activity
positive regulation of phagocytosis
cell redox homeostasis
Fibroblast
positive regulation of endothelial cell migration
negative regulation of endothelial cell proliferation
response to estrogen
response to lipopolysaccharide
interferon-gamma-mediated signaling pathway
Endothelial I
regulation of organic acid transport
vasoconstriction
Pericyte
neutrophil degranulation
innate immune response
electron transport chain
T-helper 1 type immune response
positive regulation of leukocyte activation
Macrophage
renal system process
localization within membrane
regulation of body fluid levels
Endothelial II
-log10(P)
D
Upregulated in ICM:
Reactome Pathways
Collagen biosynthesis and modifying enzymes
Collagen formation
Extracellular matrix organization
Collagen chain trimerization
Pseudo-bulk
Extracellular matrix organization
Rho GTPase cycle
Neuronal System
Collagen formation
Collagen biosynthesis and modifying enzymes
Fibroblast
Signaling by Receptor Tyrosine Kinases
Endothelial I
Extracellular matrix organization
Pericyte
PD-1 signaling
Macrophage
-log10(P)
E
Downregulated in ICM:
Reactome Pathways
Glyoxylate metabolism and glycine degradation
Metabolism of amino acids and derivatives
Cardiomyocyte
Neutrophil degranulation
Signaling by Interleukins
Macrophage
-log10(P)

S**upplemental Figure 3: Differentially expressed genes and pathway analysis of differentially expressed genes in ICM compared to NF, by cell type. Related to Figure 1. A** Volcano plot of differentially expressed genes in cell types, top 8 up- and down-regulated protein coding genes labelled. Genes significant at FDR < 0.05 with a low probability of being derived by background are colored red. **B** Pathway enrichment (P<0.001) for upregulated and downregulated (**C**) genes in ICM compared to NF based on gene ontology (GO) biological processes database using TopGO. **D** Reactome pathway enrichment (FDR<0.05) for upregulated and downregulated (**E**) genes in ICM compared to NF based on Reactome Pathways database using ReactomePA. log(FC), log fold-change; VSMC, vascular smooth muscle cell; ICM, Ischemic cardiomyopathy.

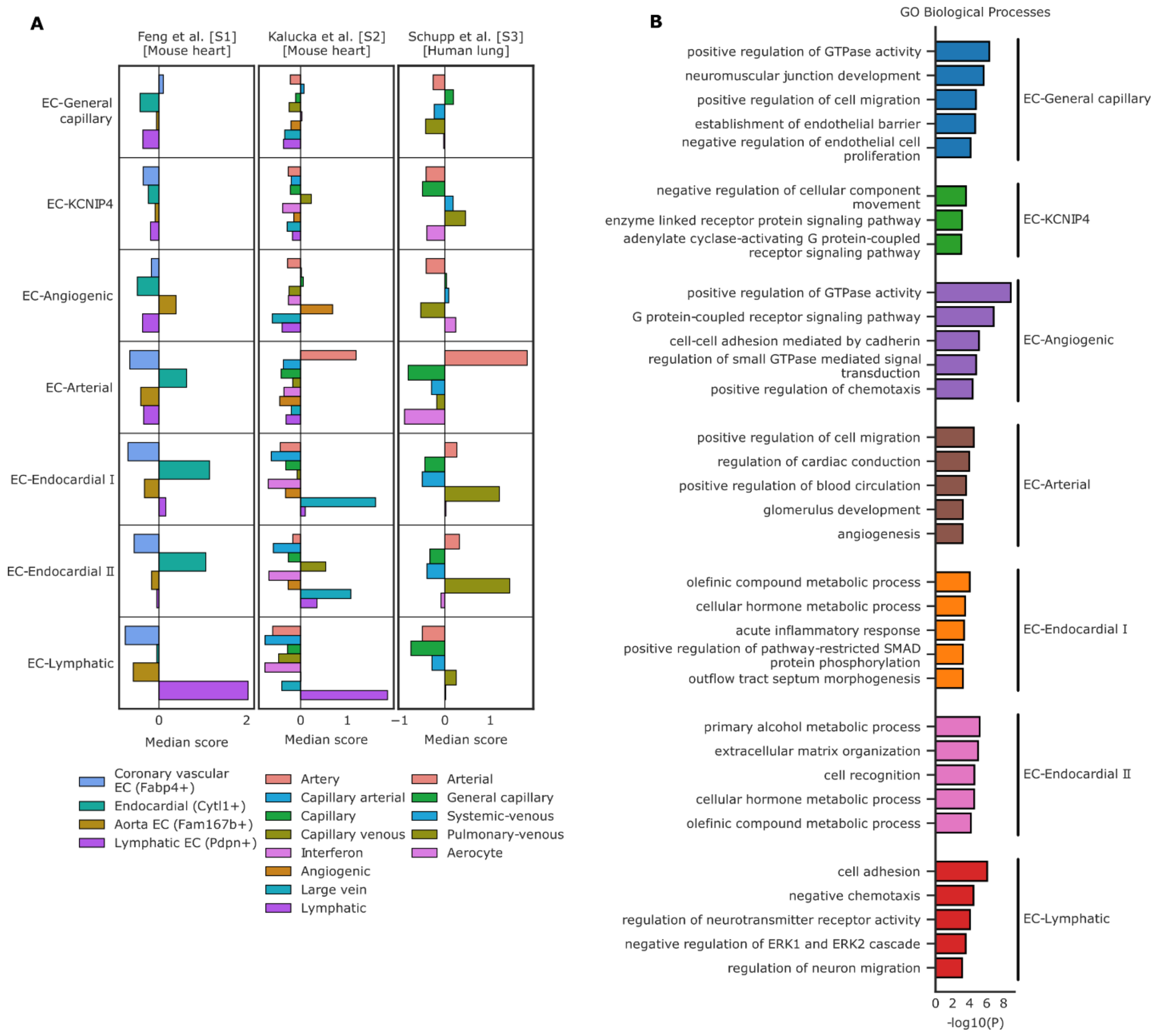

Supplemental Figure 4: Endothelial cell subcluster classification and pathway analysis, related to Figure 2. A. Assigned endothelial subtype classifications for six subclusters (EC-Angiogenic, EC-Arterial, EC-Endocardial I, EC-Endocardial II, EC-General Capillary, EC-Lymphatic) by comparing expression profiles to published data (see STAR Methods). **B**. Pathway analysis of significant marker genes of each endothelial cell subcluster using GO Biological Processes database. Only significant ontologies (P<0.001) are shown. EC, endothelial cell.

A

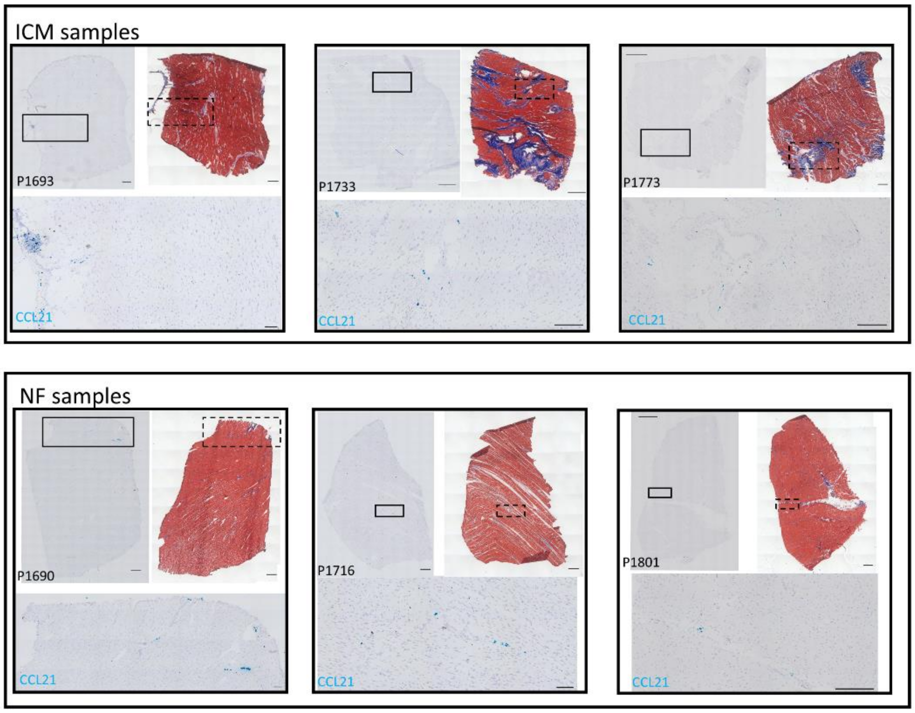
ICM samples
P1693
P1733
P1773
CCL21
CCL21
CCL21
NF samples
P1690
P1716
P1801
CCL21
CCL21
CCL21

B

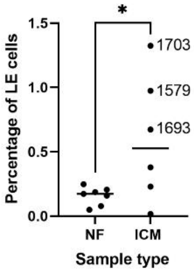
*
1.5
1.0
0.5
0.0
Percentage of LE cells
1703
1579
1693
NF
ICM
Sample type

C

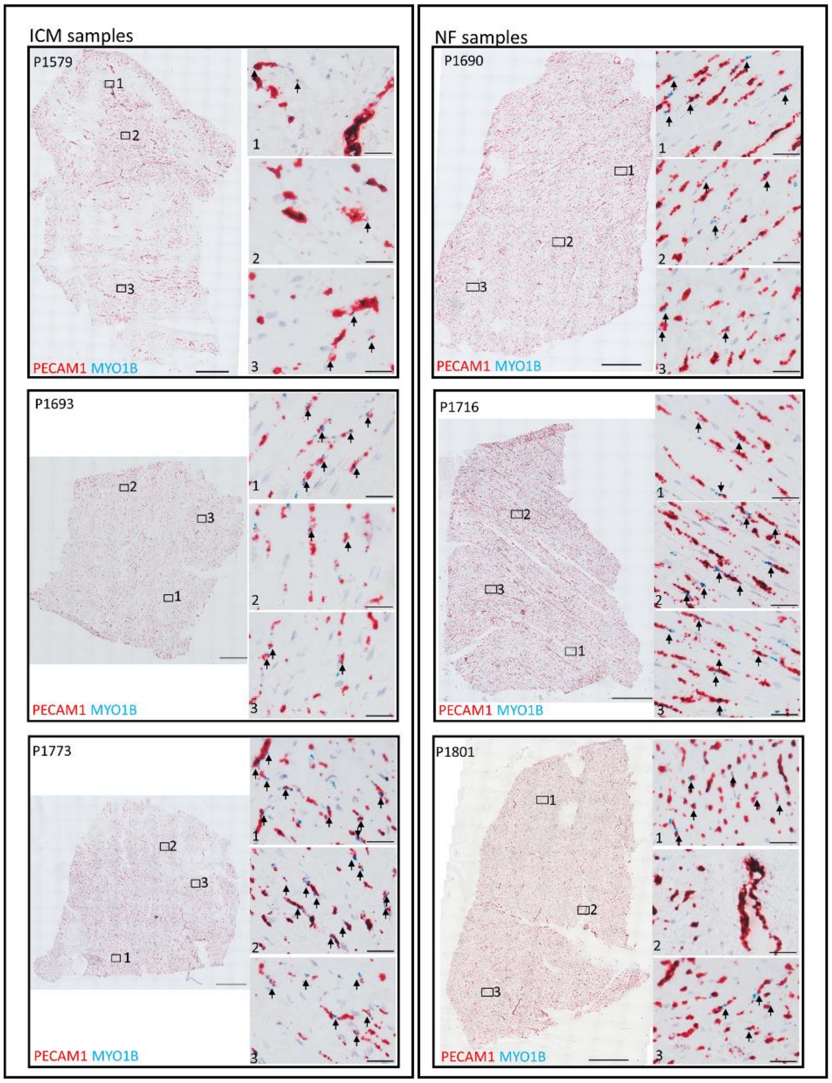
ICM samples
NF samples
P1579
P1690
P1693
P1716
P1773
P1801
1
2
3
PECAM1 MYO1B
PECAM1 MYO1B
PECAM1 MYO1B
PECAM1 MYO1B
PECAM1 MYO1B
PECAM1 MYO1B

Supplemental Figure 5: Example images of expanded in situ hybridization validation of EC-lymphatic subcluster and EC-angiongenic subcluster in three ischemic cardiomyopathy patients and three non-failing controls, related to Figure 2. **A** In situ hybridization with RNAscope showing localization of a canonical lymphatic endothelial cell marker, *CCL21* (blue) across sections from ischemic cardiomyopathy patients (P1693, P1733, P1773) and non-failing patients (P1690, P1716, P1801). Boxes on tile images show where the close-up images were taken, and dotted boxes show the corresponding region on the sequential trichrome stained sections. Nuclear localization is shown with hematoxylin (blue). **B** Quantification of RNAscope, percentage of *CCL21* positive cells over all nuclei, n = 7 (NF) and 6 (ICM), p = 0.0363. Line notes the median, *p<0.05, unpaired t-test. **C** In situ hybridization with RNAscope showing localization of a canonical endothelial marker *PECAM1* (red) and marker of EC-angiogenic subcluster *MYOB1* (blue) across sections from ischemic cardiomyopathy patients (P1579, P1693, P1773) and non-failing patients (P1690, P1716, P1801). Boxes on tile images show where the close-up images were taken, and arrows show double positive cells. Nuclear localization is shown with hematoxylin (blue). ICM, ischemic cardiomyopathy, NF, non-failing. Scale bars of tile represent 1 mm, and of close up of 250 um (A) or 50 um (C).

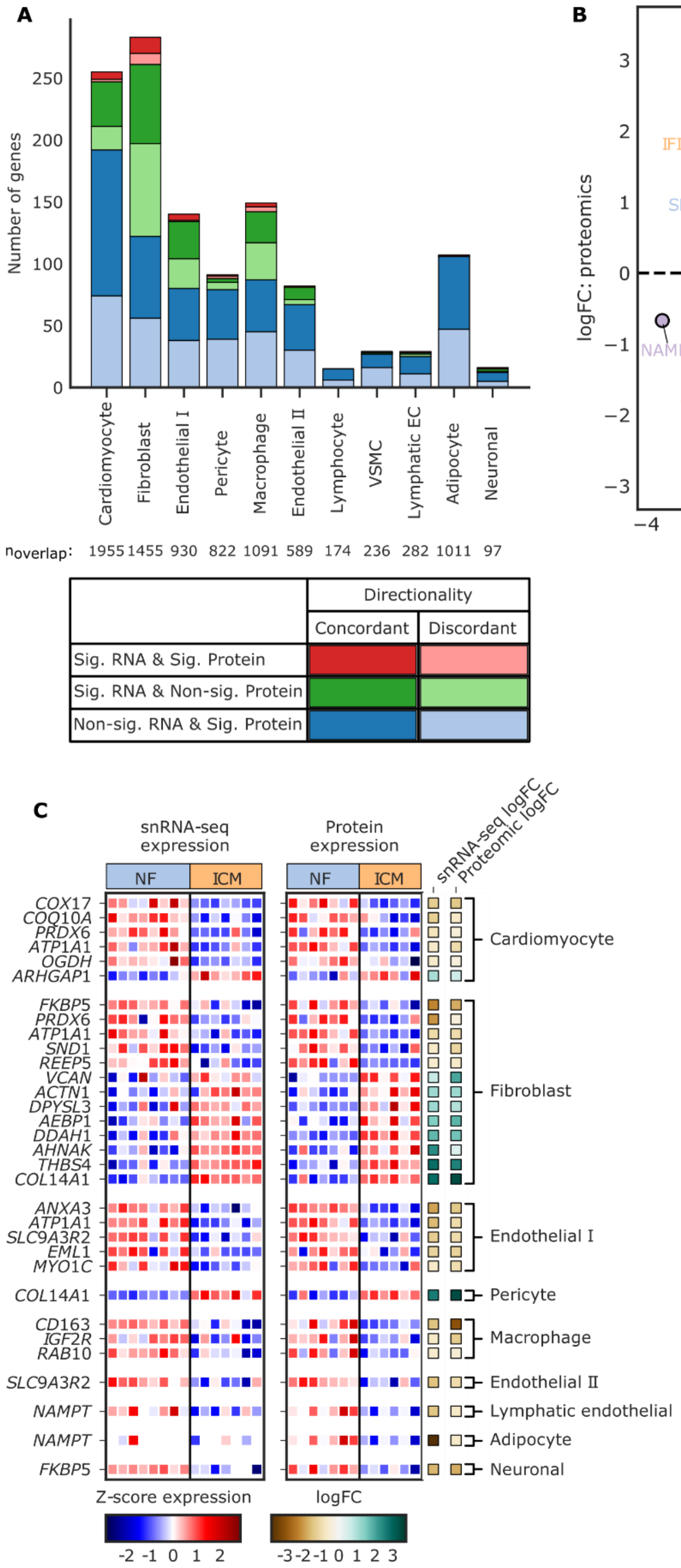
A
Number of genes
0
50
100
150
200
250
Cardiomyocyte
Fibroblast
Endothelial I
Pericyte
Macrophage
Endothelial II
Lymphocyte
VSMC
Lymphatic EC
Adipocyte
Neuronal
noverlap: 1955 1455 930 822 1091 589 174 236 282 1011 97
Directionality
Concordant
Discordant
Sig. RNA & Sig. Protein
Sig. RNA & Non-sig. Protein
Non-sig. RNA & Sig. Protein
B
logFC: proteomics
logFC: snRNA-seq
−4
−2
0
2
4
−3
−2
−1
0
1
2
3
COL14A1
COL14A1
THBS4
VCAN
AEBP1
DDAH1
ACTN1
DPYSL3
AHNAK
ARHGAP1
ALDH5A1
HIBADH
VCAN
PRELP
TSPAN14
C1QB
UQCR11
IFIT3
MFGE8
MFGE8
SERPINF1
A2M
SERPING1
C3
FBLN5
AZGP1
MFGE8
SERPINF2
ERP44
PRDX6
COQ10A
PRDX6
OGDH
RAB10
NAMPT
REEP5
SLC9A3R2
NAMPT
SLC9A3R2
MYO1C
ATP1A1
ATP1A1
SND1
EML1
ANXA3
ATP1A1
FKBP5
IGF2R
COX17
FKBP5
CD163
Cardiomyocyte
Fibroblast
Endothelial I
Pericyte
Macrophage
Endothelial II
VSMC
Lymphatic EC
Adipocyte
Neuronal
C
snRNA-seq expression
Protein expression
NF
ICM
NF
ICM
snRNA-seq logFC
Proteomic logFC
COX17
COQ10A
PRDX6
ATP1A1
OGDH
ARHGAP1
Cardiomyocyte
FKBP5
PRDX6
ATP1A1
SND1
REEP5
VCAN
ACTN1
DPYSL3
AEBP1
DDAH1
AHNAK
THBS4
COL14A1
Fibroblast
ANXA3
ATP1A1
SLC9A3R2
EML1
MYO1C
Endothelial I
COL14A1
Pericyte
CD163
IGF2R
RAB10
Macrophage
SLC9A3R2
Endothelial II
NAMPT
Lymphatic endothelial
NAMPT
Adipocyte
FKBP5
Neuronal
Z-score expression
-2 -1 0 1 2
logFC
-3 -2 -1 0 1 2 3

Supplemental Figure 6: Correlation of snRNAseq data to proteomics data, related to Figure 1. A. Bar plot showing the number and proportion of overlap of significantly dysregulated genes from snRNAseq or proteomics in ICM. Bar colors indicate the statistical significance and consistency of the directionality between the proteomics and snRNAseq analyses. The total number of genes/proteins tested in the given cell type in the snRNAseq as well as in the bulk proteomics data is listed below each bar ($n_{overlap}$) **B**. Plot of correlation of differential RNA/protein expression results between ICM and NF based on snRNAseq and proteomic data for genes that were significantly differentially expressed in both datasets. The color of each dot represents the cell type of the snRNAseq differential expression test. **C.** Heatmap of effect size estimates for protein coding genes that were significantly differentially expressed between ischemic cardiomyopathy (ICM) and non-failing (NF) in snRNAseq data (FDR-adjusted P-value < 0.05, expressed in > 1% of nuclei from either group, low background probability) and proteomic data. Blue/red shading represents Z score expression, Tan/Green shading represents the overall log fold-change comparing ICM to NF (logFC). logFC, log fold-change; VSMC, vascular smooth muscle cell.

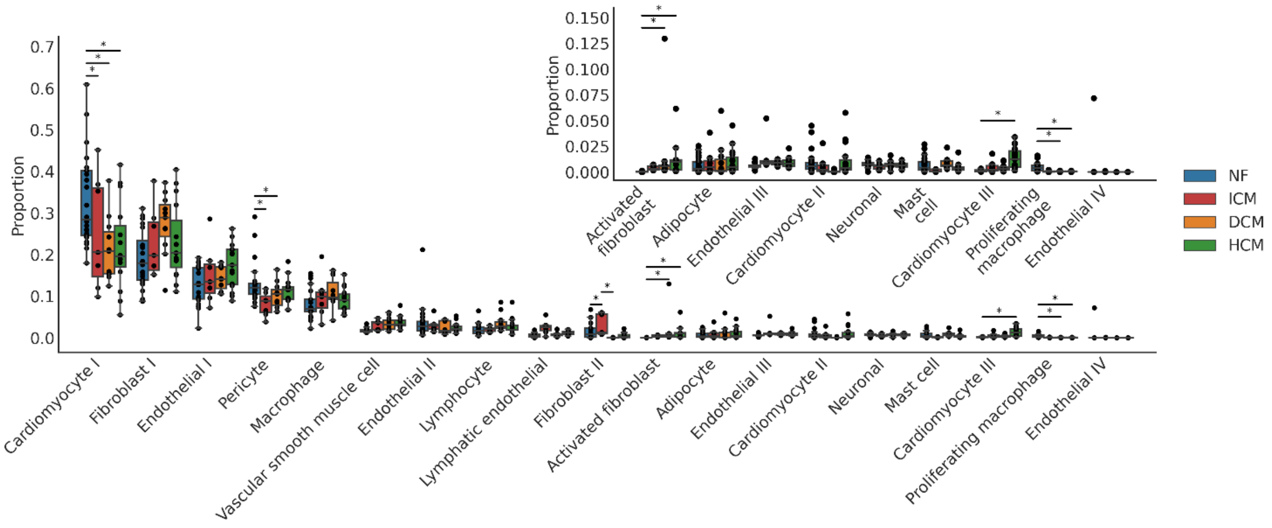

Supplemental Figure 7: Distribution of Cellular Composition in HCM/DCM/ICM combined dataset, related to Figure 5. Statistically credible changes are noted with a * (see STAR Methods). Box plots are represented as: Center line, median; Box limits, upper and lower quartiles; Whiskers, 1.5x interquartile range; Points, outliers.

Supplemental References

[S1] Feng, W., Chen, L., Nguyen, P.K., Wu, S.M., and Li, G. (2019). Single Cell Analysis of Endothelial Cells Identified Organ-Specific Molecular Signatures and Heart-Specific Cell Populations and Molecular Features. Front. Cardiovasc. Med. *6*, 165. 10.3389/fcvm.2019.00165.

[S2] Kalucka, J., de Rooij, L.P.M.H., Goveia, J., Rohlenova, K., Dumas, S.J., Meta, E., Conchinha, N.V., Taverna, F., Teuwen, L.-A., Veys, K., et al. (2020). Single-Cell Transcriptome Atlas of Murine Endothelial Cells. Cell *180*, 764-779.e20. 10.1016/j.cell.2020.01.015.

[S3] Schupp, J.C., Adams, T.S., Cosme, C., Raredon, M.S.B., Yuan, Y., Omote, N., Poli, S., Chioccioli, M., Rose, K.-A., Manning, E.P., et al. (2021). Integrated Single-Cell Atlas of Endothelial Cells of the Human Lung. Circulation *144*, 286–302. 10.1161/CIRCULATIONAHA.120.052318.

[S4] Zhang, Y., El-Sikhry, H, Chaudhary K. R., Batchu S. N., Shayeganpour, A., Jukar, T. B., Bradbury, J. A., Graves, J. P., DeGraff, L. M., Myers, P., et al (2009). Overexpression of CYP2J2 provides protection against doxorubicin-induced cardiotoxicity. Am J. Physiol Heart Circ Physiol. *297*: H37-46. 10.1152/ajpheart.00983.2008

[S5] Wang, B., Zeng, H., Wen, Z., Chen, C., Wang, D. W. (2016). CYP2J2 and its metabolites (epoxyeicosatreinoic acids) attenuates cardiac hypertrophy by activating AMPKa2 and enhancing nuclear translocation of Akt1. Aging Cell. *15*:940-952. 10.1111/acel.12507

[S6] Aliwarga, T., Evangelista, E. A., Sotoodehnia, N., Lemaitre, R. N., Totah, R. A. (2018) Regulation of CYP2J2 and EET levels in Cardiac Disease and Diabetes. Int. J. Mol. Sci *29* 1916 10.3390/ijms19071916

[S7] Valencia, R., Bassiouni, W., Darwesh, A. M., Bapuji, R., Seubert, J. M. (2022) Cardiomyocyte-specific CYP2J2 and its therapeutic implications. Expert Opin Drug Metab Toxicol. *18*: 423-439 10.1080/17425255.2022.2114344

[S8] Gil-Cayuela, C., Rosello-Lleti, E., Ortega, A., Tarazon, E., Trivino, J. C., Martinez-Dolz, L., Gonzalez-Juanatey, J. R., Lago, F., Portoles, M., Rivera M. (2016) New Altered Non-Fibrillar Collagens in Human Dilated Cardiomyopathy: Role in the Remodeling Process. PLoS One *11*: e0168130 10.1371/journal.pone.0168130

[S9] Galindo, C. L., Kasasbeh, E., Murphey, A., Ryzhov, S., Lenihan, S., Ahmad, F. A., Williams, P., Nunnally, A., Adcock, J., Song, Y., et al. (2014) Anti-remodeling and anti-fibrotic effects of the neuregulin-1b glial growth factor 2 in a large animal model of heart failure. JAHA. *3*: e000773 10.1161/JAHA.113.000773

[S10] Holm, H., Gudbjartsson, D. F., Sulem P., Masson, G., Helgadottir, H. T., Zanon, C, Magnusson, O. H., Helgason, A., Saemundsdottir, J., Gylfason, A., et al. (2011). A rare variant in MYH6 is associated with high risk of sick sinus syndrome. Nat. Genet. *43*: 316-320 10.1038/ng.781

[S11] Theis, J. L., Zimmermann, M. T., Evans, J. M., Eckloff, B. W., Wieben, E. D., Qureshi, M. Y., O'Leary, P. W., Olson T. M. (2015). Recessive MYH6 mutations in hypoplastic left heart with reduced ejection fraction. Circ Genetics *8:* 564-571 10.1161/CIRCGENETICS.115.001070

[S12] Granados, J. T., Ghosh, T. K., Pople, M., Bu'Lock, F., Thornborough, C., Eason, J., Kirk, E. P., Fatkin, D., Feneley, M. P., Harvey, R. P., et al. (2010). a-Cardiac myosin heavy chain (MYH6) mutations affecting myofibril formation are associated with congenital heart defects. Human Molecular Genetics, *19*: 4007-4016 10.1093/hmg/ddq315

[S13] Jiang, J., Wakimoto, H., Seidman, J. G., Seidman, C. E. 2013. Allele-specific silencing of mutant Myh6 transcripts in mice suppresses hypertrophic cardiomyopathy. Science *342*:111-114 10.1126/science.1236921

[S14] Justo, T., Martiniuc, A., Dhoot, G. K. (2021) Modulation of cell signalling and sulfation in cardiovascular development and disease. Sci Rep. *11*: 22424 10.1038/s41598-021-01629-0

[S15] Korf-Klingebiel, M., Reboll, M. R., Grote, K., Schleiner, H., Wang, Y., Wu, X., Klede, S., Mikhed, Y., Bauersachs, J., Klintschar, M., et al. (2019) Heparan Sulfate-Editing Extracellular Sulfatases Enhance VEGF Bioavailability for Ischemic Heart Repair. Circ. Res. *11*: 787-801 10.1161/CIRCRESAHA.119.315023

[S16] Yue, X., Li, X., Nguyen H. T., Chin, D. R., Sullivan D. E, Lasky, J. A. (2008) Transforming growth factor-beta1 induces heparan sulfate 6-O-endosulfatase 1 expression in vitro and in vivo. J. Biol Chem *18*: 20397-407 10.1074/jbc.M802850200

[S17] Gerarduzzi, C., Kumar, R. K., Trivedi, P., Ajay, A. K., Iyer, A., Boswell, S., Hutchinson J. N., Waikar, S. S., Vaidya, V. S. (2017) Silencing SMOC2 ameliorates kidney fibrosis by inhibiting fibroblast to myofibroblast transformation. JCI Insight. *2*: e90299 10.1172/jci.insight.90299

[S18] Luo, L., Wang, C-C., Song, X-P., Wang, H-M., Zhou, H., Sun, Y., Wang, X-K., Hou, S., Pei, F-Y. (2018) Suppression of SMOC2 reduces bleomycin (BLM)-induced pulmonary fibrosis by inhibition of TGF-b1/SMADs pathway. *105*: 841-847 10.1016/j.biopha.2018.03.058

[S19] Yuting, Y., Lifeng, F., Qiwei, H. (2019) Secreted modular calcium-binding protein 2 promotes high fat diet (HFD)-induced hepatic steatosis through enhancing lipid deposition fibrosis and inflammation via targeting TGF-b1. Biochem Biophys Res Commun. *509*: 48-55 10.1016/j.bbrc.2018.12.006

[S20] Bergmeier, V., Etich, J., Pitzler, L., Frie, C., Koch, M., Fischer, M., Rappl, G., Abken, H., Tomasek, J. J., Brachvogel, B. (2018) Identification of a myofibroblast-specific expression signature in skin wounds. Matrix Biol. *65*: 59-74 10.1016/j.matbio.2017.07.005

[S21] Tao, G., Levay, A. K., Peacock, J. D., Huk, D., J., Both, S. N., Purcell, N. H., Pinto, J. R., Galantowicz, M. L, Koch, M., Lucchesi, P. A., et al. (2012) Collagen XIV is important for growth and structural integrity of the myocardium. J. Mol Cell Cardiol. *53*: 626-38 10.1016/j.yjmcc.2012.08.002

[S22] Ma, Z., Wang, X., LV, Q., Gong, Y., Xia, M., Zhuang, L., Lu, X., Yang, Y., Zhang, W., Fu, G., et al. (2021) Identification of underlying hub genes associated with hypertrophic cardiomyopathy by integrated bioinformatics analysis. Pharmogenomics Pers Med. *14*: 823-837 10.2147/PGPM.S314880

[S23] Xie, T., Wang, Y., Deng, N., Huang, G., Taghavifar, F., Geng, Y., Liu, N., Kulur, V., Yao, C., Chen, P., et al. (2018) Single-cell deconvolution of fibroblast heterogeneity in mouse pulmonary fibrosis. Cell Rep. *22*: 3625-3640 10.1016/j.yjmcc.2012.08.002

[S24] Komatsu, M., Kanda, T., Urai, H., Kurokochi, A., Kitahama, R., Shigaki, S., Ono, T., Yukioka, H., Hasegawa, K., Tokuyama, H., et al. (2018) NNMT activation can contribute to the development of fatty liver disease by modulating the NAD+ metabolism. Sci Rep. *8*:8637 10.1038/s41598-018-26882-8

[S25] Takahashi, R., Kanda, T., Komatsu, M., Itoh, T., Minakuchi, H., Urai, H., Kuroita, T., Shigaki, S., Tsukamoto, T., Higuchi, N., et al. (2022) The significance of NAD+ metabolites and nicotinamide N-methyltransferase in chronic kidney disease. Sci Rep *12*: 6398 10.1038/s41598-022-10476-6

[S26] Samuchiwal, S. K., Balestrieri, B. (2018) Harmful and protective roles of group V Phospholipase A2: current perspectives and future directions. Biochim Biophys Acta Mol Cell Biol Lipids. *1864*: 819-826 10.1016/j.bbalip.2018.10.001

[S27] Zhang, Y., Fu, C., Zhao, S., Jiang, H., Li, W., Liu X. (2022) PRELP promotes myocardial fibrosis and ventricular remodelling after acute myocardial infarction by the wnt/b-catenin signalling pathway. Cardiovasc J Afr. *33*:1-6 10.5830/CVJA-2022-001

[S28] Chacon-Solano, E., Leon, C., Carretero, M., Garcia, M., Sanchez-Dominguez, R., Quero, F., Mendez-Jimenez, E., Bonafont, J., Ruiz-Mezcua, B., Escamez, M. J., et al. (2022) Mechanistic interrogation of mutation-independent disease modulators of RDEB identifies the small leucine-rich proteoglycan PRELP as a TGF-b antagonist and inhibitor of fibrosis. *111*:189-206 10.1016/j.matbio.2022.06.007

[S29] Du, Z., Lin, Z., Wang, Z., Liu, D., Tian, D., Xia, L. (2020) SPOCK1 overexpression induced by platelet-derived growth factor-BB promotes hepatic stellate cell activation and liver fibrosis through the interin a5b1/PI3K/Akt siganling pathway. Lab Invest. *100:* 1042-1056 10.1038/s41374-020-0425-4

[S30] Singh, P., Rai, A., Dohare, R., Arora, S., Ali, S., Parveen, S., Syed, M. A. (2020) Network-based identification of signature genes KLF6 and SPOCK1 associated with oral submucous fibrosis. Mol Clin Oncol. *12*:299-310 10.3892/mco.2020.1991

[S31] Kolur, V., Vastrad, B., Bastrad, C., Kotturshetti, S., Tengli, A. (2021) Identification of candidate biomarkers and therapeutic agents for heart failure by bioinformatics analysis. BMC Cardiovasc Disord. *21*: 329 10.1186/s12872-021-02146-8

[S32] di Salvo, T., G., Yang, K-C., Brittain, E., Absi, T., Maltais, S., Hemnes, A. (2015) Right ventricular myocardial biomarkers in human heart failure. J. Card. Fail. *21*:398-411 10.1016/j.cardfail.2015.02.005

[S33] Liu, B., Xiang, L., Ji, J., Liu, W., Chen, Y., Xia, M., Liu, Y., Liu, W., Zhu, P., Jin, Y., et al. (2021) SPARCL1 promotes nonalcoholic steatohepatitis progression in mice through upregulation of CCL2. J. Clin. Invest. *131*:e144801 10.1172/JCI144801

[S34] Ye, H., Wang, W-G., Cao, J., Hu, X-C. (2017) SPARCL1 suppresses cell migration and invasion in renal cell carcinoma. Mol Med Rep. *16*:7784-7790 10.3892/mmr.2017.7535

[S35] Salmasi, M., Y., Morris-Reosendahl, D., Jarral, O. A., Rosendahl, U., Asimakopoulos, G., Raja, S., Aragon-Martin, J. A., Child, A., Pepper, J., et al. London Aortic Mechanobiology Working Group. (2022) Determining the genetic contribution in patients with non-syndromic ascending thoracic aortic anuerysms: Correlation with findings from computational pathology. Int. J. Cardiol. *1*:1-9 10.1016/j.ijcard.2022.07.010

[S36] Zhang, R., Xu, X., Chen, X., Hao, C., Ji, Z., Zuo, P., Yang, M., Ma, Genshan, M., Li, Y. (2022) Upregulation of key genes ELN and TGFB3 were association with the severity of cardiac hypertrophy. BMC Genomics. *14*:592 10.1186/s12864-022-08778-0

[S37] de Brouwer, B., Drent, M., van den Ouweland, J. M. W., Wignen, P. A., van Moorsel, C. H. M., Bekers, O., Crutters, J. C., White, E. S., Janssen, R. (2018) Increased circulating desmosine and age-dependent elastinolysis in idiopathic pulmonary fibrosis. Respir. Res. *19*:45 10.1186/s12931-018-0747-6
